# IFN-**γ** and donor leukocyte infusions for relapsed myeloblastic malignancies after allogeneic hematopoietic stem cell transplantation

**DOI:** 10.1172/jci.insight.190655

**Published:** 2025-03-25

**Authors:** Sawa Ito, Emily Geramita, Kedwin Ventura, Biswas Neupane, Shruti Bhise, Erika M. Moore, Scott Furlan, Warren D. Shlomchik

**Affiliations:** 1 Division of Hematology-Oncology, Department of Medicine;; 2 Thomas E. Starzl Transplantation Institute; and; 3 Department of Immunology, University of Pittsburgh, Pittsburgh, Pennsylvania, USA.; 4 Department of Pediatrics, University of Washington School of Medicine, Fred Hutchinson Cancer Center, Seattle, Washington, USA.; 5 Department of Pathology, University of Pittsburgh, Pittsburgh, Pennsylvania, USA.

**Keywords:** Hematology, Transplantation, Cancer, Cytokines, Immunotherapy

## Abstract

**BACKGROUND:**

The graft-versus-leukemia (GVL) effect contributes to the efficacy of allogeneic stem cell transplantation (alloSCT). However, relapse, indicative of GVL failure, is the greatest single cause of treatment failure. Based on preclinical data showing that IFN-γ is important to sensitize myeloblasts to alloreactive T cells, we performed a phase I trial of IFN-γ combined with donor leukocyte infusions (DLIs) in myeloblastic malignancies that relapsed after HLA-matched alloSCT.

**METHODS:**

Patients with relapsed acute myeloid leukemia or myelodysplastic syndrome after alloSCT were eligible. Patients self-administered IFN-γ for 4 weeks (cohort 1) or 1 week (cohort 2), followed by DLI and concurrent IFN-γ for a total of 12 weeks. Bone marrow samples were analyzed by single-cell RNA sequencing (scRNA-Seq) to assess in vivo responses to IFN-γ by malignant myeloblasts.

**RESULTS:**

IFN-γ monotherapy was well tolerated by all participants (*n* = 7). Treatment-related toxicities after DLI included grade I–II graft-versus-host disease (*n* = 5), immune effector cell–associated neurotoxicity syndrome (*n* = 2), and idiopathic pulmonary syndrome (*n* = 1), all of which resolved with corticosteroids. Four of 6 DLI recipients achieved minimal residual disease-negative complete remissions and full donor hematopoietic recovery. Median overall survival was 579 days (range, 97–906) in responders. scRNA-Seq validated in vivo activation of the IFN-γ response pathway in hematopoietic stem cell–like or myeloid progenitor cells after IFN-γ in analyzed samples.

**CONCLUSION:**

IFN-γ was safe and well tolerated in this phase I study of IFN-γ for relapsed acute myeloid leukemia and myelodysplastic syndrome after alloSCT, with a promising efficacy signal when combined with DLI. Larger studies are needed to formally test the efficacy of this approach.

**TRIAL REGISTRATION:**

ClinicalTrials.gov NCT04628338.

**FUNDING:**

UPMC Hillman Cancer Center Cancer Immunology and Immunotherapy Program Pilot Award and Cure Within Reach: Drug Repurposing Clinical Trials to Impact Blood Cancers.

## Abstract 2

This phase I study demonstrated the safety and potential efficacy of IFN-γ for the treatment of relapsed AML/MDS after allogeneic stem cell transplantation.

## Introduction

Allogeneic stem cell transplantation (alloSCT) can be a curative treatment for patients with hematologic malignancies, including acute myeloid leukemia (AML) and myelodysplastic syndrome (MDS), which comprise approximately 60% of alloSCTs performed for malignant diseases in the United States. Part of the efficacy of alloSCT is due to donor-derived alloreactive αβ T cells that can recognize leukemia cells as non-self, thereby mediating the graft-versus-leukemia (GVL) effect (1). However, relapse of primary disease, indicative of GVL failure, occurs in 30%–40% of patients and remains the single most common cause of death in this patient population (2–5). It is estimated that approximately 2,000 alloSCT recipients with underlying AML/MDS develop posttransplant relapse annually, and 80% of those die from relapse within 2 years (5–7).

There is no consensus strategy for treating AML or MDS that has relapsed after alloSCT. Salvage chemotherapy regimens spanning a range of intensities are commonly employed. A low-intensity approach with hypomethylating agent monotherapy achieves only a 9%–20% complete remission (CR) rate, mostly short-lived, with a median survival of only 3–6 months (8–15). Combining a hypomethylating agent with the B cell lymphoma 2 inhibitor, venetoclax, yields an 18%–28% CR rate, mostly of a short duration, with a disappointing median survival of less than 6 months (16–20). More intense multiagent regimens, for example, those containing fludarabine, cytarabine, and idarubicin, without or with venetoclax, have been used for younger and fit patients. However, even these regimens yield a CR in only up to 36% of participants, again of a short duration, leading to a poor median survival of 6 months (16, 21–23). These cytotoxic therapies can also cause end organ damage and prolonged cytopenias, resulting in frequent opportunistic infections. Consistent with this, high-dose chemotherapy was associated with a 10% early mortality rate from infection or multiorgan failure (16).

Chemotherapy can also be used to cytoreduce and to lymphodeplete before a donor leukocyte infusion (DLI) or as a bridge to a second alloSCT. However, DLI or second alloSCT durably salvages fewer than 20% of patients (5, 6, 24, 25). Novel approaches to treat posttransplant relapse are needed.

The GVL effect is not equally potent against all types of leukemias. For example, chronic-phase chronic myelogenous leukemia (CP-CML) is exquisitely GVL sensitive, whereas blast crisis CML (BC-CML) is GVL resistant, despite sharing common biology (24–27). Likewise, AML and acute lymphoblastic leukemia are relatively GVL resistant (28). In the recent CTN1301 trial (29) comparing different approaches of graft-versus-host disease (GVHD) prevention combined with myeloablative conditioning in patients with AML, the relapse rates were similar in the CD34-selected arm as compared to arms that received T cell–replete grafts, highlighting the relative GVL resistance of AML, the disease for which alloSCT is most commonly employed.

To better understand GVL resistance, we previously established GVL models against mouse CP-CML (mCP-CML) and mouse BC-CML (mBC-CML) created by retroviral transfer into bone marrow (BM) cells of disease-defining oncogenes (30–32). As is the case in the clinic, mCP-CML is very GVL sensitive, whereas mBC-CML is relatively GVL resistant. Gene-deficient leukemias were then created to screen for mechanisms of GVL resistance. The key finding was that mBC-CML required the expression of the IFN-γ receptor (IFN-gR) for optimal CD4- and CD8-mediated GVL. In contrast, *STAT1/STAT2*^–*/*–^ mCP-CML, which cannot respond to any type of IFN, was fully GVL sensitive (32). Similarly, *IFN-gR*^–*/*–^ MLL-AF9 AML was also relatively GVL resistant. These results suggest that optimal GVL against myeloblastic leukemias may require a high-magnitude, alloreactive T cell response that generates IFN-γ whereas a low-level, smoldering, alloreactive T cell response is sufficient to control CP-CML.

Compelling clinical data also support an important role for IFN-γ in sensitizing myeloblasts to alloreactive T cell killing. Lower rates of AML relapse were reported in patients who developed early CMV reactivation, which induces NK cell– and T cell–derived IFN-γ release (33–37). Two independent groups described lower levels of human leukocyte antigen (HLA) expression in myeloblasts at posttransplant relapse relative to HLA expression in pretransplant specimens. Notably, both found that IFN-γ restored HLA expression on relapsed myeloblasts in vitro (38, 39). Another cause of GVL failure is T cell exhaustion (39–46). A defining characteristic of exhausted T cells is that they produce little IFN-γ, which, as described above, sensitizes myeloblasts to T cell killing. Taken together, these preclinical results argue for treating relapsed myeloblastic leukemias with a DLI as a source of nonexhausted T cells in combination with pharmacologic IFN-γ. IFN-γ is an FDA-approved therapy for chronic granulomatous disease but to our knowledge has never been tested as part of a strategy for treating post-alloSCT relapse. Here, we report safety and preliminary clinical and biological data from a phase I trial of IFN-γ in combination with DLI in treatment of relapsed AML or MDS after alloSCT.

## Results

### Treatment plan and patient characteristics.

**Figure 1:**
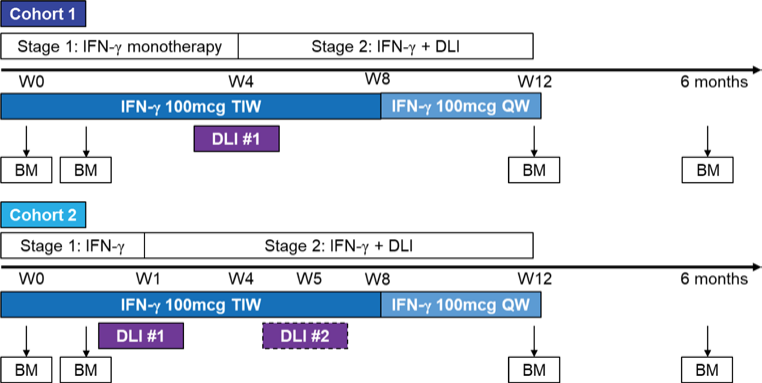
Clinical trial design. Treatment on the study was in 2 stages: (a) IFN-γ monotherapy and (b) IFN-γ with DLI. Research BM samples were collected before and 48–72 hours after the first or second dose of IFN-γ. In cohort 1, the first DLI was infused after 4 weeks of IFN-γ monotherapy. In cohort 2, the first DLI was infused after 1 week of IFN-γ monotherapy. BM, bone marrow; TIW, 3 times a week; QW, once a week.

We conducted a phase I trial of IFN-γ in combination with DLI in participants with relapsed AML/MDS after alloSCT (NCT04628338; Figure 1; clinical protocol in Supplemental Data 1; supplemental material available online with this article; https://doi.org/10.1172/jci.insight.190655DS1). Patients were eligible if they had relapsed AML or MDS after having undergone an HLA-matched alloSCT (for detailed enrollment criteria, see the full clinical protocol in Supplemental Data 1). We enrolled 2 patients with MDS and 2 patients with AML (patient characteristics, Table 1) in cohort 1, wherein IFN-γ was administered for 4 weeks as monotherapy prior to the first DLI containing 10^7^ CD3^+^ cells/kg. Three additional participants (all with AML; Table 1) were enrolled in a second cohort, wherein the duration of IFN-γ monotherapy was shortened to 1 week prior to DLI. This change was made to decrease the potential risk that patients would progress during the IFN-γ monotherapy period.

All patients had poor-risk cytogenetic or molecular features. One patient received a myeloablative HLA-matched sibling alloSCT whereas the other 6 patients received reduced-intensity HLA-matched unrelated transplants. The times from transplant to relapse (median 142 days, range 30–413 days), pre–IFN-γ treatment BM blasts, and donor myeloid or T cell chimerism varied (Table 1). Three out of 6 patients were refractory to salvage chemotherapy regimens before enrollment. One patient (patient 2) received only IFN-γ monotherapy as DLI was not available.

### Toxicities and safety.

All safety data and probable or possible adverse events are summarized in Table 2 and Supplemental Table 1. IFN-γ monotherapy was well tolerated in both cohorts, with only anticipated grade 1–2 flu-like symptoms. During IFN-γ monotherapy, the main grade 3 or higher adverse events were cytopenias, which were likely attributable to the underlying AML or MDS. One patient (patient 2) developed grade 3 neutropenic fever with bacteremia 28 days after the initiation of IFN-γ, coincident with rapid disease progression. Notably, no patients developed GVHD while receiving IFN-γ monotherapy.

Four of 6 DLI recipients developed steroid-sensitive, grade I–II acute GVHD between 21 and 73 days after the first DLI. The remaining 2 recipients did not develop GVHD; patient 5 received 2 DLIs and went off study to receive subsequent therapies (see below), and patient 7 died shortly after his first DLI due to rapid disease progression. Two patients (patients 3 and 6) developed ICANS 53 and 14 days after DLI, respectively, which resolved with corticosteroids. Patient 5, who did not manifest clinical GVHD after 2 DLIs, developed steroid-sensitive nephrotic syndrome with biopsy-proven minimal-change disease 73 days after his second DLI, coincident with disease progression. Patient 6 developed IPS 33 days after DLI, which resolved completely with systemic corticosteroids. The detailed clinical courses of the 2 ICANS cases are summarized in Supplemental Data 2.

### Clinical responses and survival.

**Figure 2:**
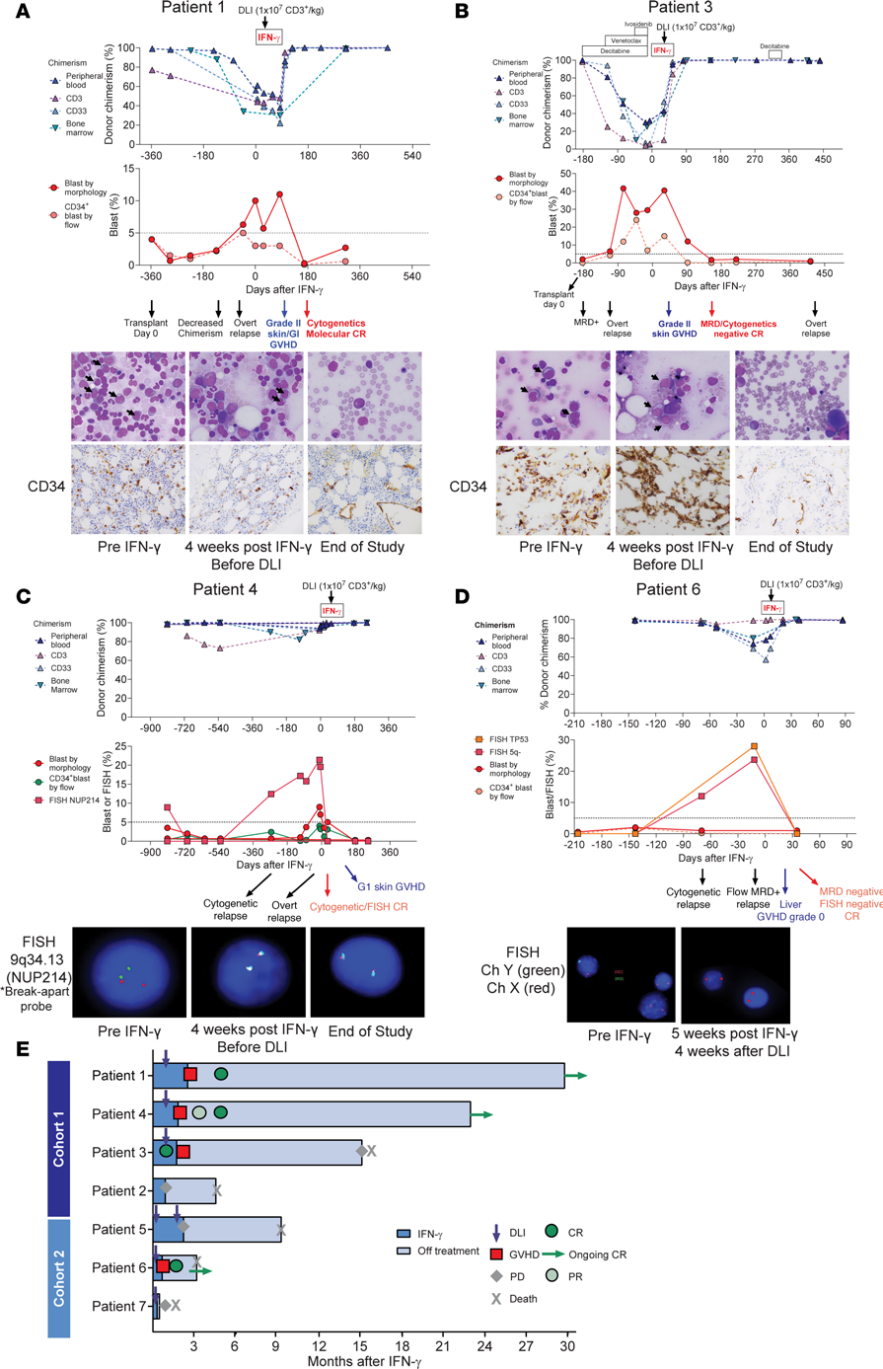
Clinical course of responders after initiation of IFN-γ. Panels **A**–**D** capture the courses of patients 1, 3, 4, and 6 (respectively), who achieved MRD^neg^ CRs. Day 0 marks the start of IFN-γ therapy. The top plot depicts the kinetics of donor chimerism in unfractionated blood and BM and in the CD3^+^ and CD33^+^ fractions in blood. The lower plots show leukemia quantitation based on blast percentages by morphology and the percentages of CD34^+^ cells by flow cytometry (results from the clinical hematopathology and flow cytometry labs). The second row shows May-Grunwald-Giemsa–stained BM slides. The arrows indicate representative malignant cells (**A** and **B**). The third row shows representative BM morphology with CD34 staining (**A** and **B**) or FISH analyses of 9q34.13 NUP214 translocation using a “break apart” probe approach (**C**) or immunofluorescence for the X and Y chromosomes (**D**). (**E**) Timelines of the courses of all study participants. CR, complete remission; PR, partial remission; PD, progressive disease.

In cohort 1, all 3 patients who received IFN-γ and a DLI achieved complete molecular, cytogenetic, and measurable residual disease–negative CRs (MRD^neg^ CRs) with conversion to full donor chimerism, coincident with the development of steroid-sensitive GVHD (Figure 2, A–C). These CRs were durable, resulting in a median survival of 903 days (range 460–990 and ongoing). Patient 4 (Figure 2C), with pretreatment donor T cell chimerism of 97%, achieved a CR documented by a negative FISH result for his disease-defining DEK-NUP214 translocation with IFN-γ monotherapy prior to his receiving a DLI (0 of 185 cells with the rearrangement). One patient (patient 2), who did not have available DLI and had poor donor T cell chimerism (8%), was treated with IFN-γ only and died from disease progression at day +139 after IFN-γ initiation.

In cohort 2, all patients received IFN-γ + DLI and had variable clinical responses. Patient 6 (Figure 2D), with complex-karyotype AML who received IFN-γ + DLI, achieved an MRD^neg^ CR and conversion to full donor chimerism, associated with steroid-sensitive GVHD (biopsy-proven liver, grade 0), IPS, and ICANS, which both resolved with corticosteroids. He remained with full donor chimerism without evidence of relapse until day +97, when he died from aspiration pneumonia. Patient 5 never developed GVHD even after 2 DLIs and went off study to receive additional salvage therapy. Patient 7 died from the rapid progression of disease associated with multiorgan failure 7 days after 1 DLI and 14 days after beginning IFN-γ.

In summary for patients in both cohorts, 4 out of 6 patients (66%) who received IFN-γ with a DLI achieved MRD^neg^ CRs coincident with steroid-sensitive GVHD. The clinical courses are summarized in Figure 2E.

### IFN-γ responsiveness of myeloblasts.

**Figure 3:**
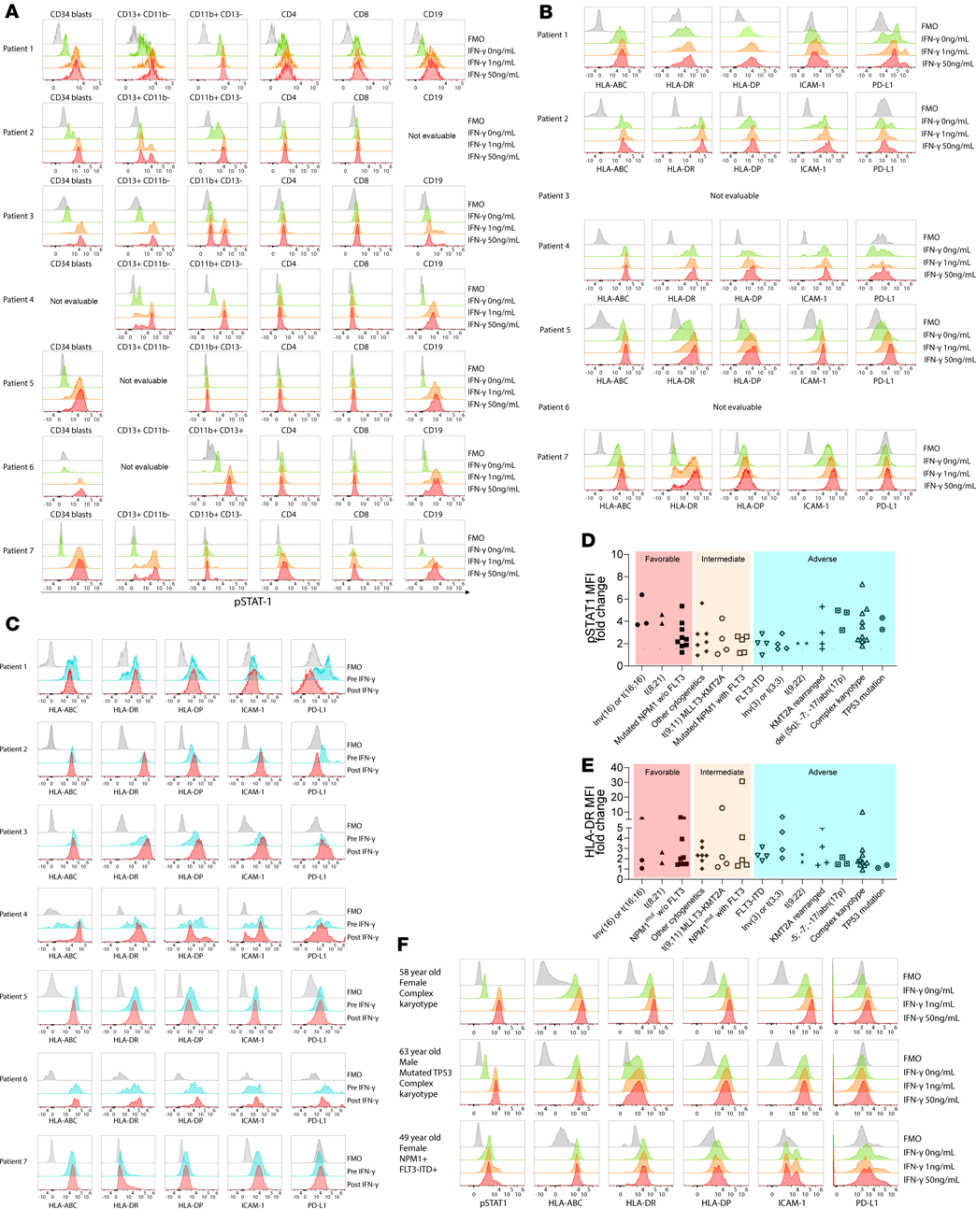
IFN-γ responsiveness of leukemia myeloblasts. In vitro p-STAT1 quantitation of BM cells after in vitro culture with or without IFN-γ (1 or 50 ng/mL) for 15 minutes (**A**). Shown is anti–p-STAT1 staining gating on cells that were CD34^+^, CD13^+^CD11b^–^, CD4^+^, CD8^+^, or CD19^+^. (**B**) Expression of HLA-ABC, HLA-DR, HLA-DP, ICAM-1, and PD-L1 on myeloblasts (see Supplemental Figure 1 for gating strategies) after culture for 48 hours without or with IFN-γ (1 ng/mL or 50 ng/mL). FMO (fluorescence minus 1) samples were cultured with 50 ng/mL IFN-γ. (**C**) BM samples from participants were collected between 2 and 14 days prior to and 48–60 hours after in vivo IFN-γ. Shown is expression of HLA-ABC, HLA-DR, HLA-DP, ICAM-1, and PD-L1 on myeloblasts. p-STAT1 and HLA-DR expression of an array of primary AML samples (*n* = 60) before and after in vitro IFN-γ stimulation (**D** and **E**). Shown is fold-induction with IFN-γ (*y* axis) for p-STAT1 (**D**) and HLA-DR (**E**) grouped by ELN classification (*x* axis). (**F**) Representative flow cytometry plots of p-STAT1 and IFN-γ–inducible molecules after in vitro culture with or without IFN-γ. Shown are data (from top to bottom) of samples that manifest IFN-γ induction of p-STAT1 and HLA-DR, only p-STAT1, or neither.

We tested in vitro responsiveness of myeloblasts and other BM-resident hematopoietic cells to IFN-γ using BM samples collected prior to in vivo IFN-γ. We also compared the phenotypes of BM cells before and after in vivo treatment with IFN-γ. Using specimens collected prior to IFN-γ therapy, we assessed IFN-γ responsiveness at the level of the IFN-gR by measuring phosphorylated STAT1 (p-STAT1) at 15 minutes after in vitro IFN-γ stimulation (gating strategy, Supplemental Figure 1). As an internal positive control for the assay, we analyzed p-STAT1 induction in cells other than myeloblasts. IFN-γ induced p-STAT1 in CD13^+^CD11b^–^ cells, CD11b^+^CD13^–^ cells, and B cells whereas neither CD4^+^ nor CD8^+^ cells were responsive (Supplemental Figure 1). CD34^+^ cells and other immature myeloid cells (CD13^+^CD11b^–^ or CD11b^+^CD13^–^) from all patients phosphorylated STAT1, indicative of a functional IFN-gR (Figure 3A). To assay for IFN-γ–induced phenotypic changes, we also analyzed myeloblast surface expression of HLA-DR, HLA-ABC, HLA-DP, ICAM-1, and programmed cell death ligand 1 (PD-L1) 48 hours after in vitro IFN-γ stimulation (Figure 3B). Baseline HLA-DR expression varied across samples, as has been previously reported (47). All evaluable samples, except for those from patient 7, exhibited at least 1 clear subpopulation of HLA-DR^+^ cells before IFN-γ. After IFN-γ stimulation, HLA-DR upregulation was heterogeneous across the samples, with the largest changes in the samples from patient 7 (whose blasts were mostly HLA-DR^–^ at baseline) and patient 5. ICAM-1 was upregulated in all evaluable samples except for that from patient 4. HLA-DP changes were subtle, with modest increases in MFI, again with the exception of patient 4. HLA-ABC expression was unchanged, other than in the sample from patient 7. PD-L1 expression was also heterogeneous across and within specimens, with only the CD34^+^ cells from patient 5 convincingly upregulating PD-L1 with IFN-γ. Taken together, these data show that despite all samples demonstrating clear induction of p-STAT1, downstream changes were heterogeneous, even in the products of genes known to be induced by IFN-γ in other cell types.

We also examined the surface expression of HLA-DR, HLA-ABC, HLA-DP, ICAM-1, and PD-L1 on BM cells collected before and 48–60 hours after the first or second in vivo dose of IFN-γ (Figure 3C). A bead-based approach was used to standardize HLA-DR expression in the pre–and post–IFN-γ specimens (see Methods). We first looked for potential IFN-γ–induced changes in nonleukemic cells. All samples exhibited at least 1 change in a cell population consistent with an IFN-γ effect in the post–IFN-γ sample relative to the pre–IFN-γ sample (Supplemental Figure 2). Myeloblasts from all participants except for those from patients 5 and 7 showed at least 1 phenotypic change in the pre– and post–IFN-γ samples, though these were heterogeneous. Samples from patients 1, 4, and 6 manifested a loss of an HLA-DR^dull^ population without a change in the main population whereas there was a clear positive shift in the entire population in patient 3. There was no significant change in HLA-DR in samples from patient 2 and even patient 7, whose sample had a very strong in vitro response. There was no convincing upregulation of HLA-ABC except an apparent loss of a low-staining population in patient 4. Interestingly, PD-L1 expression was generally unchanged or downregulated after IFN-γ treatment except for patient 3.

We also compared the in vitro and in vivo IFN-γ responsiveness of myeloblasts. Those from patients 1 and 4 showed a similar loss of HLA-DR^dull^ cells in response to IFN-γ in vitro and in vivo. In contrast, myeloblasts from patient 4 lost the HLA-DR^dull^ population in vitro but not in vivo, and myeloblasts from patient 7 strongly upregulated HLA-DR in vitro but did not do so after in vivo treatment, which may suggest a suboptimal in vivo exposure of myeloblasts to IFN-γ.

To investigate in vitro IFN-γ responsiveness of AML more broadly, we studied a set of AML samples from 60 patients newly diagnosed with AML spanning a spectrum of cytogenetic and molecular abnormalities as categorized by the European LeukemiaNet 2017 (ELN 2017) AML risk classification (48). To do so, we analyzed p-STAT1 and expression of HLA-DR, HLA-DP, HLA-ABC, ICAM-1, and PD-L1 before and after IFN-γ treatment in vitro. Most samples induced p-STAT1 (51/60, 85%; Figure 3D aggregate data; Figure 3F, representative staining), defined by ≥1.5-fold changes in the standardized p-STAT1 MFI, as previously reported (49). However, the magnitudes of the change in p-STAT1 were variable. IFN-γ responsiveness was also heterogeneous as measured by upregulation of HLA-DR defined by ≥1.5-fold changes in MFI (Figure 3E). Neither induction of p-STAT1 nor upregulation of HLA-DR correlated with ELN 2017 AML risk categories. IFN-γ both induced p-STAT1 and upregulated HLA-DR in 38 samples (Figure 3F, representative positive response). However, 13 AML samples induced p-STAT1 without a change in HLA-DR (Figure 3F). In a small minority of samples (*n* = 3), AML phenotype cells neither phosphorylated STAT1 nor upregulated HLA-DR (Figure 3F), though other lineage cells in the sample phosphorylated STAT1 (Supplemental Figure 3).

### Cytokine profiling.

**Figure 4:**
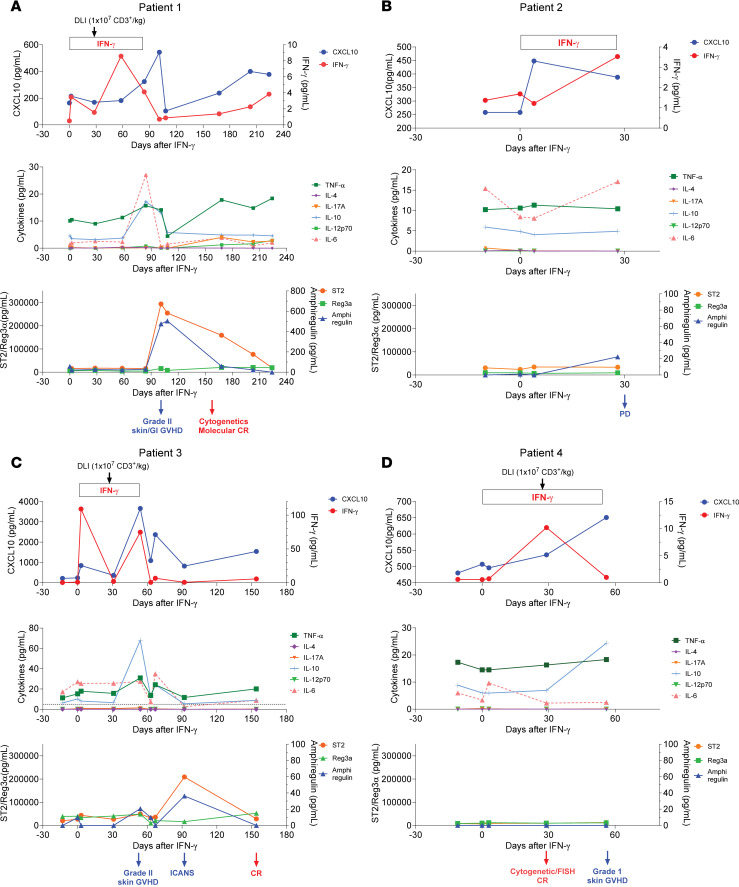
Serial measurements of CXCL10 (IP-10), IFN-γ, other cytokines, and the GVHD biomarkers ST2, Reg3α, and amphiregulin relative to clinical events. (**A**–**D**) Shown are data from patients 1–4, respectively.

Plasma samples from the 4 patients in cohort 1 were available for analysis of cytokine concentrations. Plasma was collected before IFN-γ, 48–72 hours after IFN-γ, 4 weeks after IFN-γ monotherapy before DLI, 4 weeks after DLI, and at subsequent times associated with clinical events (GVHD, CR, or disease progression). Figure 4, A–D, depict the levels of CXCL10, IFN-γ, other cytokines (TNF-α, IL-4, IL-17A, IL-10, IL12p70, and IL-6), and GVHD biomarkers (ST2, Reg3α, amphiregulin) during the study period. Patients 1–3 had an increase in IFN-γ, CXCL10 (which can be induced by IFN-γ), or both, comparing the pre–IFN-γ and first post–IFN-γ samples. CXCL10 had a second peak in patients 1 and 3, coincident with the development of GVHD. Patient 1 had a rise in both amphiregulin and ST2 at the time of GVHD diagnosis, whereas their rise in patient 3 was more temporally related to the development of ICANS. Collectively, these inflammatory markers declined with corticosteroid treatment. During the 4 weeks of IFN-γ monotherapy, GVHD biomarkers did not rise, suggesting that IFN-γ alone did not trigger a GVHD response, concordant with the absence of clinical GVHD before DLI.

### scRNA-Seq.

**Figure 5:**
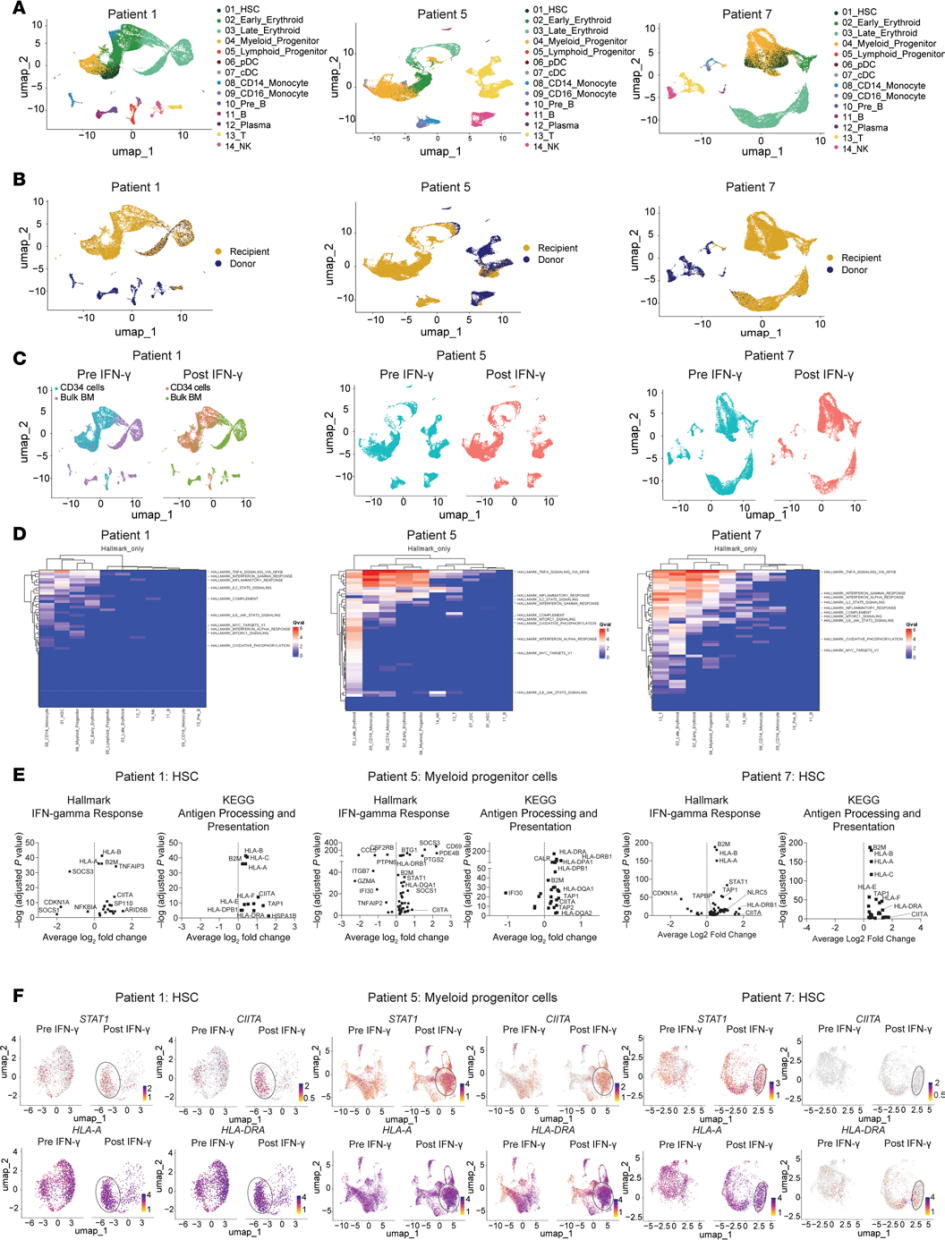
scRNA-Seq of BM samples collected before and after in vivo treatment with IFN-γ. (**A**) Two-dimensional uniform manifold approximation and projection (UMAP) with cell lineage annotations for the combined pre–IFN-γ and post–IFN-γ samples. For patient 1, transcriptomes from the unfractionated and CD34-selected samples are also combined. (**B**) Donor and recipient origin of cells by SNP differences between donor and recipient in transcribed genes mapped onto the same UMAP as in **A**. Most early myeloid and erythroid cells were recipient in origin (consistent with relapsed MDS) whereas lymphoid cells and maturing monocytes were dominantly donor derived. (**C**) Pre– and post–IFN-γ samples are separately annotated. The overall distributions of major lineage clusters of the pre– and post–IFN-γ samples were similar. (**D**) SCPA. Shown are *q* values for changes in the listed pathways (higher values = greater changes in the pathway activity in the post–IFN-γ treatment sample). (**E**) Volcano plots of the expression of selected genes included in key gene sets in **D** for HSCs (patients 1 and 7) and myeloid progenitors (patient 5). Shown are Hallmark sets for “IFN-γ response” and Kyoto Encyclopedia of Genes and Genomes (KEGG) for genes involved in antigen processing and presentation. *X* axis shows average log_2_ fold-change in the post–IFN-γ treatment samples. *Y* axis shows –log_10_ of adjusted *P* values. (**F**) The dominant malignant lineage clusters shown in **A** were separately re-embedded. HSC-like cells were re-embedded for patients 1 and 7, and myeloid progenitor cells were re-embedded for patient 5. The cells in the clustering are depicted as UMAPs with the pre– and post–IFN-γ samples in separate plots. The representative expression levels of *STAT1*, *HLA-A*, *CIITA*, and *HLA-DRA* are shown in each patient. Ovals surround regions of differences in the re-embedded pre– and post–IFN-γ transcriptomes.

We performed single-cell RNA-Seq (scRNA-Seq) on BM samples collected before IFN-γ treatment and 48–60 hours after the first or second dose of IFN-γ from 3 patients (patients 1, 5, and 7). We first analyzed BM samples using unsupervised cluster analysis. Using a healthy human single-cell expression atlas (50), we annotated cells to lymphoid (T cells, B cells, NK cells, plasma cells) and myeloid lineages, including hematopoietic stem cells (HSCs), early myeloid progenitors, and early or late erythroid cells (Figure 5A), as previously described (51). We successfully distinguished recipient- from donor-derived cells based on single nucleotide polymorphisms (SNPs) in expressed genes (Figure 5B). As expected, most immature pluripotent and myeloid lineage cells (HSCs, myeloid progenitor cells, early erythroid cells) at relapse were of recipient genotypic origin, while lymphoid progenitors, B cells, NK cells, and plasma cells were mostly donor derived. Mature T cells were a mix of donor and recipient cells, consistent with the mixed donor T cell chimerism estimated by clinical assays for each patient (Table 1).

Using scRNA-Seq, we could infer genomic copy number in single cells (52), which correlated with clinical data obtained from patient diagnostic specimens. For example, patient 1 showed that a majority of HSCs, myeloid progenitors, and early erythroid cells harbored der[1;7], add[10], del[20], his disease-defining chromosome alterations (Supplemental Figure 4A). Similarly, in patients 5 and 7, their known recurrent cytogenetics abnormalities were found in the majority of HSCs, myeloid progenitors, and early erythroid cells (Supplemental Figure 4, B and C).

A main goal of our scRNA-Seq analysis was to find evidence of in vivo IFN-γ activity. Overall, the hematopoietic cell lineage distributions of the single-cell transcriptomes of the pre–IFN-γ and post–IFN-γ samples were similar (Figure 5C). To more specifically investigate the effects of in vivo IFN-γ within the major lineage clusters, we performed single-cell pathway analysis (SCPA) (53) categorized by the Hallmark Molecular Signatures Database (54), comparing the pre– and post–IFN-γ samples (Figure 5D). Changes in transcripts in the post–IFN-γ specimens were predominantly found in the nonlymphoid subsets (HSC-like cells, myeloid progenitors, and CD14^+^ or CD16^+^ monocytes and erythroid cells), consistent with the lack of STAT1 phosphorylation of lymphoid cells when cultured with IFN-γ in vitro (Figure 3A and Supplemental Figure 1). Reassuringly, the IFN-γ response pathway was activated in HSC-like cells (patient 1 and 7) and myeloid progenitor cells (patients 1, 5, 7) in the post–IFN-γ samples. Post–IFN-γ samples also had evidence of activation of “TNF-α signaling via NFκb” in HSC-like and myeloid progenitor cell populations. “IL2 STAT5 signaling” and “inflammatory response” pathways were also engaged in post–IFN-γ myeloid populations.

To further analyze the impact of IFN-γ effect on malignant cells, we plotted the differentially expressed genes within the Hallmark IFN-γ response category in Figure 5D, focusing on the dominant malignant myeloid cell subclusters for each patient: the HSC-like population for patients 1 and 7 and myeloid progenitor population for patient 5 (Figure 5E). Importantly, there were significant changes in genes known to be induced by IFN-γ. IFN-gR signaling is also known to induce changes in genes involved in antigen presentation. We therefore also analyzed the expression of specific genes in the KEGG antigen presentation pathway (55) in the same cell subsets, which also revealed changes consistent with in vivo IFN-γ activity (Figure 5E).

Finally, to better focus on IFN-γ–induced changes in the malignant lineage clusters for each patient, HSC-like cells were re-embedded for patients 1 and 7 whereas myeloid progenitor cells were reclustered for patient 5 (Figure 5F). There were global changes in gene expression after IFN-γ treatment evident in the single-cell profiles (see regions bound by ovals). The expression levels of selected IFN-γ–inducible genes (*STAT1*, *HLA-A*, *CIITA*, and *HLA-DRA*) also showed distinct patterning after treatment. In patient 1, cells expressing *CIITA*, *HLA-A*, and *HLA-DRA* were enriched in the bottom left cluster of the plot. In patient 5 post–IFN-γ cells with similar expression of IFN-γ–inducible genes globally altered their transcriptomic profiles after therapy. In patient 7, changes in expression were more subtle with the appearance of a small new cluster in the lower right.

## Discussion

Relapsed disease is the single most common cause of treatment failure in patients who undergo an alloSCT for treatment of AML or MDS. Unfortunately, current salvage approaches yield durable remissions only in a small minority of patients. Data from mouse models and from AML specimens collected before and after transplant suggested that IFN-γ could be important to sensitize myeloblasts to T cell killing (32, 38, 39) and that alloreactive T cells develop exhaustion, manifested by decreased IFN-γ production (40). This led us to perform a phase I clinical trial of IFN-γ combined with a DLI as a source of unexhausted T cells in patients with AML or MDS that relapsed after alloSCT.

IFN-γ monotherapy was safe and well tolerated; no participants developed GVHD during this period. The combination of IFN-γ and DLI was, however, associated with steroid-sensitive GVHD in the 4 participants who achieved CRs. We also observed ICANS in 2 participants and IPS in 1 participant, all of which were steroid responsive, consistent with similar immune-mediated syndromes commonly observed after chimeric antigen receptor (CAR) T cell therapy. To our knowledge, ICANS is uncommon after DLI, though systematic reports on this are lacking. High serum levels of IFN-γ are induced by CAR T cells (56) and were reported to correlate with ICANS (57), which supports the possibility that IFN-γ, combined with expanding T cells, was contributory to ICANS in our study.

Efficacy was a secondary outcome, and it is important not to overinterpret results given the small sample size and patient heterogeneity. Nevertheless, an MRD^neg^ CR rate of 67% (4/6) in patients who received both IFN-γ and DLI is promising. Importantly, these responses were durable, with a 1-year survival rate of 83% and a median overall survival (OS) of 24 months (range, 3–32 months) in responders and 9.4 months (range 0.5–32 months) for the entire cohort. All responders achieved full reconstitution of donor myeloid and lymphoid hematopoiesis, despite a wide range of pretreatment chimerism. Interestingly, 1 patient with mostly donor CD3 chimerism before treatment achieved an MRD^neg^ CR with IFN-γ monotherapy prior to receiving a DLI, suggesting that IFN-γ alone could be a rescue strategy in selected patients with low-level relapse and a high degree of donor T cell chimerism.

Notably, we observed CRs in patients with features that predict for poor outcomes with traditional therapies. Patient 3 had *TP53*-mutated AML with complex cytogenetics and developed an MRD-positive relapse 33 days posttransplant. Despite salvage treatment with a hypomethylating agent and venetoclax followed by enasidenib, this patient progressed to overt relapse and progressively decreasing donor chimerism. He achieved a durable MRD^neg^ CR 154 days after starting IFN-γ and 121 days after the DLI. Patient 6, who had complex cytogenetics with *NRAS* and *KRAS* mutations, also achieved a CR. However, patient 7 had rapidly progressive disease such that there would not have been time for an alloimmune response to develop, suggesting that such patients would not be appropriate for the IFN-γ/DLI approach.

Consistent with prior work, in vitro IFN-γ induced p-STAT1 in myeloblasts from all evaluable patients and in the majority of archived specimens (49). In contrast with the relatively consistent p-STAT1 induction, upregulation of surface proteins known to be potentially IFN-γ responsive was heterogeneous across specimens, indicative of more complexity in the regulation of these genes. There were in vivo effects of IFN-γ on BM-resident cells in all patients, but as was the case in vitro, these were again heterogeneous. For example, there was a grossly similar loss of HLA-DR^dull^ myeloblasts in vitro and in vivo in patients 1 and 4. However, samples from patients 5 and 7 had dramatic HLA-DR upregulation in vitro but not in vivo. Nonetheless, despite the lack of phenotype changes in the samples from patients 5 and 7, scRNA-Seq supported at least some level of IFN-γ action on the dominant malignant myeloid population.

Taken together, the flow cytometry, scRNA-Seq, and plasma chemokine data indicate that levels of IFN-γ sufficient for action on BM cells were generally achieved. However, our data suggest that doses higher than that established to treat chronic granulomatous disease (CGD) might have a greater impact on myeloblasts. This could be tested in future clinical studies using cohort-based or intrapatient dose escalation. Responses could be monitored by plasma sampling, analysis of BM cells by flow cytometry, or the quantitation of selected transcripts in sorted cell subsets. A larger study could also explore whether qualities of in vitro or in vivo IFN-γ–induced changes correlate with clinical responses, which if proved to be true, could aid in participant selection in future clinical trials.

Mouse data support a leukemia cell–intrinsic mechanism of IFN-γ action. However, it is notable that hematologic responses, including CRs, extended beyond the cessation of IFN-γ treatment coincident with the development of GVHD. It is possible that during the initiation of the alloimmune response, IFN-γ was most important to sensitize myeloblasts. However, IFN-γ may not be as necessary when there is a larger magnitude anti–minor histocompatibility antigen (anti-miHA) response. The GVHD response itself may also generate IFN-γ (see CXCL10 levels in patients 1 and 3). More effective early killing by the initial wave of alloreactive T cells potentiated by IFN-γ could enhance crosspresentation of key miHAs (40), further amplifying an effective T cell response. IFN-γ is also known to induce the immunoproteasome, which could enhance the presentation of targeted miHAs.

In conclusion, in this first-in-human trial, IFN-γ and DLI was a safe and well-tolerated treatment for myeloid malignancies that had relapsed after alloSCT. Although GVHD was observed in all responders, it was universally steroid sensitive. Although efficacy was not the primary objective of this phase I trial, CR and survival data suggest the potential of this treatment approach, which will need to be tested in a larger clinical trial.

## Methods

### Sex as a biological variable

Our study eligibility criteria permitted both male and female participants, though only men were enrolled. Both AML and MDS are more common in men.

### Clinical study design

#### Overview.

This was a phase I, single-center, open-label study (see Supplemental Data 1). There were 2 stages: an IFN-γ monotherapy stage and a combination stage in which patients received both IFN-γ and DLI. In the monotherapy phase, IFN-γ (Actimmune, Horizon Therapeutics) was self-administered by patients at 100 µg 3 times a week for 4 weeks. Due to the tolerability of IFN-γ monotherapy in the first 4 patients, we amended the protocol to enroll a second cohort with a shortened monotherapy phase of 1 week using the same 100 µg 3 times a week regimen in order to reduce the potential risk of disease progression while awaiting the combination treatment. In the combination phase, both cohorts received DLI at a dose of 1 × 10^7^ CD3 cells/kg and continued IFN-γ for 8 additional weeks, with a taper to 100 µg once per week for the last 4 weeks. Patients with less than a complete response and without GVHD were eligible for a second DLI at a recommended dose of 5 × 10^7^ CD3 cells/kg beginning 4 weeks after the first DLI.

The dose of IFN-γ (100 µg fixed dose) was chosen to parallel the FDA-approved dose of 50 µg/m^2^ for CGD. The inclusion criteria for this trial stipulated that participants have a body surface area between 1.5 m^2^ and 2.5 m^2^, which would yield Actimmune doses between 75% and 125% of the approved per square meter dose.

#### Eligibility criteria.

Patients 18 years of age or older with an Eastern Cooperative Oncology Group performance status score of 0 to 2 and relapsed AML or MDS after HLA-matched alloSCT were eligible. Relapse was defined as having abnormal myeloblasts comprising 0.5% to 30% of total cells as determined by flow cytometry for cohort 1 and up to any level for cohort 2. Prior therapies for relapse were allowed, with washout periods prior to start of IFN-γ as follows: ≥1 week for hypomethylating agents, venetoclax, and targeted therapies (tyrosine kinase inhibitors, FMS-like tyrosine kinase 3 inhibitors, isocitrate dehydrogenase 1/isocitrate dehydrogenase 2 inhibitors) and ≥4 weeks for cytotoxic chemotherapies. Patients were excluded if they had primary engraftment failure, grades 3 or 4 acute GVHD (aGVHD) at time of enrollment or history of grade 4 aGVHD per Mount Sinai Acute GVHD International Consortium (MAGIC) Criteria, moderate or severe chronic GVHD (cGVHD) per National Institutes of Health Consensus Criteria at time of enrollment, initiation of new systemic immunosuppressive medications to treat GVHD in the 2 weeks prior to enrollment, or systemic corticosteroids at a dose of ≥0.5 mg/kg/d of prednisone or its equivalent.

#### Outcome assessments.

The primary objective was to assess the safety of IFN-γ as monotherapy and in combination with DLI. Secondary objectives were to evaluate clinical efficacy of the combination IFN-γ and DLI regimen and to describe the in vivo effects of IFN-γ on myeloblast HLA expression and gene expression.

Dose-limiting toxicity was defined as death from any cause attributable to IFN-γ within 30 days of the first IFN-γ dose. Adverse events that emerged during treatment were defined as events that began or worsened from the time of the first dose of IFN-γ to 28 days after the last dose and were graded according to the National Cancer Institute Common Terminology Criteria for Adverse Events, version 5.0. Serious adverse events were those that resulted in death, were life-threatening, led to inpatient hospitalization or prolongation of hospitalization, or caused persistent or significant incapacity. GVHD was of particular interest given the known risk of GVHD with DLI and the possibility for IFN-γ monotherapy to precipitate or worsen GVHD. aGVHD was assessed during treatment and for 28 days after the last dose according to the MAGIC Criteria, and cGVHD was assessed over the same time frame according to the National Institutes of Health Consensus Criteria.

Clinical efficacy of the combination regimen was assessed by the investigators using the 2017 European LeukemiaNet response criteria, including CR, CR with incomplete hematologic recovery, remission duration, and OS. Clinical responses were evaluated at 4, 12, and 24 weeks after starting IFN-γ by enumerating myeloblasts in BM and peripheral blood; long-term follow-up was also completed by electronic health record review at 52 weeks and at the date of data cutoff of December 31, 2023. MRD was evaluated by flow cytometry on all BM biopsies.

### Sample collection and processing

Research BM aspirates were collected before and 48–72 hours after the first or second dose of IFN-γ. Peripheral blood was collected before the first dose of IFN-γ; 48–72 hours, 4 weeks, and 12 weeks after the first dose of IFN-γ; and at the onset of GVHD and at select post-GVHD time points. Plasma was isolated from peripheral blood by centrifuging 2–3 mL whole blood at 376*g* for 10 minutes twice and was stored at –80°C. BM mononuclear cells (BMCs) or peripheral blood mononuclear cells (PBMCs) were isolated by Ficoll-Hypaque density gradient centrifugation using SepMate (StemCell Technologies). Aliquots of BMCs and PBMCs were immediately analyzed by flow cytometry as described below. The remaining cells were cryopreserved in liquid nitrogen until further use. Deidentified primary AML samples were obtained from Pitt Biospecimen Core.

### p-STAT1 assay

Freshly collected BMCs or PBMCs were plated at 10^6^ cells per well in a 96-well plate in complete media consisting of RPMI (catalog R8758 MilliporeSigma) supplemented with 10% human serum (catalog H3667, MilliporeSigma) and penicillin-streptomycin (catalog 15140122, Thermo Fisher Scientific). After washing with PBS, the cells were stained with fixable violet live-dead dye (Vivid; Invitrogen, Thermo Fisher Scientific) and monoclonal antibodies against CD34, CD117, CD45, CD38, CD13, CD11b, CD14, HLA-DR, CD3, CD4, CD8, and CD19. The list of monoclonal antibodies is summarized in Supplemental Table 2. The cells were then washed with PBS and resuspended in complete media with IFN-γ at 0, 1, or 50 ng/mL. Cells were cultured at 37°C 5% CO_2_ for 15 minutes followed by adding prewarmed PhosFlow Cytofix buffer (catalog 557870 BD Biosciences) for an additional 10 minutes. Cells were permeabilized with PhosFlow Perm Buffer III (catalog 558050, BD Biosciences) at 4°C for 30 minutes. After washing, anti–p-STAT1 antibody was added, and cells were incubated at room temperature for 30 minutes. The cells were washed and resuspended in 0.5% bovine serum albumin PBS buffer (FCS buffer) and acquired on a Cytek Biosciences Aurora.

### Analyzing surface molecule changes in response to IFN-γ

Thawed BMCs or PBMCs were plated at 10^6^ cells per well in a 96-well plate in complete medium with 0, 1, or 50 ng/mL IFN-γ (PeproTech) and were cultured for 48 hours. After washing with PBS, the cells were stained with fixable violet live-dead dye for 15 minutes followed by staining with monoclonal antibodies against CD34, CD117, CD45, CD38, CD13, CD11b, CD14, HLA-ABC, HLA-DR, ICAM-1, and PD-L1 for 15 minutes (Supplemental Table 2). The cells were acquired on an Aurora using standardization beads (Quantum MESF, Bangs Laboratories), to enable the quantification of changes in HLA-DR expression.

### Cytokine measurements

Plasma cytokines were measured using a microfluidic channel system (Simple Plex, Ella, Bio-Techne) according to manufacturer’s instruction. We used one cartridge to measure cytokines (IFN-γ, CXCL10/IP-10, IL-10, IL-12 p70, IL17A, IL-4, IL-6, TNF-α; catalog SPCKE-PS003033) and a second to measure GVHD biomarkers (ST2, Reg3α, amphiregulin; catalog SPCKC-PS006688). Cytokines were measured in duplicate and GVHD biomarkers in triplicate. Concentrations were calculated from the mean signal using analyte- and lot-specific factory standard curves created on lot-to-lot basis.

### scRNA-Seq

scRNA-Seq was performed on BM from patients 1, 5, and 7 collected before and 48–72 hours after the first or second dose of IFN-γ. For patient 1, we prepared bulk, unenriched BM as well as BM enriched in CD34^+^ BM myeloblasts by immunomagnetic selection; for patients 5 and 7, only bulk BM cells were analyzed. Samples were captured with Chromium Controller (10x Genomics), and libraries were generated using the Chromium Next GEM Single Cell 5′ v2 (Dual Index) kit (10x Genomics) according to the manufacturer’s instructions. Libraries were sequenced using a NextSeq 2000 in 1 run (Illumina P3 kit), with a target of 20,000 reads per cell for gene expression. Illumina BCL files were then demultiplexed and processed using Cell Ranger (10x Genomics). Cells meeting the following criteria (calculated using Seurat) (58) were retained for downstream analysis: unique molecular indices per cell greater than 700 and less than 50,000 and percentage mitochondrial RNA less than 12%. Dimensionality reduction was performed using Seurat with standard parameters using the top 10,000 variable features. We used unsupervised cluster analysis and referred to a healthy human single-cell expression atlas to annotate BM cells to lymphoid (lymphoid progenitors, T cells, B cells, NK cells, and plasma cells) and myeloid lineages, HSCs at different maturation stages, early myeloid progenitors, and early or late erythroid cells. Malignant leukemia blasts were identified by their dysregulated transcriptome, referring to a single-cell expression atlas from healthy human BM that allows for detection of residual leukemia at a minimum level of 0.05% (59), and malignant clones were identified by annotating recurrent chromosome alterations known to each patient. We distinguished recipient origin cells from donor-derived hematopoietic cells based on SNP differences in expressed genes (60). InferCNV (61) was used to identify the somatic copy number alterations in the chromosome in single cells using the T cell population as a set of reference “normal” cells. SCPA was used to identify the changes in the pathway activity comparing BM cells collected before and after IFN-γ (53).

### Statistics

Patients were included in analyses of biological efficacy if they provided both pre– and post–IFN-γ BM samples. Descriptive statistics were used to summarize clinical and laboratory variables. Median survival was estimated using Kaplan-Meier methods. *P* < 0.05 was considered statistically significant.

### Study approval

The study was approved by University of Pittsburgh Institutional Review Board (STUDY20060133: HCC 20-092). Written informed consent was obtained from all participants enrolled in this study.

### Data availability

All raw data used in this manuscript are available in the Supporting Data Values file. scRNA-Seq data (accession number GSE291800) were deposited at Gene Expression Omnibus, through the National Center for Biotechnology Information, National Library of Medicine, and are publicly available.

## Author contributions

SI designed the clinical trial and laboratory research, conducted and supervised clinical trial activities, conducted laboratory research, collected and interpreted data, and wrote the manuscript. EG conducted the clinical trial, collected data, and wrote the manuscript. KV, BN, SB, EMM, and SF conducted laboratory research, collected and interpreted data, and reviewed the manuscript. WDS conceived the project, designed the clinical trial and laboratory research, interpreted data, and wrote the manuscript.

## Supplementary Material

Supplemental data

Supplemental data 1

ICMJE disclosure forms

Supporting data values

## Figures and Tables

**Table 1:**
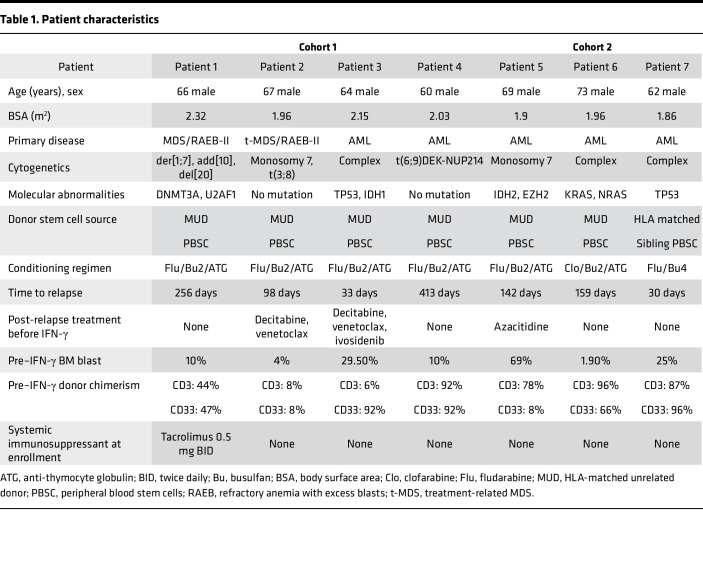
Patient characteristics

**Table 2:**
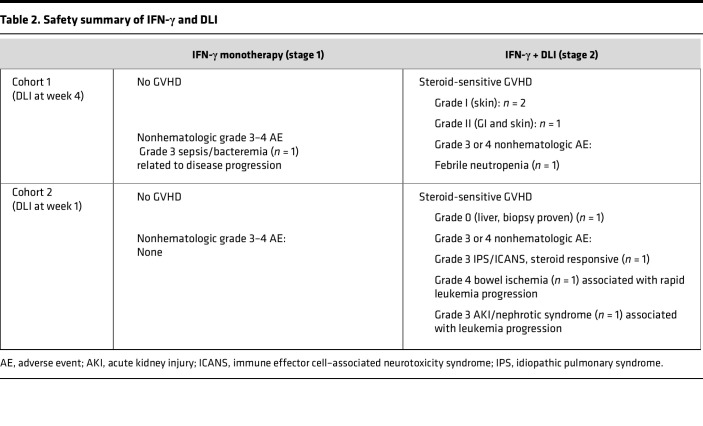
Safety summary of IFN-γ and DLI

## Version 1 03/25/2025

In-Press Preview

## Version 2 05/08/2025

Electronic publication

## References

[1] Bleakley M, Riddell SR (2004). Molecules and mechanisms of the graft-versus-leukaemia effect. Nat Rev Cancer.

[2] Alyea EP (2010). NCI First International Workshop on The Biology, Prevention and Treatment of Relapse after Allogeneic Hematopoietic Cell Transplantation: report from the committee on prevention of relapse following allogeneic cell transplantation for hematologic malignancies. Biol Blood Marrow Transplant.

[3] Bishop MR (2010). Introduction to the reports from the National Cancer Institute First International Workshop on the Biology, Prevention, and Treatment of Relapse after Allogeneic Hematopoietic Stem Cell Transplantation. Biol Blood Marrow Transplant.

[4] Wayne AS (2013). Proceedings from the National Cancer Institute’s Second International Workshop on the Biology, Prevention, and Treatment of Relapse after Hematopoietic Stem Cell Transplantation: introduction. Biol Blood Marrow Transplant.

[5] Horowitz M (2018). Epidemiology and biology of relapse after stem cell transplantation. Bone Marrow Transplant.

[6] Kharfan-Dabaja MA (2018). Association of second allogeneic hematopoietic cell transplant vs donor lymphocyte infusion with overall survival in patients with acute myeloid leukemia relapse. JAMA Oncol.

[7] Schmid C (2012). Treatment, risk factors, and outcome of adults with relapsed AML after reduced intensity conditioning for allogeneic stem cell transplantation. Blood.

[8] Rautenberg C (2020). Prediction of response and survival following treatment with azacitidine for relapse of acute myeloid leukemia and myelodysplastic syndromes after allogeneic hematopoietic stem cell transplantation. Cancers (Basel).

[9] Claiborne J (2019). Managing post allograft relapse of myeloid neoplasms: azacitidine and donor lymphocyte infusions as salvage therapy. Leuk Lymphoma.

[10] Sommer S (2018). Decitabine in combination with donor lymphocyte infusions can induce remissions in relapsed myeloid malignancies with higher leukemic burden after allogeneic hematopoietic cell transplantation. Leuk Res.

[11] Schroeder T (2018). Treatment of relapsed AML and MDS after allogeneic stem cell transplantation with decitabine and DLI-a retrospective multicenter analysis on behalf of the German Cooperative Transplant Study Group. Ann Hematol.

[12] Craddock C (2016). Tolerability and clinical activity of post-transplantation azacitidine in patients allografted for acute myeloid leukemia treated on the RICAZA Trial. Biol Blood Marrow Transplant.

[13] Schroeder T (2015). Treatment of acute myeloid leukemia or myelodysplastic syndrome relapse after allogeneic stem cell transplantation with azacitidine and donor lymphocyte infusions--a retrospective multicenter analysis from the German Cooperative Transplant Study Group. Biol Blood Marrow Transplant.

[14] Steinmann J (2015). 5-Azacytidine and DLI can induce long-term remissions in AML patients relapsed after allograft. Bone Marrow Transplant.

[15] Schroeder T (2013). Azacitidine and donor lymphocyte infusions as first salvage therapy for relapse of AML or MDS after allogeneic stem cell transplantation. Leukemia.

[16] Krakow EF (2022). Intensive chemotherapy for acute myeloid leukemia relapse after allogeneic hematopoietic cell transplantation. Am J Hematol.

[17] Joshi M (2021). Salvage use of venetoclax-based therapy for relapsed AML post allogeneic hematopoietic cell transplantation. Blood Cancer J.

[18] Amit O (2021). Venetoclax and donor lymphocyte infusion for early relapsed acute myeloid leukemia after allogeneic hematopoietic cell transplantation. A retrospective multicenter trial. Ann Hematol.

[19] Schuler E (2021). Treatment of myeloid malignancies relapsing after allogeneic hematopoietic stem cell transplantation with venetoclax and hypomethylating agents-a retrospective multicenter analysis on behalf of the German Cooperative Transplant Study Group. Ann Hematol.

[20] Aldoss I (2018). Efficacy of the combination of venetoclax and hypomethylating agents in relapsed/refractory acute myeloid leukemia. Haematologica.

[21] Zucenka A (2021). Venetoclax-based salvage therapy followed by Venetoclax and DLI maintenance vs FLAG-Ida for relapsed or refractory acute myeloid leukemia after allogeneic stem cell transplantation. Bone Marrow Transplant.

[22] Zuanelli Brambilla C (2021). Relapse after allogeneic stem cell transplantation of acute myelogenous leukemia and myelodysplastic syndrome and the importance of second cellular therapy. Transplant Cell Ther.

[23] Zhao P (2022). Venetoclax plus azacitidine and donor lymphocyte infusion in treating acute myeloid leukemia patients who relapse after allogeneic hematopoietic stem cell transplantation. Ann Hematol.

[24] Mackinnon S (1995). Adoptive immunotherapy evaluating escalating doses of donor leukocytes for relapse of chronic myeloid leukemia after bone marrow transplantation: separation of graft-versus-leukemia responses from graft-versus-host disease. Blood.

[25] (1997). Donor leukocyte infusions in 140 patients with relapsed malignancy after allogeneic bone marrow transplantation. J Clin Oncol.

[26] Horowitz MM (1990). Graft-versus-leukemia reactions after bone marrow transplantation. Blood.

[27] Porter DL (1994). Induction of graft-versus-host disease as immunotherapy for relapsed chronic myeloid leukemia. N Engl J Med.

[28] Schmid C (2007). Donor lymphocyte infusion in the treatment of first hematological relapse after allogeneic stem-cell transplantation in adults with acute myeloid leukemia: a retrospective risk factors analysis and comparison with other strategies by the EBMT Acute Leukemia Working Party. J Clin Oncol.

[29] Luznik L (2022). Randomized phase III BMT CTN trial of calcineurin inhibitor-free chronic graft-versus-host disease interventions in myeloablative hematopoietic cell transplantation for hematologic malignancies. J Clin Oncol.

[30] Matte CC (2004). Graft-versus-leukemia in a retrovirally induced murine CML model: mechanisms of T-cell killing. Blood.

[31] Matte-Martone C (2008). CD8^+^ but not CD4^+^ T cells require cognate interactions with target tissues to mediate GVHD across only minor H antigens, whereas both CD4^+^ and CD8^+^ T cells require direct leukemic contact to mediate GVL. Blood.

[32] Matte-Martone C (2017). Differential requirements for myeloid leukemia IFN-γ conditioning determine graft-versus-leukemia resistance and sensitivity. J Clin Invest.

[33] Elmaagacli AH (2011). Early human cytomegalovirus replication after transplantation is associated with a decreased relapse risk: evidence for a putative virus-versus-leukemia effect in acute myeloid leukemia patients. Blood.

[34] Foley B (2012). Cytomegalovirus reactivation after allogeneic transplantation promotes a lasting increase in educated NKG2C^+^ natural killer cells with potent function. Blood.

[35] Han T (2022). Cytomegalovirus infection is associated with rapid NK differentiation and reduced incidence of relapse in HLA matched sibling donor transplant patients. Clin Exp Immunol.

[36] Turki AT (2022). Impact of CMV reactivation on relapse of acute myeloid leukemia after HCT is dependent on disease stage and ATG. Blood Adv.

[37] Akahoshi Y (2023). CMV reactivation after allogeneic HCT is associated with a reduced risk of relapse in acute lymphoblastic leukemia. Blood Adv.

[38] Christopher MJ (2018). Immune escape of relapsed AML cells after allogeneic transplantation. N Engl J Med.

[39] Toffalori C (2019). Immune signature drives leukemia escape and relapse after hematopoietic cell transplantation. Nat Med.

[40] Zhou M (2020). T cell exhaustion and a failure in antigen presentation drive resistance to the graft-versus-leukemia effect. Nat Commun.

[41] Senjo H (2023). Calcineurin inhibitor inhibits tolerance induction by suppressing terminal exhaustion of donor T cells after allo-HCT. Blood.

[42] Ni X (2017). PD-L1 interacts with CD80 to regulate graft-versus-leukemia activity of donor CD8^+^ T cells. J Clin Invest.

[43] Deng R (2015). B7H1/CD80 interaction augments PD-1-dependent T cell apoptosis and ameliorates graft-versus-host disease. J Immunol.

[44] Asakura S (2010). Alloantigen expression on non-hematopoietic cells reduces graft-versus-leukemia effects in mice. J Clin Invest.

[45] Blazar BR (2003). Blockade of programmed death-1 engagement accelerates graft-versus-host disease lethality by an IFN-gamma-dependent mechanism. J Immunol.

[46] Fujiwara H (2014). Programmed death-1 pathway in host tissues ameliorates Th17/Th1-mediated experimental chronic graft-versus-host disease. J Immunol.

[47] Wetzler M (2003). HLA-DR antigen-negative acute myeloid leukemia. Leukemia.

[48] Dohner H (2022). Diagnosis and management of AML in adults: 2022 recommendations from an international expert panel on behalf of the ELN. Blood.

[49] Irish JM (2004). Single cell profiling of potentiated phospho-protein networks in cancer cells. Cell.

[50] George N (2024). Expression Atlas update: insights from sequencing data at both bulk and single cell level. Nucleic Acids Res.

[51] Krakow EF (2024). HA-1-targeted T-cell receptor T-cell therapy for recurrent leukemia after hematopoietic stem cell transplantation. Blood.

[52] https://github.com/broadinstitute/infercnv/blob/master/inst/CITATION#.

[53] Bibby JA (2022). Systematic single-cell pathway analysis to characterize early T cell activation. Cell Rep.

[54] Liberzon A (2015). The Molecular Signatures Database (MSigDB) hallmark gene set collection. Cell Syst.

[55] Kanehisa M (2024). KEGG: biological systems database as a model of the real world. Nucleic Acids Res.

[56] Turtle CJ (2016). CD19 CAR-T cells of defined CD4^+^:CD8^+^ composition in adult B cell ALL patients. J Clin Invest.

[57] Gust J (2020). Cytokines in CAR T cell-associated neurotoxicity. Front Immunol.

[58] Satija R (2015). Spatial reconstruction of single-cell gene expression data. Nat Biotechnol.

[59] Kanaan SB (2021). Single-cell transcriptomics for residual disease detection in acute myelogenous leukemia post allogeneic hematopoietic cell transplantation. Blood.

[60] Heaton H (2020). Souporcell: robust clustering of single-cell RNA-seq data by genotype without reference genotypes. Nat Methods.

[61] https://github.com/broadinstitute/inferCNV.

