# Supplementary material for: IFN-γ and donor leukocyte infusions for relapsed myeloblastic malignancies after allogeneic hematopoietic stem cell transplantation
Source: JCI Insight. 2025 Mar 25;10(9):e190655. doi: 10.1172/jci.insight.190655 (PMC12128990; doi:10.1172/jci.insight.190655)
Supplement: Supplemental data [file jciinsight-10-190655-s094.pdf]

Supplemental material

Supplemental Table 1. Grades and frequencies of all adverse events.

IFN-gamma monotherapy (Stage 1)

Adverse event term	Any grade (n)	%	Grade 3 or 4 (n)	%	Comment
**Hematologic**					
Anemia	4	57%	3	43%	
Decreased ALC	4	57%	4	57%	
Decreased ANC	3	43%	2	29%	
Decreased platelets	2	29%	2	29%	
Decreased WBC	1	14%	1	14%	
**Non-hematologic**	-	-	-	-	
Bone Pain	1	14%	0	0%	
Fatigue	1	14%	0	0%	
Flu like symptoms	3	43%	0	0%	
Foot pain	1	14%	0	0%	
Headache	1	14%	0	0%	
Hypotension- intermittent	1	14%	0	0%	
Insomnia	1	14%	0	0%	
Left lower leg pain	1	14%	0	0%	
Malaise	1	14%	0	0%	
Myalgia	2	29%	0	0%	
Nausea-intermittent	1	14%	0	0%	
Sepsis/Bacteremia	1	14%	1	14%	Probably associated with AML progression
Sinus tachycardia-intermittent	1	14%	0	0%	

IFN-gamma + donor leukocyte infusion (Stage 2)

Adverse event term	Any grade (n)	%	Grade 3 or 4 (n)	%	Comment
**Hematologic**					
Anemia	5	83%	4	67%	
Decreased ANC	5	83%	4	67%	
Decreased ALC	3	50%	2	33%	
Decreased platelets	4	67%	4	67%	
Decreased WBC	6	100 %	6	100 %	
**Non-hematologic**	-	-	-	-	
Abdominal pain	1	17%	0	0%	
Acute kidney injury (nephrotic syndrome)	1	17%	1	17%	Most likely associated with AML progression
Adult respiratory distress syndrome	1	17%	1	17%	IPS, sensitive to steroid
Anorexia	1	17%	0	0%	
Bacteremia	1	17%	0	0%	
Blood bilirubin increased	1	17%	1	17%	

Chills	2	33%	0	0%	
Confusion	2	33%	0	0%	ICANS, responsive to steroid
CRS	1	17%	0	0%	
Decreased Fibrinogen	1	17%	0	0%	
Diarrhea	3	50%	1	17%	
Dizziness	1	17%	0	0%	
Dry skin	1	17%	0	0%	
Dyspnea	1	17%	0	0%	
Encephalopathy	1	17%	0	0%	ICANS, responsive to steroid
Fatigue	2	33%	0	0%	
Febrile neutropenia- SAE	1	17%	1	17%	
Fever	3	50%	0	0%	
Flu-like symptoms- SAE	1	17%	0	0%	Most likely associated with AML progression
Gastrointestinal other: Jejunal ischemia	1	17%	1	17%	
General Disorders other: Night sweats	1	17%	0	0%	
GI other: rectal bleed	1	17%	0	0%	
GVHD	4	67%	0	0%	
Hypotension	2	33%	0	0%	
Hypoxia	1	17%	0	0%	
Increased Alk Phos	2	33%	0	0%	
Increased ALT	3	50%	1	17%	
Increased AST	3	50%	1	17%	
Increased Creatinine	2	33%	0	0%	
Increased GGT	1	17%	1	17%	
Increased INR	1	17%	0	0%	
Insomnia- intermittent	1	17%	0	0%	
Lethargy	1	17%	0	0%	
Myalgia	1	17%	0	0%	
Nausea	4	67%	0	0%	
Pruritis	3	50%	0	0%	
Rash maculo-papular	3	50%	0	0%	
Respiratory other: Immune mediated lung injury	1	17%	0	0%	IPS, sensitive to steroid
Shoulder pain	1	17%	0	0%	
Vomiting	4	67%	0	0%	
Weight loss	1	17%	0	0%	

Supplemental Table 2. List of monoclonal antibodies

Specificity	Fluorochrome	mAb clone	ISOTYPE	Vendor	Catalog #
CCR7 (CD197)	BV510	G043H7	Mouse IgG2a, kappa	Biolgend	3353232
CD117	PerCPCy5.5	HLDA6	Mouse IgG1, kappa	Biolegend	323416
CD11b	BV711	ICRF44	Mouse, IgG1, kappa	Biolegend	301344
CD127	BV711	A019D5	Mouse, IgG1, kappa	Biolegend	351327
CD13	APC	WM15	Mouse IgG1, kappa	Biolegend	301706
CD14	BV785	M5E2	Mouse, IgG2a, kappa	Biolegend	301839
CD14	PB	M5E2	Mouse IgG2a, kappa	Biolegend	301828
CD19	PB	HIB19	Mouse IgG1, kappa	Biolegend	302232
CD19	BV650	HIB19	Mouse IgG1, kappa	Biolegend	302238
CD25	Alexa Fluor 700	M-A251	Mouse, IgG1 kappa	Biolegend	356118
CD274	PECy7	MIH1	Mouse, IgG1, kappa	BD Pharmigen	558017
CD3	APCCy7	UCHT1	Mouse IgG1, kappa	Biolgend	300426
CD3	PE/Dazzle 594	UCHT1	Mouse IgG1, kappa	Biolegend	300450
CD33	BUV395	WM53	Mouse IgG1, kappa	BD Horizon	568374
CD34	PE	581	Mouse IgG1, kappa	Biolegend	343506
CD38	BUV737	HB7	Mouse, IgG1, kappa	BD Horizon	612824
CD4	Alexa Fluor 700	Sk3	Mouse IgG1, kappa	Biolgend	344622
CD4	BUV395	SK3	Mouse, IgG1, kappa	BD Horizon	563550
CD45	BV510	HI30	Mouse, IgG1, kappa	Biolegend	304036
CD45	BV785	HI30	Mouse, IgG1, kappa	Biolegend	304048
CD45RA	BV650	HI100	Mouse IgG2b, kappa	Biolegend	304136
CD45RO	PECy5	UCHL1	Mouse IgG2a, kappa	Biolegend	304208
CD8	BUV737	SK1	Mouse, IgG1, kappa	BD Horizon	612754
CD8	PECy7	SK1	Mouse IgG1, kappa	Biolegend	344712
FoxP3	FITC	234A/E7	Mouse, IgG1, kappa	eBioscience	11-4777-42
Ki67	PerCPCy5.5	Ki67	Mouse IgG1, kappa	BioLegend	350520
HLA-ABC	Alexa Fluor 700	W6/32	Mouse IgG2a, kappa	Biolegend	311438
HLA-DP	BV650	B7/21	Mouse IgG1, kappa	BD OptiBuild™	750871
HLA-DR	APC-Cy7	L243	Mouse IgG2a, kappa	Biolegend	307618
HLA-DR	FITC	L243	Mouse IgG2a, kappa	Biolegend	307604
ICAM-1	PE/Dazzle 594	HA58	Mouse IgG1, kappa	Biolegend	353118
NRP-1 (CD304)	Alexa Fluor 647	EPR3113	Rabbit IgG	Abcam	ab198323
NRP-1 (CD304)	PE	12C2	Mouse IgG2a, kappa	Biolegend	354504
PD1	PE/Dazzle 594	EH12.2H7	Mouse IgG1, kappa	Biolegend	329940
pSTAT1 (py701)	Alexa Fluor 488	Clone 4a	Mouse IgG2a	BD Phosflow™	612596
κ Isotype Ctrl	Alexa Fluor 700		Mouse IgG2a	Biolegend	400248
κ Isotype Ctrl	APC/Cyanine7		Mouse IgG2a	Biolegend	400230
κ Isotype Ctrl	Brilliant Violet 650		Mouse IgG1	Biolegend	400163
κ Isotype Ctrl	FITC		Mouse IgG1	Biolegend	400110
κ Isotype Ctrl	PE/Dazzle 594		Mouse IgG1,	Biolegend	400176
κ Isotype Ctrl	APC		Mouse IgG2b,	Biolegend	400322

Supplemental Figure Legends

Supplemental Figure 1. Gating strategy for analyses of IFN-γ effects. Shown are representative data from the Patient 1 sample. (A) shows the upstream gating for subpopulations in panel (B).

Supplemental Figure 2. Surface expression changes of potentially IFN-γ-inducible surface proteins in different cell populations before and after in vivo IFN-γ. Gating was as in Supplemental Figure 2. Shown are pre- and post- IFN-γ treatment expression of HLA-DR, HLA-ABC, HLA-DP, ICAM-1 and PD-L1 in cells with the following immunophenotypes: $SSC^{low}CD45^{dim}CD34^{+}$, $CD11b^{-}CD13^{+}$, $CD11b^{+}CD13^{+}$, $CD11b^{+}CD13^{-}$, $CD14^{+}CD33^{+}$, and $CD45^{bright}$.

Supplemental Figure 3. Representative flow cytometry plots of pSTAT1 in other lineage cells after in vitro culture with or without IFN-γ.

Supplemental Figure 4. Identification of malignant cells with scRNAseq analysis based on chromosome aberrations. (A). Copy number variation confirms that the majority of HSCs and myeloid progenitors harbor der (1;7), add (10), del (20), known recurrent chromosomal abnormalities in Patient 1's case of MDS. (**B**) Copy number variation inferred by the scRNAseq confirms that the majority of HSCs and myeloid progenitors harbor a chromosome 7 deletion, a feature of AML in Patient 5. (**C**) Copy number variation inferred by scRNAseq confirms that the majority of HSCs and myeloid progenitors harbor abnormalities in chromosomes 3, 17, 18, 21, and 22, features of Patient 7's AML.

Supplemental Data 2: Summary of ICANS cases

Patient 3

A 64-year-old male with AML with complex cytogenetics and *TP53/IDH1* mutations received a matched unrelated donor (MUD) allogeneic stem cell transplantation (alloSCT) conditioned with fludarabine, busulfan, and ATG. He developed relapse with measurable residual disease (MRD) positivity as early as 33 days after transplantation. His disease was refractory to salvage therapies with decitabine/venetoclax and ivosidenib, evidenced by a bone marrow studies showing a hypocellular marrow with 29.5% myeloblasts with low donor chimerism: 25% in bone marrow; associated with 5% in CD33 cells, 6% in CD3 cells in peripheral blood. He was enrolled in this phase I clinical trial and started IFN-γ on day 215 post-transplant and received DLI (10^7 CD3+ cells/kg) on day +33 after IFN-γ. His pre-DLI bone marrow showed 40.5% myeloblasts with slightly improved donor myeloid chimerism: 39% in bone marrow and 53% in 10% in blood $CD33^+$ and $CD3^+$ cells, respectively. On day +54, he developed biopsy-proven grade 2 skin acute GVHD. IFN-γ was discontinued per the protocol. At the onset of GVHD, his peripheral blood chimerism showed further improvement with donor-derived hematopoiesis, including 98% in CD33 cells, 84% in CD3 cells, and 95% in whole blood, which was accompanied by neutrophil and platelet recovery. His skin GVHD rapidly resolved with systemic corticosteroids and low-dose tacrolimus with a trough goal of 5-10 ng/mL. After he completed a steroid taper, he was admitted for fever and multi-focal pneumonia on day +81. He was treated with broad-spectrum antimicrobials, including cefepime, isavuxonazonium, and caspofungin, for a possible breakthrough fungal infection process while on outpatient voriconazole. On day +87, he developed altered mental status with decreased orientation, inability to follow commands or answer questions appropriately, and intermittent agitation; he did not have any change in level of alertness. Brain MRI did not show evidence of infarction, hemorrhage, or mass; there was were, however, advanced chronic white matter microangiopathic changes. On day +88, he no

longer answered questions or responded to commands. Given concern for cefepime-induced neurotoxicity, he was switched to piperacillin-tazobactam, with no change in clinical status. EEG showed generalized periodic discharges with triphasic morphology and generalized delta/theta slowing concerning encephalopathy. He was started on levetiracetam due to an increased seizure risk. Cerebrospinal fluid showed normal cell count, with negative cytology and infectious studies. On day +89, he became somnolent and did not follow commands, answer questions, or attempt to communicate verbally or nonverbally. Simultaneously, he was noted to have transaminitis (peaked levels; ALT 105 IU/L, AST 178 IU/L, and normal bilirubin), rising eosinophilia with a peak absolute eosinophil count of 1.0 x 10^9/L, and erythematous skin lesions on his scalp, which biopsy showed vacuolar interface dermatitis consistent with GVHD. Due to concerns for recurrent grade 1 skin acute GVHD, he started receiving methylprednisolone 2mg/kg on day +89. Within 48 hours of systemic steroid initiation, his mental status significantly improved; he was alert, followed commands, and was able to answer simple questions verbally. Scalp erythema, transaminitis, and eosinophilia also rapidly resolved. Corticosteroids were tapered slowly, and his mental status returned to baseline. Given his dramatic clinical responsiveness to systemic steroids, we retrospectively annotated his encephalopathy as immune effector cell-associated neurotoxicity syndrome (ICANS), co-occurring with acute GVHD. Inflammation markers, such as CRP or ferritin, were not measured during the acute episodes.

Simultaneous with the onset of GVHD and ICANS, his cytopenias resolved, and a bone marrow examination on day +96 showed decreased myeloblasts to 12% blast by morphology with 100% donor chimerism and normal cytogenetics. An next-generation sequencing panel failed to reveal his previously detected TP53 and IDH1 mutations. A subsequent bone marrow on day +220 demonstrated a flow MRD negative CR with normal karyotype/FISH and 100% donor chimerism in bone marrow.

Patient 6

A 73-year-old male with a history of coronary artery disease, atrial fibrillation, hypothyroidism, interstitial pulmonary fibrosis, and acute myeloid leukemia with complex karyotype including rearrangements of chromosome 3, del(5q), del(7q), trisomy 8 and the loss of 17p and *KRAS/NRAS* mutations received a MUD alloSCT with clofarabine, busulfan, and ATG conditioning. After successful engraftment, he developed progressive thrombocytopenia, and his bone marrow on post-transplant day +142 showed flow an abnormal myeloid population comprising 1.9% of cells. A FISH analysis revealed a del(5q) (23.7%) and loss of 17p (28%), and decreased donor bone marrow chimerism to 80%, all consistent with relapse. He was enrolled into the second cohort of this phase I trial with IFN-γ begun on day 0 and a DLI at +7 day. On day +19, he developed a low-grade fever associated with an elevated CRP of 15.1 mg/dL and a ferritin of 5462 ng/mL. He was admitted to the hospital for an infectious disease workup. Blood cultures and respiratory virus tests were negative. A CT scan showed no definitive infection but showed progression of his known non-specific interstitial pneumonitis with fibrosis, manifested as reticular interstitial opacities. IFN-γ was discontinued on day +23. Despite empiric antibiotics, his intermittent fever persisted. He subsequently developed a skin rash and transaminitis (peaked levels: ALT 169 IU/L, AST 109 IU/L, and a normal bilirubin). Given persistent fever with a progressively increased ferritin level (up to 10750 ng/mL), transaminitis, and new oxygen requirement, grade 2 cytokine releasing syndrome (CRS) was suspected, and he was begun on methylprednisolone 2mg/kg on day +28. While a skin biopsy showed no evidence of GVHD, a liver biopsy on day +28 showed minimal to mild portal and perivenular inflammation containing T lymphocytes associated with increased hepatocyte apoptosis and mild biliary epithelial cell senescence-related changes, favoring low-grade GVHD. Tacrolimus was added on day +32. Subsequently his transaminitis and hypoxia rapidly resolved

with reductions of CRP and ferritin levels. A bone marrow biopsy on day +34 showed a MRD-negative complete remission with 100% donor chimerism, normal FISH, and normal NGS.

Steroids were tapered to prednisone 40mg daily. On day +36, he developed encephalopathy consisting of lethargy and disorientation, with no focal neurological signs. A brain MRI showed a small acute right frontal lobe infarction and non-specific progression of patchy T2 flair periventricular hyperintensities. A CT angiogram of the head and neck angiogram showed mild chronic microvascular changes, no stenosis or occlusion, and patent carotid arteries. The echocardiogram did not show any evidence of an intra-cardiac thrombus. EEG showed rare generalized rhythmic delta and generalized theta activity with intermittent delta slowing, with no epileptiform abnormality, suggesting mild to moderate encephalopathy. The stroke team and neurology considered that the infarct size was too small to explain the clinical manifestation and concluded the MRI findings were likely incidental and not a cause of his encephalopathy. Cerebrospinal fluid was negative for infectious disease, and cytology was negative for malignancy. Tacrolimus was discontinued for possible microangiopathic processes as a culprit for encephalopathy. Simultaneously, he suffered acute recurrent hypoxia and tachypnea requiring 2L/min of oxygen with a CT scan revealing ground grass opacities superimposed on the chronic interstitial disease. He was transferred to the ICU for close observation. Given the risk of complications, bronchoscopy was deferred, and the decision was made to start high dose pulsed methylprednisolone at 1gm/day for 3 days and etanercept twice weekly while covering a broad spectrum of microbes. After the pulsed methylprednisolone, his respiratory and mental status rapidly improved. He was transferred out of the ICU on day +41.

Given the rapid responsiveness to systemic immunosuppressants, we retrospectively annotated his respiratory complications as idiopathic pulmonary syndrome and encephalopathy as ICANS.

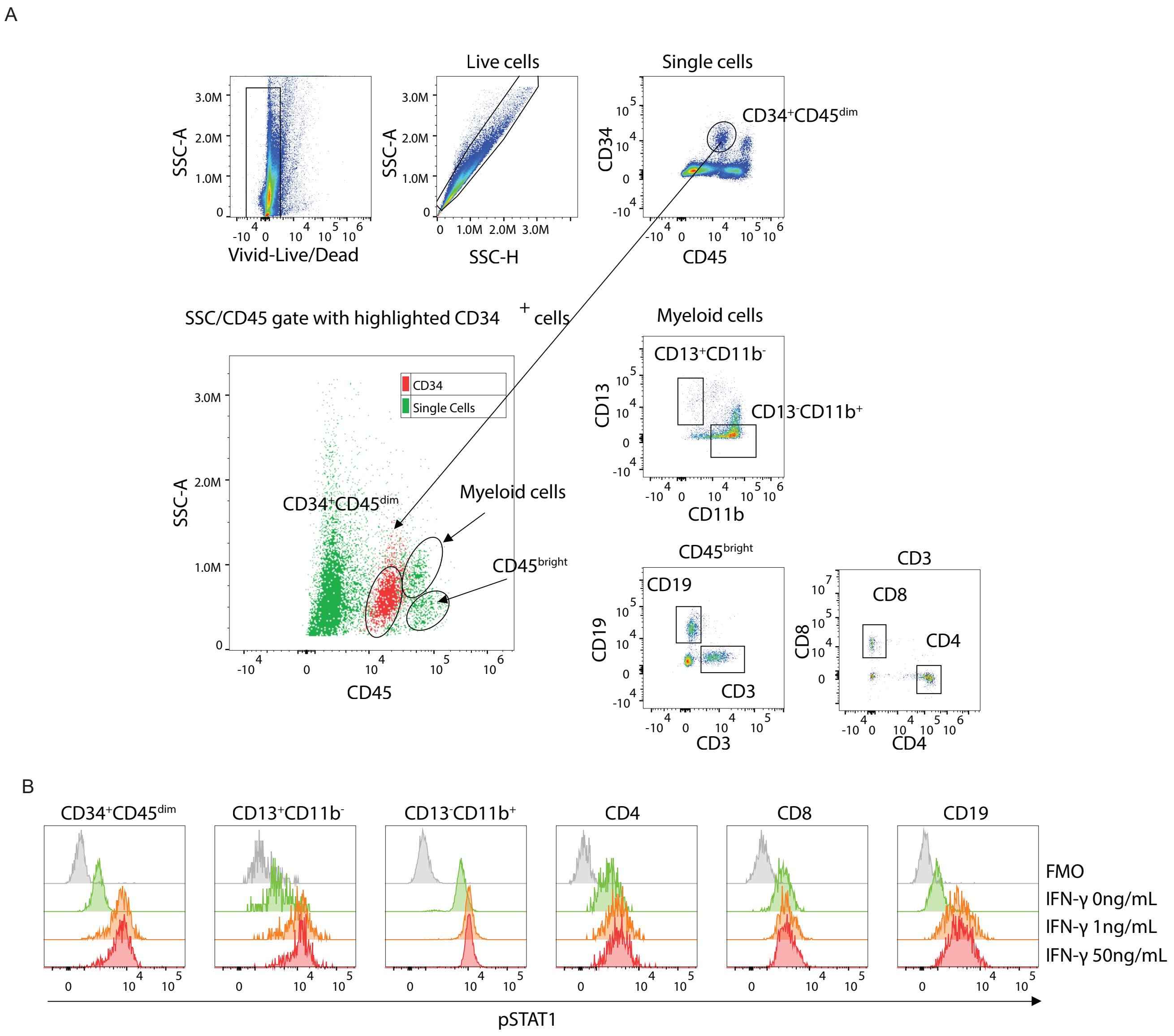
A
Live cells
Single cells
SSC-A
Vivid-Live/Dead
SSC-H
CD34
CD45
CD34+CD45dim
SSC/CD45 gate with highlighted CD34+ cells
CD34
Single Cells
Myeloid cells
CD45bright
CD13+CD11b-
CD13-CD11b+
CD13
CD11b
CD19
CD3
CD8
CD4
B
CD34+CD45dim
CD13+CD11b-
CD13-CD11b+
CD4
CD8
CD19
FMO
IFN-γ 0ng/mL
IFN-γ 1ng/mL
IFN-γ 50ng/mL
pSTAT1

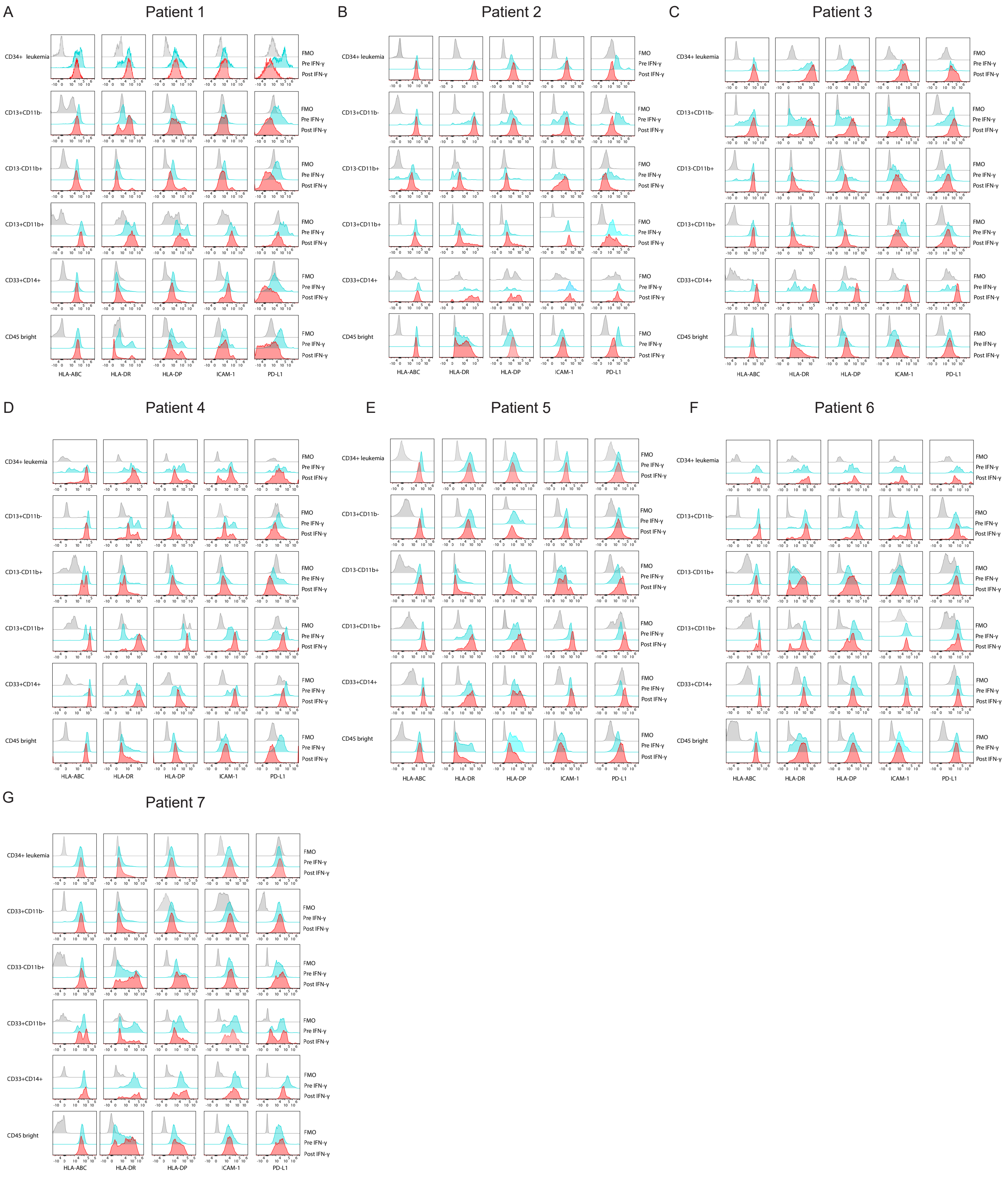
A
Patient 1
B
Patient 2
C
Patient 3
D
Patient 4
E
Patient 5
F
Patient 6
G
Patient 7
CD34+ leukemia
CD13+CD11b-
CD13-CD11b+
CD13+CD11b+
CD33+CD14+
CD45 bright
CD33+CD11b-
CD33-CD11b+
CD33+CD11b+
FMO
Pre IFN-γ
Post IFN-γ
HLA-ABC
HLA-DR
HLA-DP
ICAM-1
PD-L1

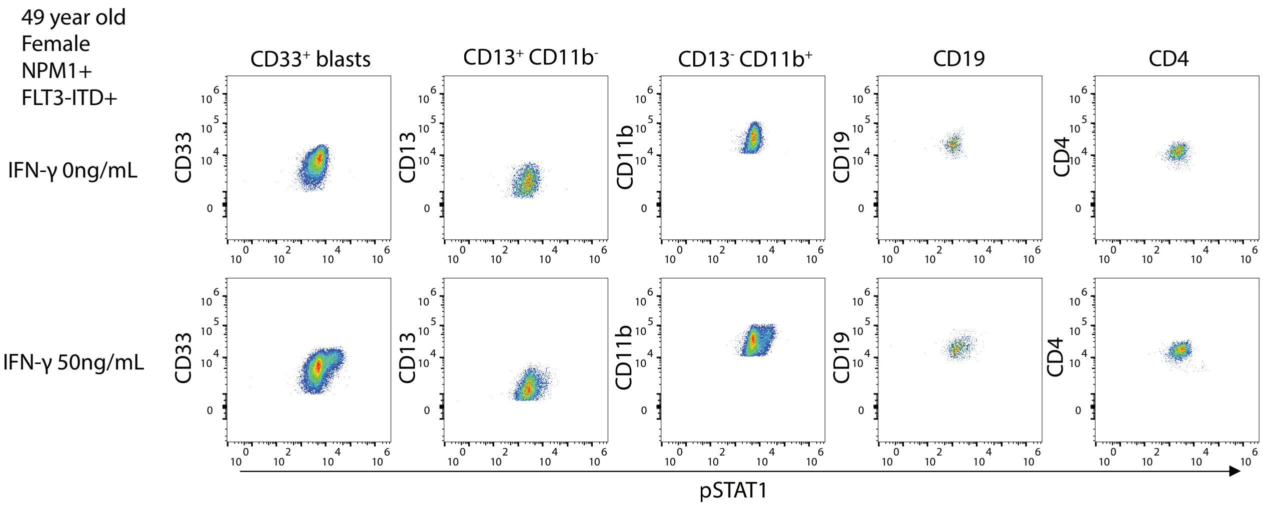
49 year old
Female
NPM1+
FLT3-ITD+
CD33+ blasts
CD13+ CD11b-
CD13- CD11b+
CD19
CD4
IFN-γ 0ng/mL
IFN-γ 50ng/mL
CD33
CD13
CD11b
CD19
CD4
pSTAT1

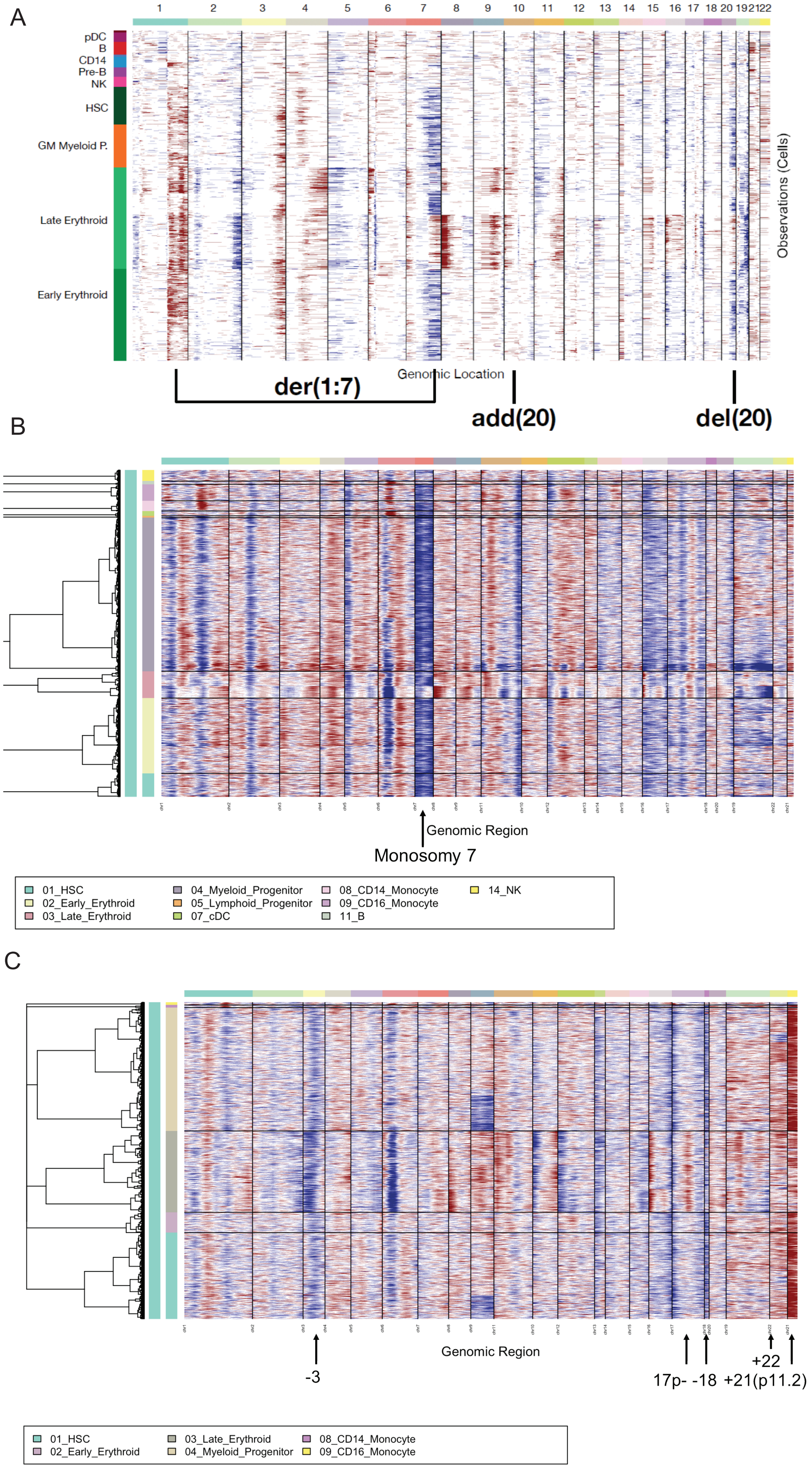
A
1 2 3 4 5 6 7 8 9 10 11 12 13 14 15 16 17 18 20 19 21 22
pDC
B
CD14
Pre-B
NK
HSC
GM Myeloid P.
Late Erythroid
Early Erythroid
Observations (Cells)
Genomic Location
der(1:7)
add(20)
del(20)
B
Genomic Region
Monosomy 7
01_HSC
02_Early_Erythroid
03_Late_Erythroid
04_Myeloid_Progenitor
05_Lymphoid_Progenitor
07_cDC
08_CD14_Monocyte
09_CD16_Monocyte
11_B
14_NK
C
Genomic Region
-3
17p-
-18
+22
+21(p11.2)
01_HSC
02_Early_Erythroid
03_Late_Erythroid
04_Myeloid_Progenitor
08_CD14_Monocyte
09_CD16_Monocyte
