# Supplementary material for: IFN-γ and donor leukocyte infusions for relapsed myeloblastic malignancies after allogeneic hematopoietic stem cell transplantation
Source: JCI Insight. 2025 Mar 25;10(9):e190655. doi: 10.1172/jci.insight.190655 (PMC12128990; doi:10.1172/jci.insight.190655)
Supplement: Supplemental data 1 [file jciinsight-10-190655-s095.pdf]

TITLE: A pilot study of IFN-γ to treat acute myeloid leukemia (AML) and myelodysplastic syndrome (MDS) that has relapsed after allogeneic hematopoietic stem cell transplantation.

**HCC #:**	20-092
**NCT#**	NCT04628338
**Phase:**	Pilot
**Version:**	6.0
**Version Date:**	10-Jan-2023

Investigational Agent: ACTIMMUNE® (interferon gamma-1b) injection

Principal Investigator:

Sawa Ito, M.D., Ph.D.
Assistant Professor
Division of Hematology-Oncology
University of Pittsburgh
5115 Centre Ave, 2nd Floor, Pittsburgh, PA 15232
Phone: 412-623-1416
Fax:412-623-4840
e-mail address: itos3@upmc.edu

Associate Investigator:

Emily Geramita, M.D., Ph.D.
Clinical Fellow
Division of Hematology-Oncology
University of Pittsburgh
5115 Centre Ave, Room 463, PA 15232
Phone: 301-792-3297
e-mail address: geramitae@upmc.edu

Elizabeth Krakow, M.D.
Assistant Professor, Clinical Research Division
Fred Hutchinson Cancer Research Center
1100 Fairvew Ave N, Seattle, WA 98115
Phone: 206-667-3410
Fax: 206-667-2535
e-mail address: efkrakow@fredhutch.org

Warren Shlomchik, M.D.
Professor of Medicine and Immunology, Director, Hematopoietic Stem Cell Transplant and Cell Therapy, Vice-Chief of Hematologic Malignancies, Hematopoietic Stem Cell Transplantation and T cell Immunotherapy, Division of Hematology and Oncology, Department of Medicine, University of Pittsburgh

Geoffrey Hill, M.D. Ph.D.
José Carreras/E. Donnall Thomas Endowed Chair for Cancer Research, Clinical Research Division
Director of The Immunotherapy Integrated Research Center, Fred Hutchinson Cancer Research Center
Director of Hematopoietic Stem Cell Transplantation,

Seattle Cancer Care Alliance

Source(s) of Support: University of Pittsburgh Medical Center
Fred Hutchinson Cancer Research Center
Horizon Pharma USA

PROTOCOL SYNOPSIS

Protocol Title: A pilot study of IFN-γ to treat acute myeloid leukemia (AML) and myelodysplastic syndrome (MDS) that has relapsed after allogeneic hematopoietic stem cell transplantation.

Short title: IFN-γ for post-transplant relapsed AML/MDS

Rationale: Allogeneic hematopoietic stem cell transplantation (alloSCT) can cure patients with acute myeloid leukemia (AML) and myelodysplastic syndrome (MDS). However, relapsed AML/MDS is the most significant single cause of treatment failure, and the majority of relapsed patients ultimately succumb. Alloreactive T cells in the donor graft can kill residual leukemia cells, mediating the graft-vs-leukemia (GVL) effect. Consistent with this, recipients of T cell-depleted grafts have higher rates of relapse. GVL is more potent against chronic leukemias than acute myeloblastic diseases, and the higher incidence of relapse in patients with AML/MDS reflects a failure in GVL. Dr. Shlomchik's laboratory discovered a mechanism to explain the GVL-resistance of myeloblastic leukemias. He reported that chronic phase CML cells are killed by alloreactive T cells even if the leukemia cells cannot respond to IFN-γ, whereas acute myeloblastic leukemias require the IFN-γ receptor to be optimally susceptible to the GVL effect. Moreover, the administration of exogenous IFN-γ could rescue the defective GVL mediated by IFN-γ knock out T cells. Other groups have reported clinical data suggesting an important role of IFN-γ in GVL. Human leukocyte antigen (HLA) expression was reduced in AML samples from patients who relapsed post-alloSCT, relative to their pretransplant samples. This expression could be restored with in vitro IFN-γ treatment. These preclinical and clinical data support our hypothesis that IFN-γ will promote GVL in patients with AML/MDS that has relapsed after alloSCT. The central goal of this pilot phase 1 trial will be to explore whether IFN-γ in this setting is safe and whether it has the desired biological activities on malignant blasts in vivo. We will test IFN-γ in relapsed patients as monotherapy and in conjunction with donor leukocyte infusions (DLI). The clinical and biological information from this study is essential to design a phase II trial with a therapeutic endpoint.

Objectives:
Primary Objectives:

1) Biological objective: To study whether the standard dose of IFN-γ is sufficient to upregulate HLA I, HLA II, ICAM-1, and induce phosphorylated-STAT1 in malignant blasts in vivo in patients who have relapsed after allogeneic stem cell transplantation.

2) Feasibility and safety objective: To explore the feasibility and safety of administering IFN-γ post alloSCT as monotherapy and combined with DLI in patients who have tolerated IFN-γ monotherapy.

Secondary Objectives:

1) Evaluate whether there is a reduction in malignant blast burden coincident with therapy.

2) Evaluate whether there is a progression of stable graft-versus-host disease (GVHD) or development of de novo GVHD coincident with therapy.

Essential eligibility criteria:

a) Recipients of HLA matched allogeneic hematopoietic stem cell transplant with bone marrow or peripheral blood stem cell graft with relapsed AML or MDS.
b) At least 0.5% bone marrow myeloblasts, before or after cytoreductive chemotherapy.
c) no increases in systemic immunosuppressive medications (other than to maintain target blood levels) of >50% in the prior four weeks to treat GVHD,
d) If active grade I or II acute GVHD, no addition of new systemic immunosuppressive medications for the prior two weeks,
e) If a systemic corticosteroid is used, the dose should be at or less than 0.5mg/kg/day prednisone or equivalent.

Study design (Figure 1): This is an investigator-initiated pilot phase I study to evaluate the safety and biological effects of IFN-γ on adult patients with AML/MDS who relapsed after alloSCT. In stage 1, the safety and biological effect of a fixed-dose of IFN-γ (100mcg subcutaneously three times per week) will be evaluated. Bone marrow biopsies will be performed before and no earlier than 48 hours to 1 week after the first dose of IFN-γ to assess malignant blasts for an IFN-γ response based on predicted upregulation in the expression of HLA-class I, II, ICAM-1, and induction of phosphorylated-STAT1 (p-STAT1) on malignant blasts by flow cytometry. We may also perform single-cell RNAseq on samples pre and post IFN-γ treatment. In stage 2, subjects for whom donor leukocytes are available and who have tolerated IFN-γ will receive DLI in a dose-escalating manner as is already a clinical standard of care. IFN-γ will be continued three times a week for four weeks after the first dose of DLI, followed by once-weekly IFN-γ for four weeks. If DLI is not available, the subjects will continue IFN-γ for eight more weeks. The patients will be off treatment after a 12 week-course of IFN-γ with a repeat bone marrow biopsy at this point. The clinical outcomes will be monitored at six months after the first dose of IFN-γ and then the subjects will be off study. Figure 1 summarizes the overall study design.

Cohort 2: As we confirmed the safety of IFN-γ monotherapy in the first cohort, we will evaluate a second cohort wherein we will shorten IFN-γ monotherapy (stage 1) to 1 week before DLI. In stage 1, we will assess the safety a fixed-dose of IFN-γ (100mcg subcutaneously three times per week) for one week. The bone marrow biopsies will be performed before and no earlier than 48 hours to 1 week after the first dose of IFN-γ to assess malignant blasts for an IFN-γ response. In stage 2, subjects who have tolerated IFN-γ will receive DLI in a dose-escalating manner. IFN-γ will be continued three times a week for seven weeks after the first dose of DLI, followed by once-weekly IFN-γ for four weeks. The patients will be off treatment after a 12 week-course of IFN-γ with a repeat bone marrow biopsy at this point. The clinical outcomes will be monitored at six months after the first dose of IFN-γ and then the subjects will be off study. Figure 2 summarizes the study design for cohort 2.

Sample size: Our goal is to have at least three patients receive at least one dose of DLI. For example, if the first three patients all complete stage 1 and stage 2, this initial pilot study would be completed with three patients. We estimate enrolling six total patients, but not likely more than nine, as some patients may not be able to complete the study due to the development of rapid disease progression or unavailability of DLI. Based on the nature of the transplant populations at our centers, we estimate accrual will be completed within six months.

For cohort 2, we aim to enroll six patients receiving IFN-γ and at least one dose of DLI.

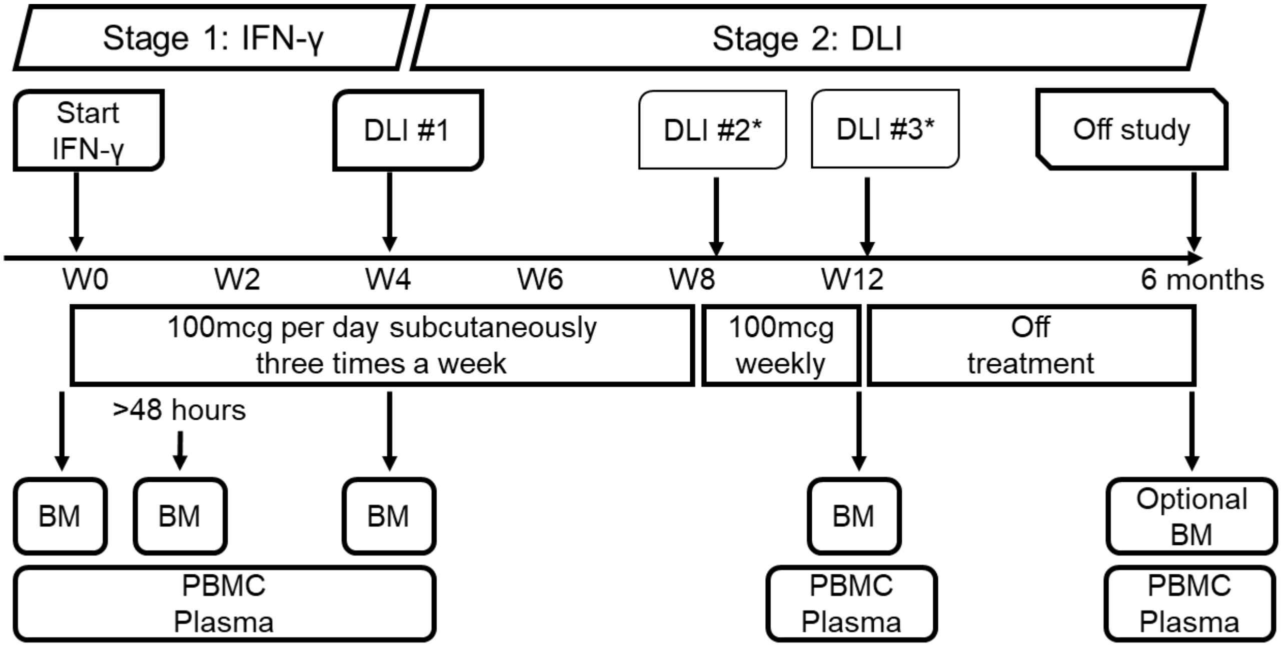

Figure 1. Study Design of Cohort 1: The figure outlines the scheme of how to administer IFN-γ and subsequent donor lymphocyte infusions after enrollment. Biospecimens will also be collected, as shown. BM: bone marrow aspirates, DLI: donor lymphocyte infusion, IFN-γ: interferon-gamma, PBMC: peripheral blood mononuclear cells

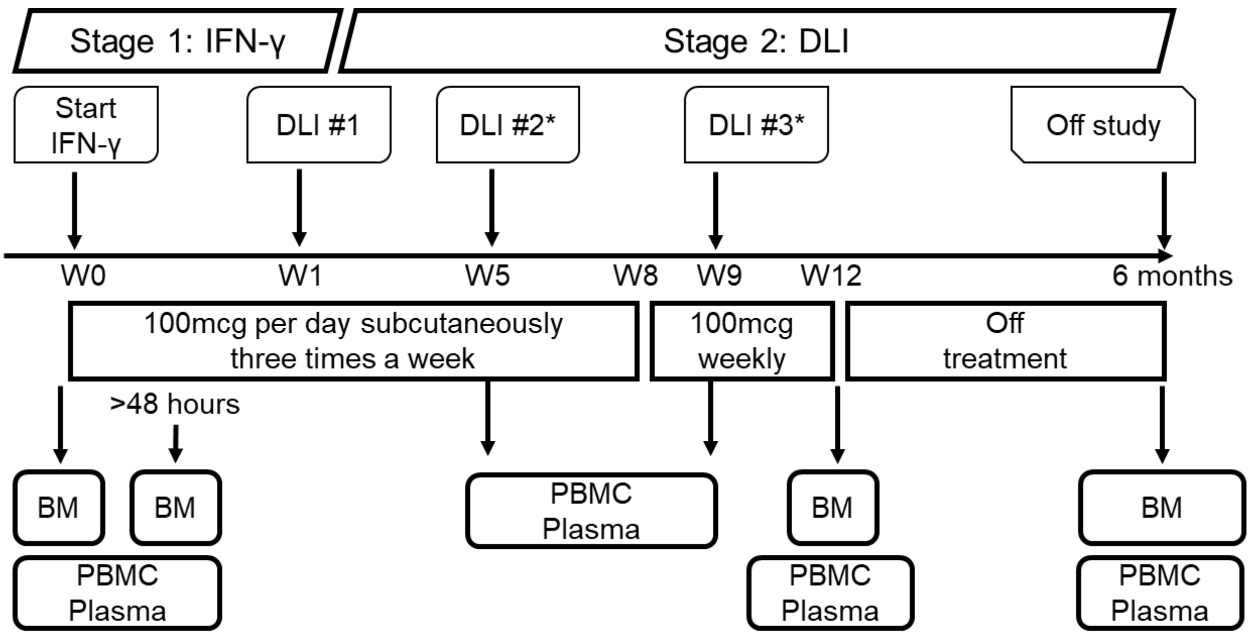

Figure 2. Study Design of Cohort 2: The figure outlines the scheme of how to administer IFN-γ and subsequent donor lymphocyte infusions after enrollment. Biospecimens will also be collected, as shown. BM: bone marrow aspirates, DLI: donor lymphocyte infusion, IFN-γ: interferon-gamma, PBMC: peripheral blood mononuclear cells

TABLE OF CONTENTS

1. OBJECTIVES 9
  1.1 Primary Objectives 9
  1.2 Secondary Objectives 9

2. BACKGROUND 9
  2.1 Role of IFN-γ Therapy for the Relapse after Allogeneic Stem Cell Transplantation 9
  2.2 The Rationale of Dose Selection and Clinical Study Design of IFN-γ after AlloSCT 10
  2.3 Should IFN-γ be effective, there will be a meaningful clinical impact 14
  2.4 Scientific impact of this clinical trial 14

3. PATIENT SELECTION 14
  3.1 Eligibility Criteria 14
  3.2 Exclusion Criteria 15
  3.3 Exclusion of children 16
  3.4 Exclusion of pregnant or breast-feeding women 16

4. RECRUITMENT AND REGISTRATION PROCEDURE 16
  4.1 Recruitment 16
  4.2 Registration 16

5. STUDY AGENTS INFORMATION 17
  5.1 Description 17
  5.2 Formulation and dose 18
  5.3 Pharmacokinetics 18
  5.4 Toxicity 18
  5.5 Clinical adverse events 19
  5.6 Storage 20

6. TREATMENT PLAN AND STUDY PROCEDURES 20
  6.1 Clinical Data Collection 20
  6.2 Pre-Treatment Clinical Evaluation (within 2 weeks of treatment unless specified) 20
  6.3 Stage 1: Safety evaluation of a fixed dose of IFN-γ 22
  6.4 Stage 2: Donor Lymphocyte Infusion 24
  6.5 Protocol Visit, Monitoring, and Biospecimen Collection 27
  6.6 Management of Complications 33
  6.7 Criteria for Discontinuation of IFN-γ 34
  6.8 Management of hypersensitivity reactions to IFN-γ 34
  6.9 Management of Other Complications 34
  6.10 Collection of Biospecimens 35
  6.11 Biospecimen Management and Research Data Collection 36

7. LABORATORY RESEARCH STUDIES 36
7.1 Response of malignant blasts to IFN-γ 36
7.2 Cellular and humoral immune response to IFN-γ 36

8. STATISTICAL CONSIDERATIONS 37
8.1 Study Endpoint 37
8.2 Sample Size 37
8.3 Off Study Criteria 38
8.4 Criteria for Study Suspension 39

9. ADVERSE EVENTS: LIST AND REPORTING REQUIREMENTS 39
9.1 Definitions 39
9.2 Expected Adverse Events 40
9.3 Reporting of Serious Adverse Events 41
9.4 Reporting adverse events to the responsible IRB 42
9.5 Time Period for Collecting Adverse Events 42

10. DATA MANAGEMENT PLAN 43
10.1 Clinical Data Management 43
10.2 Genomic Data Management 43
10.3 End of Study Procedures 43
10.4 Loss or Destruction of Data 43
10.5 Incidental/Secondary Findings Disclosure Procedure 43

11. RISK AND BENEFIT 44
11.1 Risks Related to Interferon-gamma (greater than minimal risk) 44
11.2 Risks Related to Saliva Collection (negligible risk) 44
11.3 Risks Related to Phlebotomy or Bone Marrow Examination (minimal risk) 44
11.4 Risks Related to Nucleotide Sequencing (minimal risk) 44
11.5 Risks Related to Protection of Confidentiality (minimal risk) 45

12. COST AND PAYMENT 45
12.1 Payment and Compensation 45

13. DATA SAFETY MONITORING PLAN 45

14. REFERENCES 47

15. APPENDIX 50
15.1 Appendix A. CIBMTR acute GVHD grading and staging system 50
15.2 Appendix B. NIH chronic GVHD scoring system 51
15.3 Appendix C. Schedule of event for cohort 1 56
15.4 Appendix D. Schedule of event for cohort 2 57

1. OBJECTIVES

1.1 Primary Objectives

1.1.1 Biological objective: To study whether the standard dose of IFN-γ is sufficient to upregulate HLA I, HLA II, ICAM-1, and induce phosphorylated-STAT1 in malignant blasts in vivo in patients who have relapsed after allogeneic stem cell transplantation (alloSCT)

1.1.2 Feasibility and safety objective: To explore the feasibility and safety of administering IFN-γ monotherapy post alloSCT and combined with DLI in patients who have tolerated IFN-γ monotherapy.

1.1.3 Primary Endpoints

1.1.3.1 Primary biological endpoint:

- Upregulation of key targets including HLA-ABC and -DR/DQ, ICAM-1 and generation of phosphorylated STAT1 (pSTAT1) in bone marrow malignant blasts post-IFN-γ treatment, measured by the changes in mean florescent intensity and percentage of positive cells by flow cytometry

1.1.3.2 Primary safety endpoints:

- To evaluate the adverse events of IFN-γ in relapsed patients after alloSCT per CTCAE v5.0

- To evaluate the adverse events of IFN-γ in relapsed patients after alloSCT with concurrent use of donor lymphocyte infusion per CTCAE v5.0

1.2 Secondary Objectives

1.2.1 Evaluate whether there is a reduction in malignant blast burden coincident with therapy.

1.2.2 Evaluate whether there is a progression of stable graft-versus-host disease (GVHD) or development of de novo GVHD coincident with therapy.

1.2.3 Secondary Endpoints

- Reduction of malignant blasts after IFN-γ therapy and subsequent DLI
- Incidence of graft-versus-host disease (GVHD) progression after IFN-γ therapy and subsequent DLI
- Incidence of de novo GVHD after IFN-γ therapy and subsequent DLI

2. BACKGROUND

2.1 Role of IFN-γ Therapy for the Relapse after Allogeneic Stem Cell Transplantation

AlloSCT is a potentially curative therapy commonly prescribed for patients with moderate- to high-risk acute myeloid leukemia (AML) and myelodysplastic syndrome (MDS). The progeny of

alloreactive αβ T cells in the donor graft can attack recipient leukemia cells, thereby mediating the graft-vs-leukemia effect (GVL).[1] However, GVL is not consistently effective, and relapsed disease remains the single most common cause of death after alloSCT.[2] The mechanism of GVL-resistance and sensitivity are heterogeneous dependent on the types of hematologic malignancies. For example, chronic-phase chronic myelogenous leukemia (CP-CML) is exquisitely GVL-sensitive, whereas blast crisis CML (BC-CML) is GVL-resistant, despite sharing common biology. [3-6] Likewise, AML is relatively GVL-resistant.[7] Dr. Shlomchik's lab established GVL models against mouse CP-CML (mCP-CML) and BC-CML (mBC-CML) created by the retroviral transfer into bone marrow (BM) cells of disease-defining oncogenes.[8-10] As is the case in the clinic, mCP-CML is very GVL-sensitive whereas mBC-CML is relatively GVL-resistant. Gene deficient leukemias were created to screen for mechanisms of GVL-resistance. The key finding was that mBC-CML required the expression of the IFN-γR for both CD4 and CD8-mediated GVL. In contrast, STAT1/STAT2$^{-/-}$ mCP-CML, which cannot respond to any type of IFN, was fully GVL-sensitive.[10] Our results suggest that effective GVL against myeloblastic leukemias requires a high magnitude of alloreactive T cell response that generates IFN-γ whereas a low level smoldering alloreactive T cell response may be sufficient to control CP-CML. IFN-γR$^{-/-}$ AML created by retrovirus transduction with the MLL-AF9 fusion was also GVL resistant. In subsequent unpublished work, Dr. Shlomchik found that infusion of IFN-γ can, in part, rescue the defective GVL mediated by IFN-γ$^{-/-}$ T cells.

Compelling clinical data support an important role for IFN-γ. Several groups have reported a lower rate of AML relapse in patients who have early CMV-reactivation, which results in NK cell- and T cell-derived IFN-γ production.[11,12] HLA class I expression was reduced in AML samples from patients who relapsed post-alloSCT, relative to their pretransplant samples, which was restored with in-vitro IFN- γ treatment. [13,14]

In summary, these preclinical and clinical data support our hypothesis that IFN-γ will promote GVL in patients with AML/MDS that has relapsed after alloSCT. The central goal of this pilot phase 1 trial will be to study whether IFN-γ is safe and if it has the desired biological activities on AML blasts in vivo. Such information is essential to support a phase II trial with a therapeutic endpoint.

2.2 The Rationale of Dose Selection and Clinical Study Design of IFN-γ after AlloSCT

The proposed study is an investigator-sponsored study with a primary objective to study a safe dosing regimen IFN-γ that is sufficient to stimulate IFN-γ receptors on malignant blasts. Patients who developed relapsed AML or MDS after alloSCT with no active or history of III-IV acute GVHD would be eligible. This pilot study has two stages: 1) Evaluation of safety of fixed-dose of IFN-γ (ACTIMMUNE) and 2) Fixed doses of IFN-γ with a donor lymphocyte infusion (DLI).

IFN-γ (ACTIMMUNE; interferon-gamma-1b) for this clinical trial will be supplied by Horizon Therapeutics USA. ACTIMMUNE is FDA approved to reduce the frequency and severity of serious infections associated with chronic granulomatous disease (CGD) and to delay disease progression in patients with severe, malignant osteopetrosis.

A review of the literature supports the safety of IFN-γ administration in humans. In CGD, patients (n=63), interferon-gamma-1b was well tolerated with common adverse events including fever (82%), headache (52%), rash (27%), chills (22%), injection site erythema or tenderness (22%), fatigue (22%), diarrhea (22%), vomiting (21%), nausea (16%), myalgia or arthralgia (12.6%). In the dose titration strategy in healthy volunteers,[15] 40 healthy volunteers were

randomized to receive three-week treatment of interferon-gamma-1b three times a week with either no titration (50 mcg/m2) or titration dosing (15mcg/m2 during week 1, 30mcg/m2, during week 2, and 50mcg/m2 during week 3). The severity of flu-like symptoms was reduced in the dose titration cohort only at 8 hours but not at a 1-week or 3-week treatment period, suggesting the subjects can develop rapid tolerance to the symptoms associated with interferon-gamma-1b. Adverse events observed in the no-titration cohort included injection site erythema (57%), fever (54%), fatigue (23%), flu-like symptoms (23%), headache (57%), dizziness (57%), myalgia (14.3%), and nausea (17%).

Ten clinical trials are investigating IFN-γ in cancer patients with two studies actively recruiting. In a study of soft tissue sarcoma (NCT01957709), eight patients received IFN-γ 100 mcg/m2 subcutaneously once a week for four weeks or three times a week for two weeks before the surgery. All patients tolerated IFN-γ well with no serious adverse event. The observed adverse events include flu-like symptoms (62.5%), fatigue (62.5%), chills (50%), fever (25%), injection site pain (25%), dry mouth (12.5%), diarrhea (12.5%), and night sweat (12.5%), headache (50%), dizziness (12.5%), body aches (25%), arthralgia (12.5%). The investigators reported increased expression of HLA class I and II on tumor cells biopsied two weeks after IFN-γ treatment relative to pretreatment biopsies.

The safety and toxicities of DLIs have been well documented in multiple clinical trials.[4,7] Cytokine therapies such as IFN-α, IL-2, and IL-15 have also been safely administered in combination with DLIs in patients with relapsed myeloid leukemia post-alloSCT.[16-18]

In the present trial, we will initiate treatment at 100mcg (almost equal to the dose of 50 mcg/m2 for an adult) three times a week, with the potential to deescalate the frequency of injection for unacceptable toxicity. To explore whether this dosing regimen is sufficient to activate myeloblasts, we will harvest pre- and post-treatment bone marrow specimens to analyze for IFN-γ action (upregulation of HLA class I; HLA class II, ICAM-1 and phosphorylation of STAT1). The primary safety concern is the development of GVHD, which is routinely monitored for all alloSCT patients.

Since the protocol was open for accrual in June 2021, we enrolled three patients with relapsed AML/MDS in this trial. (Table 1). All three patients tolerated IFN-γ monotherapy well with anticipated minor adverse events, including expected injection site reactions, flu-like symptoms, and stable cytopenia (Table 2). Two patients who received DLI combined with IFN-γ developed the steroid-responsive GVHD, concurrently with a dramatic improvement in donor chimerism and complete disease remission (Figure 3). One patient who did not have DLI showed disease progression; however, the patient eventually showed hematological improvement without subsequent salvage chemotherapy. All patients are alive and achieved transfusion independence. These results confirmed the safety of IFN-γ monotherapy in alloSCT for the first time in human trials and possible efficacy of IFN-γ with DLI combination, for the post-transplant relapse, where no standard care has been established in the current practice.

Thus we will propose a second cohort wherein we will shorten IFN-γ monotherapy from 4 weeks to 1 week before DLI. This will allow subjects to get DLI earlier and enable full dose IFN-γ post-DLI. Should this new approach be safe and effective, this would be the platform we move forward into a phase II trial with an efficacy endpoint.

Table 1. Clinical characteristics of the subjects.

Subject ID	20-092-001	20-092-002	20-092-003
Age/gender	66 Male	67 Male	64 Male
Primary disease	MDS/RAEB-II	t-MDS/RAEB-II	AML
Cytogenetics/molecular abnormality	der(1;7),add(10),del(20) DNMT3A, U2AF1	Monosomy 7, t(3;8)	Complex cytogenetics TP53 x2, IDH1
Donor, stem cell source	MUD, PBSC	MUD PBSC	MUD PBSC
Conditioning regimen	Flu/Bu2/ATG	Flu/Bu2/ATG	Flu/Bu2/ATG
Time to relapse	256 days	98 days	33 days
Post-relapse treatment before study enrollment	None	Azacitidine, decitabine, venetoclax	Decitabine, venetoclax, ivosidenib
Pre-IFN-γ bone marrow blast (%)	10%	4%	29.5%
DLI CD3 dose and number	1 x 10e7/kg x once	Not available	1 x 10e7/kg x once
Post- IFN-γ survival	Alive, 202 days	Alive, 124 days	Alive, 128 days

AML, acute myeloid leukemia; ATG, anti-thymocyte globulin; BM, bone marrow; Bu, busulfan; DLI, donor lymphocyte infusion; Flu, fludarabine; GVHD, graft versus host disease; MDS, myelodysplastic syndrome; MUD, matched unrelated donor; PBSC, peripheral blood stem cells; RAEB, refractory anemia with excess blasts; WB, whole blood.

Table 2. Summary of adverse events in the first three patients.

	IFN-γ monotherapy		IFN-γ+ DLI	
Adverse event	All grade	Grade> 3	All grade	Grade> 3
Acute GVHD	0	0	2	1
Sepsis	1	1	0	0
Febrile neutropenia	0	0	1	1
Bacteremia	0	0	1	0
Anemia	3	2	2	1
Decreased platelets	2	2	2	2
Decreased ANC	1	1	2	2
Decreased ALC	3	3	2	2
Flu like symptoms	3	0	0	0
Injection site reaction	2	0	0	0
Malaise	1	0	0	0
Myalgia	1	0	0	0
Fatigue	0	0	1	0
Sinus tachycardia	1	0	0	0
Hypotension	1	0	0	0
Abdominal pain	0	0	1	0
Diarrhea	0	0	2	1
Dry skin	0	0	1	0
Nausea	1	0	2	0

Vomiting	0	0	1	0
GI other: rectal bleed	0	0	1	0
Headache	1	0	0	0
Itchy eyes- intermittent	1	0	0	0

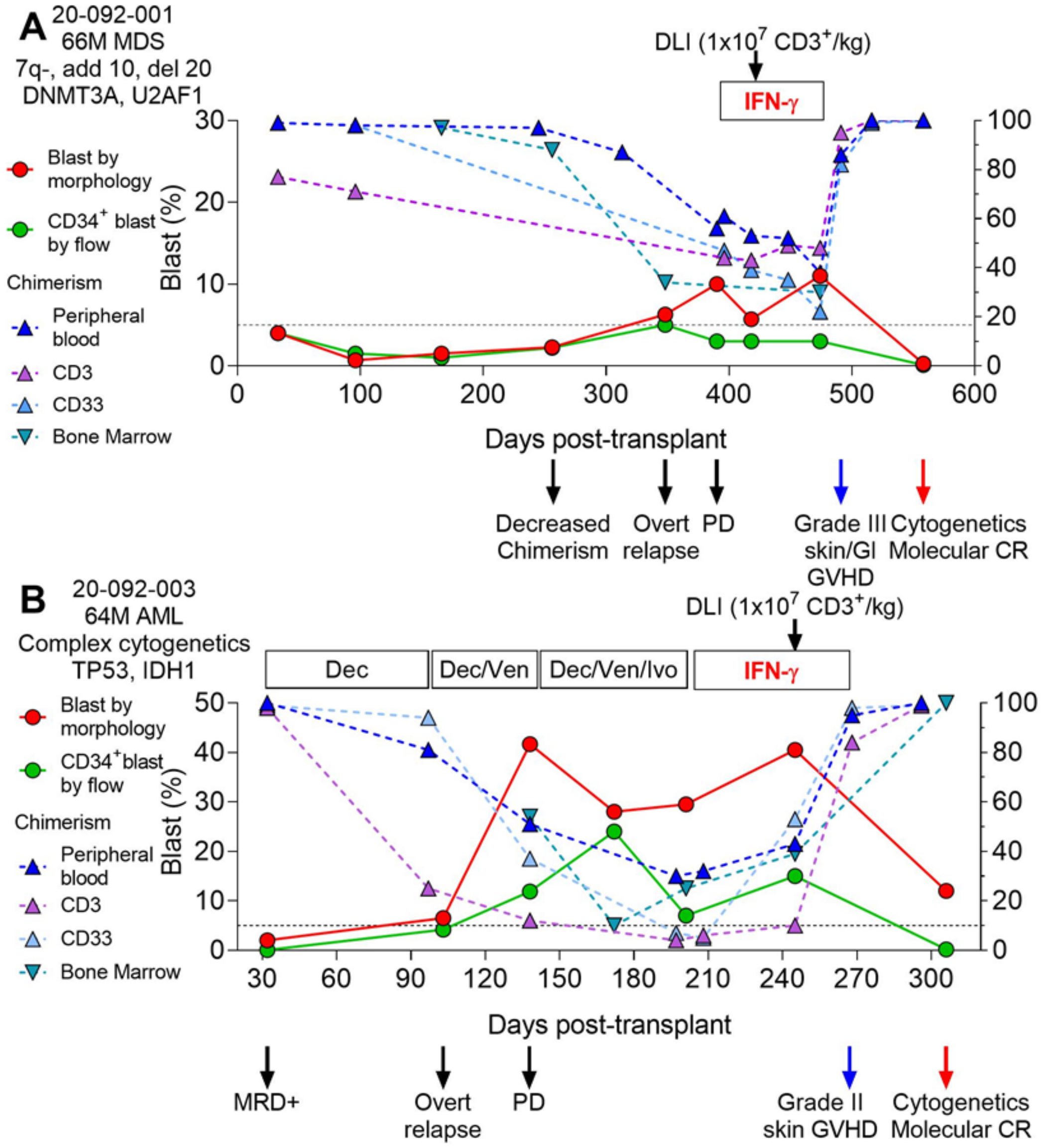

Figure 3. Clinical responses to combined IFN-γ and DLI therapy. A. Case 20-092-001. 66-year-old male with MDS (RAEB II, chromosome 7q and 20 deletions with DNMT3A and U2AF2 mutations) who received matched unrelated donor (MUD) alloSCT, showed decreased bone marrow chimerism on post-transplant day 256. Subsequent bone marrow on day 348 showed overt relapse with 10% abnormal myeloid blasts. He received IFN-γ monotherapy for 4 weeks, followed by one-time DLI with CD3 dose of 1x107/kg. At 8 weeks after DLI, he developed skin and GI GVHD (grade III), with the dramatic improvement of donor chimerism. His bone marrow biopsy on post-transplant day 558 (post-IFN-γ day 168) showed complete cytogenetic and molecular remission with complete hematologic recovery. B. Case 20-092-003. 64-year-old male with AML (complex cytogenetics, TP53, and IDH1 mutations) had bone marrow biopsy showing positive minimal residual disease by flow cytometry as early as day 30 after MUD alloSCT. Despite immediate salvage treatment, his disease progressed and was refractory to decitabine, venetoclax, and ivosidenib (IDH1 inhibitor). He discontinued all salvage therapies and subsequently received IFN-γ and one dose DLI. 3 weeks

after DLI, he developed grade II skin GVHD and increased donor chimerism. His bone marrow biopsy on post-transplant day 306 (post-IFN-γ day 92) showed 100% donor chimerism with complete remission with clearance of cytogenetics and molecular changes. He became transfusion independent, and GVHD was completely resolved with steroid and tacrolimus. Abbreviation: AML, acute myeloid leukemia; CR, complete remission; Dec, decitabine; DLI, donor lymphocyte infusion; Flu, fludarabine; GVHD, graft versus host disease; Ivo, ivosidenib (IDH1 inhibitor); PD, progression of disease; MDS, myelodysplastic syndrome; MRD, measurable residual disease; Ven, venetoclax;

2.3 Should IFN-γ be effective, there will be a meaningful clinical impact

Over decades of clinical alloSCT, there has been little progress in addressing leukemia relapse outside of donor leukocyte infusions (DLI), which is effective against chronic phase CML, but which has limited efficacy against relapsed acute leukemias and MDS. Up to 50% of post-transplant deaths are secondary to relapse. While there is a GVL effect against AML and ALL[3,19,20], relapse rates are higher than for chronic leukemias. No more than 20% of patients with relapsed AML after alloSCT become long-term survivors, and only a small minority can be successfully rescued by DLI[4,6,21-27]. The magnitude of the relapse problem is reflected in the staging of NCI-funded conferences on relapse post-transplant[28-30]. Despite efficacy, many patients with transplantable malignancies are not offered an alloSCT. In 2018 nearly 8800 alloSCTs were performed in the US and 32,000 worldwide in 2012[31], the majority for hematologic malignancies (3000 in the US for AML). Yet, in 2019[32] within the US alone, there will be an estimated 75,000 diagnoses of potentially transplantable leukemias, including 21,450 and up to 42,000 new cases of AML and MDS, respectively. The decision to refer a patient for alloSCT depends on assessing the degree to which alloSCT reduces the risk of relapse relative to treatment-related morbidity and mortality, chiefly GVHD, and infection. This calculation would change if GVL were more potent. Considering these data, successful approaches for augmenting GVL would have a major public health impact.

2.4 Scientific impact of this clinical trial

This pilot study will not be powered for efficacy, though leukemia/MDS burden will be followed as a secondary endpoint. Nonetheless, because we know that relapsed leukemia/MDS always progresses without intervention, any significant reduction in leukemia/MDS burden with IFN-γ, with or without DLI, would be reasonable evidence for efficacy and would support the overall hypothesis underlying the trial. Should we find that IFN-γ can be safely administered at a dose and schedule that stimulates myeloblasts in vivo, this would strongly support the development of a multi-center phase II study with a primary efficacy endpoint. Moreover, we will not just measure IFN-γ concentrations post-injection but will dose based on evidence of IFN-γ action on myeloblasts. We also aim to perform single-cell RNAseq (scRNAseq) on AML cells pre- and post-IFN-γ treatment, which has also never been done. RNAseq will allow us to focus on changes in cells with a transcriptome consistent with their having stem cell properties. To conduct ancillary studies, we plan to collect the sequential biospecimens including saliva, peripheral blood, and bone marrow from the subjects.The longer-term goal is to understand which IFN-γ-induced changes may sensitize myeloblasts to T cell killing. These studies will synergize with work in Dr. Shlomchik's lab wherein the same questions are being addressed in murine leukemias, which can be genome-edited and then tested in GVL models. The identification of the key changes could in the future, lead to the development of therapies more narrowly focused than is IFN-γ.

3. PATIENT SELECTION

3.1 Eligibility Criteria

3.1.1 Ages 18 or older

3.1.2 Recipients of allogeneic stem cell transplantation for acute myeloid leukemia or myelodysplastic syndrome from a human leukocyte antigen (HLA) matched donor

3.1.3 Relapsed of primary disease, prior to or after salvage treatment with at least 0.5% of blasts in the bone marrow by flow cytometry with a clear leukemia-associated immunophenotype.

3.1.4 Performance status KPS score >60% (ECOG 0-2)

3.1.5 No increases in systemic immunosuppression in the prior four weeks other than to maintain therapeutic levels

3.1.6 No systemic corticosteroid with a dose higher than 0.5mg/kg/day prednisone or equivalent

3.1.7 No history of grade IV acute GVHD

3.1.8 No new systemic immunosuppressive medications in the prior two weeks initiated due to GVHD

3.1.9 Willingness to have bone marrow and peripheral blood collected as per the study protocol

3.1.10 Must be able to give informed consent

3.2 Exclusion Criteria

3.2.1 Contraindication to receive IFN-γ including known hypersensitivity to interferon-gamma, E. coli derived products or any component of the product

3.2.2 Subjects with a positive pregnancy test or who are breastfeeding

3.2.3 For men or women of childing bearing potential (age < 50 without hysterectomy or oophorectomy or documented menopause), unwilling to use effective contraception for the duration of the study.

3.2.4 Primary engraftment failure

3.2.5 Active cardiac arrhythmias not controlled by medical management or current NYHA class II or higher congestive heart failure

3.2.6 Active ischemic heart disease not well controlled with medications

3.2.7 A seizure disorder not well controlled by medications

3.2.8 Estimated GFR <30 mL/min

3.2.9 AST/SGOT or ALT/SPOT > 5 x ULN

3.2.10 Total bilirubin > 3 x ULN

3.2.11 Chemotherapy (other than hypomethylating and/or venetoclax therapy, or targeted therapy such as IDH1/2 inhibitor or tyrosine kinase inhibitor) within the prior 4 weeks

3.2.12 Body surface area at or less than 1.5 m^2, or greater than 2.5 m^2 so as to minimize the variation in IFN-γ exposure based on differences in BSA.

3.3 Exclusion of children

Patients less than 18 years of age will not be eligible in this study because of the potentially serious risks and limited information of the study intervention in children under the unique clinical setting after alloSCT. Also, the study investigators are not treating the minor subjects in our respective institution. Should the results of this trial demonstrate efficacy, consideration will be given to expanding accrual to this patient population.

3.4 Exclusion of pregnant or breast-feeding women

There are no adequate studies of IFN-γ (Actimmune) in pregnant or breastfeeding women. It is not known whether IFN-γ (Actimmune) is excreted in human milk. Because we cannot exclude the potential for serious adverse reactions in fetuses or nursing infants, pregnant or breast-feeding women will be excluded from study participation.

4. RECRUITMENT AND REGISTRATION PROCEDURE

4.1 Recruitment

Patients will be primarily recruited through the clinical practitioners of the primary care team or members of the research team who will identify the patients who may be eligible for this protocol. Patients may also be recruited through ClinicalTrials.gov.

4.2 Registration

Patients who meet the eligibility criteria will be consented by the research team. Demographic and clinical data will be collected in the Clinical Trials Management Application (CTMA) system.

External subjects will be registered centrally by the Coordinating Center. External sites must keep a log of subjects who have signed an informed consent document. Each external site must notify the Coordinating Center of subject consent within one business day of the consent. The Coordinating Center will enter the subject and consent date in the internal Clinical Trials Management Application (CTMA) for subject tracking.

External sites must verify subject eligibility prior to submitting screening documents to the Coordinating Center. All eligibility documents must be uploaded to a secure web-based

SharePoint for verification by the Coordinating Center. Relevant screening documents include:

Protocol Registration Form
Copy of the subject signed informed consent form
Eligibility Checklist signed by a First Reviewer and Investigator
All source documents needed to verify eligibility
All results collected per the Schedule of Assessments in Section 7 of the protocol
Source documents supporting stratification factors
As submission of pathology materials for diagnostic review and classification is mandatory, confirmation of tissue availability will be required prior to registration
Coordinating Center supplied source documents, as applicable

All requests for subject registration must be made at least 48 hours prior to the planned start of treatment date. The Coordinating Center will review all submitted documents for accuracy and verify all eligibility criteria are confirmed. The Coordinating Center will notify the external site by email of any discrepancies or requests for additional information, as applicable. A subject will not be registered until all outstanding discrepancies are adequately addressed and eligibility has been confirmed.

Once eligibility is confirmed, the Coordinating Center will register the subject in CTMA. The Coordinating Center will email the subject registration confirmation, including assigned subject ID, to the external site the day of subject registration.

Only eligible subjects will be enrolled. Exceptions to inclusion and exclusion criteria are not permitted. External sites should contact the UPMC HCC Sponsor and Coordinating Center for any questions regarding subject eligibility.

External sites must notify the Coordinating Center of screen failures or withdrawal of consent within one business day of the status change.

5. STUDY AGENTS INFORMATION

5.1 Description

5.1.1 Name: ACTIMMUNE (Interferon gamma-1b), supplied by Horizon Pharma USA

5.1.2 Manufacturing: ACTIMMUNE (Interferon gamma-1b) is a single-chain polypeptide containing 140 amino acids. Production of ACTIMMUNE is achieved by fermentation of a genetically engineered Escherichia coli bacterium containing the DNA which encodes for the recombinant protein. Purification of the product is achieved by conventional column chromatography. ACTIMMUNE is a highly purified sterile solution consisting of non-covalent dimers of two identical 16,465 Dalton monomers, with a specific activity of 20 million International Units/mg (2 x 106 International Units/0.5 mL) which is equivalent to 30 million units/mg.

5.2 Formulation and dose

5.2.1 Dosage form: 100 mcg (2 million International Units) per 0.5 mL solution in a single-dose vial. ACTIMMUNE (interferon gamma-1b) is a sterile, clear, colorless solution filled in a single-dose vial for subcutaneous injection.

5.2.2 Route of administration: Subcutaneous injection

5.2.3 Dose regimen: 100 mcg three times weekly or per protocol guidance based on tolerability, response, or DLI infusions

5.3 Pharmacokinetics

The intravenous, intramuscular, and subcutaneous pharmacokinetics of ACTIMMUNE has been investigated in 24 healthy male subjects following single-dose administration of 100 mcg/m2 (twice the recommended dose for chronic granulomatous disease patients). ACTIMMUNE is rapidly cleared after intravenous administration (1.4 Liters/minute) and slowly absorbed after intramuscular or subcutaneous injection. After intramuscular or subcutaneous injection, the apparent fraction of dose absorbed was higher than 89%. The mean elimination half-life after intravenous administration of 100 mcg/m2 in healthy male subjects was 38 minutes. The mean elimination half-lives for intramuscular and subcutaneous dosing with 100 mcg/m2 were 2.9 and 5.9 hours, respectively. Peak plasma concentrations, determined by ELISA, occurred approximately 4 hours (1.5 ng/mL) after intramuscular dosing and 7 hours (0.6 ng/mL) after subcutaneous dosing. Multiple-dose subcutaneous pharmacokinetic studies were conducted in 38 healthy male subjects. There was no accumulation of ACTIMMUNE after 12 consecutive daily injections of 100 mcg/m2. Interferon-gamma was not detected in the urine of healthy human volunteers following administration of 100 mcg/m2 of ACTIMMUNE by the intravenous, intramuscular, and subcutaneous routes. In vitro perfusion studies utilizing rabbit livers and kidneys demonstrate that these organs are capable of clearing interferon-gamma from perfusate.

5.4 Toxicity

5.4.1 Carcinogenesis: ACTIMMUNE has not been tested for its carcinogenic potential.

5.4.2 Mutagenesis: Ames tests using five different tester strains of bacteria with and without metabolic activation revealed no evidence of mutagenic potential. ACTIMMUNE was tested in a micronucleus assay for its ability to induce chromosomal damage in bone marrow cells of mice following two intravenous doses of 20 mg/kg. No evidence of chromosomal damage was noted.

5.4.3 Fertility: Female cynomolgus monkeys treated with daily subcutaneous doses of 30 or 150 mcg/kg ACTIMMUNE (approximately 20 and 100 times the human dose) exhibited irregular menstrual cycles or absence of cyclicity during treatment. Similar findings were not observed in animals treated with 3 mcg/kg ACTIMMUNE. Female mice receiving recombinant murine IFN-interferon gamma (rmuIFN-gamma) at 32 times the maximum recommended clinical dose of ACTIMMUNE for 4 weeks via intramuscular injection exhibited an increased incidence of atretic ovarian follicles. Male cynomolgus monkeys treated intravenously for 4 weeks, with 8 times the maximum recommended clinical dose of ACTIMMUNE showed decreased spermatogenesis. Male mice receiving rmuIFN-gamma at 32 times the maximum recommended clinical dose of ACTIMMUNE for 4 weeks via intramuscular injection exhibited reduced spermatogenesis. The impact of this finding on fertility is not known. Male mice treated subcutaneously with rmuIFN-gamma from shortly after birth through puberty, with 280 times the maximum recommended clinical dose of ACTIMMUNE exhibited profound yet reversible decreases in sperm counts and fertility and an increase in the number of abnormal sperm. The clinical significance of these findings observed following treatment of mice with rmuIFN-gamma is uncertain.

5.5 Clinical adverse events

5.5.1 Cardiovascular Disorders: Acute and transient “flu-like” symptoms such as fever and chills induced by ACTIMMUNE at doses of 250 mcg/m2/day (greater than 10 times the weekly recommended dose) or higher may exacerbate pre-existing cardiac conditions. Patients with pre-existing cardiac conditions, including ischemia, congestive heart failure, or arrhythmia on ACTIMMUNE, should be monitored for signs/symptoms of exacerbation. Some of “flu-like” symptoms may be minimized by bedtime administration of ACTIMMUNE. Acetaminophen may also be used to ameliorate these effects.

5.5.2 Neurologic Disorders: Decreased mental status, gait disturbance, and dizziness have been observed, particularly in patients receiving ACTIMMUNE doses greater than 250 mcg/m2/day (greater than 10 times the weekly recommended dose). Most of these abnormalities were reversible within a few days upon dose reduction or discontinuation of therapy. Monitoring of patients with seizure disorders or other central nervous system disorders is recommended when administering ACTIMMUNE.

5.5.3 Bone Marrow Toxicity: Reversible neutropenia and thrombocytopenia that can be severe and may be dose-related have been observed during ACTIMMUNE therapy.

5.5.4 Hepatic Toxicity: Repeated administration of ACTIMMUNE to patients with advanced hepatic disease may result in the accumulation of interferon-gamma-1b. Frequent assessment of liver function in these patients is recommended. Elevations of aspartate transaminase (AST) and/or alanine transaminase (ALT) (up to 25-fold) have been observed during ACTIMMUNE therapy.

5.5.5 Hypersensitivity Reactions: Isolated cases of acute serious hypersensitivity reactions have been observed in patients receiving ACTIMMUNE. If such an acute reaction develops, the drug should be discontinued immediately and appropriate medical therapy instituted. Transient cutaneous rashes have occurred in some patients following the injection of ACTIMMUNE that have necessitated treatment interruption.

5.5.6 Renal Toxicity: Monitor renal function regularly when administering ACTIMMUNE in patients with severe renal insufficiency because the possibility exists that with repeated administration, accumulation of interferon gamma-1b may occur. Renal toxicity has been reported in patients receiving ACTIMMUNE.

5.5.7 Allergic Reactions to Natural Rubber: The stopper of the glass vial for ACTIMMUNE contains natural rubber (a derivative of latex), which may cause allergic reactions.

5.6 Storage

The vials should be stored in the refrigerator at 2 to 8 °C (36 °F to 46 °F). Do Not Freeze. Avoid excessive or vigorous agitation. Do Not Shake. An unused vial of ACTIMMUNE can be stored at room temperature up to 12 hours prior to use. Discard vials if not used within the 12-hour period. Do not return to the refrigerator

6. TREATMENT PLAN AND STUDY PROCEDURES

6.1 Clinical Data Collection

Each subject will be assigned a research code number. Data that are part of the eligibility checklist and contact information will be stored in the password-protected Clinical Trials Management Application (CTMA) database system at each site. The information linking the research code numbers to the patients' identities will be accessible only to the registry investigators and staff. No Protected Health Information or personal identifiers will be transferred between the University of Pittsburg and Fred Hutchinson Cancer Research Center.

6.2 Pre-Treatment Clinical Evaluation (within 2 weeks of treatment unless specified)

After a subject signs informed consent, the procedures listed below will be performed within two weeks prior to treatment. The schedule of events is summarized in **Section 15, Appendix C**

6.2.1 History, physical exam, and performance status

- History of primary disease and treatment
- Past medical history
- Performance status (ECOG and Karnofsky scale)

- Physical examination
- Concurrent medication
- Pre-existing symptoms and signs using CTCAE version 5.0 or equivalent

6.2.2 Assessment of history and active GVHD

- Past and current acute GVHD using CIBMTR grading system (Appendix A)
- Past and current chronic GVHD using NIH scoring system (Appendix B)
- GVHD prophylaxis
- Treatment of GVHD

6.2.3 Information on allogeneic stem cell transplantation

- Transplant related clinical information HLA typing (donor and recipient)
- Pre-transplant co-morbidity and HCT-comorbidity index (recipient, if available)
- Demographics: age, gender, race (donor and recipient)
- Type of stem cells, the dose of total nuclear cells, CD34 cells, and T cells in the graft (if available)
- Pre-transplant infection serology profiling (donor and recipient): CMV, EBV, Toxoplasma, and hepatitis
- Blood type and antibody screen (donor and recipient)
- HLA-alloantibody (donor and recipient, only if available)
- Conditioning regimen before the transplant
- Post-transplant treatment including chemotherapy, targeted kinase inhibitors, cell therapy
- History of infections
- History of transplant-related complications
- History of graft failure

6.2.4 Bone marrow aspirate and biopsy – This can be the same bone marrow sample as was used to assess eligibility or in the course of routine clinical care if done within two weeks of treatment initiation. A single sample collection consent form can be used prior to official enrollment for the study in order to obtain a research sample from this bone marrow aspirate in patients being considered for participation in this study within two weeks.

- Morphologic and flow cytometric assessment of the malignant blast percentage and evidence of dysplasia in subjects with MDS
- Chromosome analysis, if clinically indicated
- Minimal residual disease markers by any methodology
- BCR-ABL (CML, and Ph+ AML only), PML-RARα (APL only)
- Myeloid panel (AML or MDS-specific) next-generation sequencing if clinically available under the standard care.

6.2.5 Laboratory testing

- Pregnancy test (serum or urine) for women of child-bearing age

- CBC with differential, chemistry panel (Na, K, Cl, CO2, creatinine, glucose, urea nitrogen, phosphorus, magnesium, albumin, calcium, LDH, uric acid), hepatic (alkaline phosphatase, ALT, AST, and total bilirubin)
- STR chimerism of peripheral blood ($CD3^+$ and $CD33^+$ myeloid)

6.3 Stage 1: Safety evaluation of a fixed dose of IFN-γ

6.3.1 Clinical assessment before the first dose of IFN-γ

- Before the first dose of IFN-γ, the study team or patient's treating clinical team will take vital signs (temperature, blood pressure, and heart rate) and history of symptoms related to exclusion criteria for the first administration of IFN-γ, as listed below.

- The symptoms/signs which will disqualify the subjects from receiving the first dose of IFN-γ include
    - Flu-like symptoms within the past 48 hours
    - Fever >= 38.0 within the past 48 hours
    - Hypotension systolic BP < 90 mmHg
    - Diarrhea or vomiting within the past 48 hours if outside the patient's established pattern of GI recovery or GI side effects post-transplant
    - Other symptoms suggesting active acute infections within the past 48 hours
    - Finding consistent with GVHD that exceeds inclusion criteria

- Subjects deemed ineligible for the first IFN-γ due to infectious symptoms can be reassessed at a later time.

6.3.2 Dose adjustments of IFN-γ

- The initial dose of IFN-γ will be a 100 mcg fixed-dose three times per week.

- Each dose will be administered subcutaneously using a needle size as per institutional standard. Subsequent doses of IFN-γ will be administered to different locations in either arm, abdomen, or thigh.

- The first dose of IFN-γ will be administered by one of the clinical staff, nurses, or research staff at Hillman Cancer Center. At Seattle Cancer Care Alliance or the University of Washington Medical Center, IFN-γ will be administered by clinical nursing staff. At the first visit to the clinic, the patient will receive the self-injection training for the subsequent administration of IFN-γ at home. The self-injection training can be repeated at a subsequent visit if necessary. The IFN-γ injection diary will be recorded by the subject.

- Acetaminophen 650mg with or without prochlorperazine 5-10mg as pre-medication can be given before each dose of Actimmune at physician's discretion

- Schedule of IFN-γ and dose adjustment for cohort 1 (Figure 4)

1) The initial dose of IFN-γ

a. The first dose of IFN-γ 100 mcg will be administered on day 0 in the clinic.
b. The subject will continue self-injection of 100mcg, three times a week for two weeks (e.g., Monday, Wednesday, Friday or Tuesday, Thursday, Saturday)

2) 48 hours after the initial dose of IFN-γ (up to 1 week)
    a. Bone marrow aspirate (no biopsy) will be performed for morphology, flow cytometry, and a research sample. (Cytogenetics/molecular testing only needed if clinically indicated to follow MRD.)

3) Two weeks after the initial dose of IFN-γ (window: +/- 5 days)
    a. If 100 mcg per day three times a week is tolerated, the subject will continue IFN-γ for the next two weeks.
    b. If the subject is not tolerating at the dose of 100mcg per day three times a week for the first two weeks, decrease to 100 mcg two times a week for two weeks.

4) Four weeks after the initial dose of IFN-γ
    a. Subjects tolerating 100 mcg three times a week can proceed to DLI if eligible.
    b. If the subject is not tolerating a given dose, it can be decreased to 100mcg two times a week for another two weeks (+/- 5 days). If tolerated, the patient would then be eligible to proceed to the DLI phase.
    c. If 100 mcg two times per week is not tolerated, IFN-γ will be discontinued, and the subject will be withdrawn from further on-study treatment. The subject may still proceed to DLI off study.

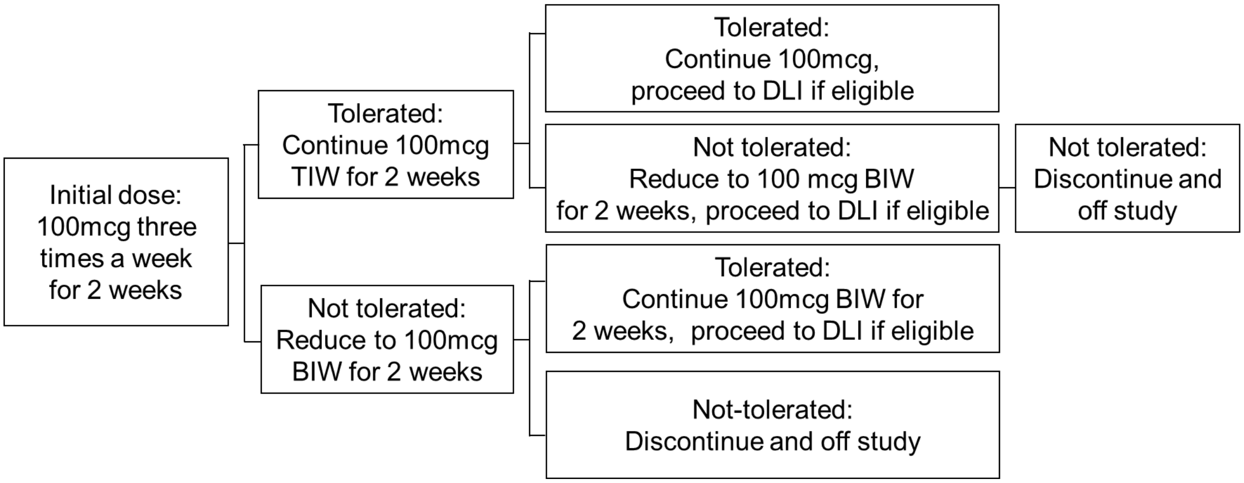

Figure 4. The dose adjustment plan for IFN-γ for cohort 1.

- Schedule of IFN-γ and dose adjustment for cohort 2 (Figure 5)

1) The initial dose of IFN-γ
    a. The first dose of IFN-γ 100 mcg will be administered on day 0 in the clinic.
    b. The subject will continue self-injection of 100mcg, three times a week for two weeks (e.g., Monday, Wednesday, Friday or Tuesday, Thursday, Saturday)

2) 48 hours after the initial dose of IFN-γ (up to 1 week)
    a. Bone marrow aspirate (no biopsy) will be performed for morphology, flow cytometry, and a research sample. (Cytogenetics/molecular testing only needed if clinically indicated to follow MRD.)

3) One week after the initial dose of IFN-γ (window: - 3 days to + 7 days)
    a. Subjects tolerating 100 mcg three times a week can proceed to DLI if eligible.
    b. If the subject is not tolerating a given dose, it can be decreased to 100mcg two times a week for one week (+/- 5 days). If tolerated, the patient would then be eligible to proceed to the DLI phase.
    c. If 100 mcg two times per week is not tolerated, IFN-γ will be discontinued, and the subject will be withdrawn from further on-study treatment. The subject may still proceed to DLI off study.

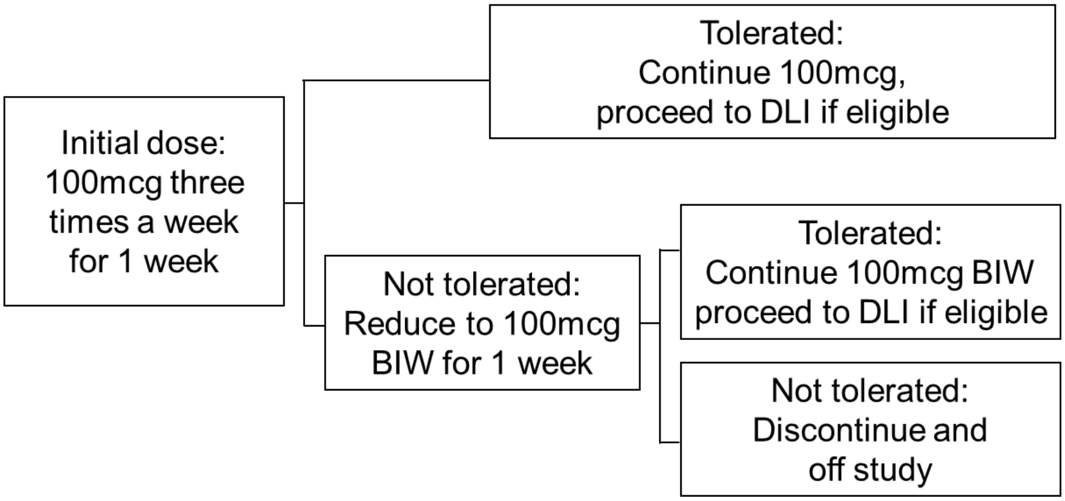

Figure 5. The dose adjustment plan for IFN-γ for cohort 2.

6.4 Stage 2: Donor Lymphocyte Infusion

6.4.1 Donor lymphocyte infusion

6.4.1.1 Donor

- Donors are assessed and selected as per institutional guidelines.
- Donors are restricted to the original sibling or unrelated donor

6.4.1.2 DLI product

- DLI will be prepared under the standard of care per institutional guideline.
- Cryopreserved GCSF-mobilized PBMCs collected at the time of transplant can be thawed and infused per institutional guideline and are considered as "DLI."
- If donor leukocytes are newly collected from the original donor, the donor will undergo the pre-apheresis assessment and apheresis for collection per institutional or the National Marrow Donor Program guideline. The collected cells will be stored in aliquots and cryopreserved for subsequent infusion.

6.4.1.3 Dose and schedule of DLI

- In the absence of active grade II-IV GVHD or de novo grade III-IV, or progressive chronic GVHD requiring new systemic treatment or increased doses of current systemic treatment, the subject will receive the DLI at doses as per Table 1.

- Based on the response assessment (improvement in donor chimerism, change in malignant cells in blood or bone marrow) or toxicity, principally GVHD, patients may proceed to a second or third DLI (Table 3).

Table 3. Recommended dose escalation every 4 weeks of donor lymphocyte infusions. The actual dose of DLI will be determined by the subject's treating physician.

DLI number	DLI dose ($CD3^+$ T cells/kg recipient weight ± 20%)	
	HLA matched sib	HLA matched unrelated
DLI #1 (starting dose)	$5x\ 10^6$	$5x\ 10^6$
DLI #2	$1x\ 10^7$	$1x\ 10^7$
DLI #3	Up to 1×10^8 per treating MD decision	Up to $5x\ 10^7$ per treating MD decision

6.4.2 Dose and schedule of IFN-γ during the donor lymphocyte infusion stage

6.4.2.1 For the subjects for whom donor leukocytes are available (cohort 1)

- After the first dose of DLI, the same dose of IFN-γ as used pre-DLI for that patient will be continued three times a week for four weeks. If the subject develops intolerance to IFN-γ during this period, we will reduce the frequency of IFN-γ to once weekly.

- Four weeks after the first dose of DLI, the frequency of IFN-γ will be tapered to once weekly, continued until the next DLI.

- A minimum of four weeks after the first dose of DLI, the subject will receive the second DLI, in the absence of active grade II-IV GVHD or de novo grade III-IV, or progressive chronic GVHD requiring new systemic treatment or increased doses of current systemic treatment per treating physician's determination. IFN-γ will be continued at the same dose once weekly. There is an optional bone marrow aspirate if clinically indicated prior to the second DLI per the treating physician, and a research sample can be collected at that time.

- A minimum of 4 weeks after the second DLI, the subject will receive the third dose of DLI, in the absence of active grade II-IV GVHD or de novo grade III-IV, or progressive chronic GVHD defined by a requirement for new systemic treatment or increased doses of current systemic treatment. IFN-γ will be continued at the same dose once weekly for

four more weeks. There is an optional bone marrow aspirate if clinically indicated before the third DLI, and a research sample may also be collected at that time.

- IFN-γ will be continued once weekly until four weeks of the last dose of DLI or 12 weeks after the first dose of IFN-γ whichever occurs later. If the subject develops intolerance to IFN-γ during this period, we will discontinue IFN-γ

- At the end IFN-γ treatment, the bone marrow aspirate is required ideally within seven days after the last dose of IFN-γ

6.4.2.2 For the subjects for whom donor leukocytes are available (cohort 2)

- After the first dose of DLI, the same dose of IFN-γ as used pre-DLI for that patient will be continued three times a week for four weeks. If the subject develops intolerance to IFN-γ during this period, we will reduce the frequency of IFN-γ to once weekly.

- A minimum of four weeks after the first dose of DLI, the subject will receive the second DLI, in the absence of active grade II-IV GVHD or de novo grade III-IV, or progressive chronic GVHD requiring new systemic treatment or increased doses of current systemic treatment per treating physician's determination. IFN-γ will be continued at the same dose three times weekly. There is an optional bone marrow aspirate if clinically indicated prior to the second DLI per the treating physician, and a research sample can be collected at that time.

- 8 weeks after the first dose of IFN-γ (or approximately 7 weeks after the first dose of DLI), the frequency of IFN-γ will be tapered to once weekly, continued until the next DLI.

- A minimum of 4 weeks after the second DLI, the subject will receive the third dose of DLI, in the absence of active grade II-IV GVHD or de novo grade III-IV, or progressive chronic GVHD defined by a requirement for new systemic treatment or increased doses of current systemic treatment. IFN-γ will be continued at the same dose once weekly for three more weeks. There is an optional bone marrow aspirate if clinically indicated before the third DLI, and a research sample may also be collected at that time.

- IFN-γ will be continued once weekly until four weeks of the last dose of DLI or 12 weeks after the first dose of IFN-γ whichever occurs later. If the subject develops intolerance to IFN-γ during this period, we will discontinue IFN-γ

- At the end IFN-γ treatment, the bone marrow aspirate is required ideally within seven days after the last dose of IFN-γ

6.4.2.3 For subjects for whom donor leukocytes are not available after a minimum of 4 weeks of treatment in the stage 1 phase

- In the absence of active grade II-IV GVHD or de novo grade III-IV, or progression of cGVHD requiring new systemic treatment or increased doses of current systemic treatment, the subject will continue the same dose of IFN-γ he or she is currently tolerating three times a week for two more weeks

- Then, IFN-γ will be reduced to once weekly to complete a total of 12 weeks of therapy measured from the time of the first dose of IFN-γ.

6.5 Protocol Visit, Monitoring, and Biospecimen Collection

6.5.1 Protocol Visits for Cohort 1

6.5.1.1 Pre-treatment bone marrow aspirates for research (within two weeks before <u>IFN-γ administration)</u>

- **Bone marrow aspirates** (no biopsy) will be performed for morphology, flow cytometry, and research sample (10mL) if the bone marrow research sample is not collected within two weeks before the first dose of IFN-γ. The bone marrow aspirate for research can be performed concurrently with protocol visit #1, the first dose of IFN-γ.

6.5.1.2 Protocol visit #1: Stage 1, the first dose of IFN-γ

- Pre-treatment assessment (bone marrow aspirates, vital signs, and clinical assessment) as described in 6.3.1 will be performed before IFN-γ administration.
- **Bone marrow aspirates** (no biopsy) will be performed for morphology, flow cytometry, and research sample (10mL) if the bone marrow research sample is not collected within two weeks before the first dose of IFN-γ.
- <u>Research sample collection: A bone marrow aspirate with a goal of 10 mL collected (in 10ml green top heparin tube x 1) and 30 mL of peripheral blood (10 mL green top heparin tube x 3 tubes) will be obtained as a baseline sample before IFN-γ administration.</u>
- First dose of IFN-γ will be administered after the safety assessment.
- As described in 6.3.1., the patient will receive the training for self-injection of IFN-γ
- Safety monitoring: Subjects will be assessed for an acute reaction to the IFN-γ or complication of biospecimen collection. Subjects will stay in the clinic for at least 1 hour after IFN-γ administration and be assessed by a physician, physician assistant, advanced practice nurse or nurse prior to discharge from the clinic.

6.5.1.3 Protocol visit #2: Stage 1, minimally 48 hours (up to one week) after the first dose of IFN-γ: Safety and biological assessment of IFN-γ, bone marrow aspiration for research

- Clinical safety monitoring: Any reaction or symptoms related to the prior doses of IFN-γ and will be assessed specifically focusing on active grade II-IV GVHD or de novo grade III-IV, or progressive chronic GVHD
- **Bone marrow aspirates** (no biopsy) will be performed for morphology, flow cytometry, and research sample (10mL).
- <u>Research sample collection: 10mL of bone marrow (10ml green top heparin tube), 30 mL of peripheral blood (10 mL green top heparin tube x 3 tubes) will be collected.</u>
- The subsequent doses of IFN-γ will be administered after the safety assessment.

6.5.1.4 Protocol visit #3: Stage 1, Day +14 (± 5 days) after the first dose of IFN-γ: Safety assessment of IFN-γ

- Clinical safety monitoring: Any reaction or symptoms related to the prior doses of IFN-γ and will be assessed specifically focusing on active grade II-IV GVHD or de novo grade III-IV, or progressive chronic GVHD
- Laboratory monitoring: CBC with differential, chemistry panel (Na, K, Cl, CO2, creatinine, glucose, urea nitrogen, phosphorus, magnesium, albumin, calcium, LDH, uric acid), hepatic (alkaline phosphatase, ALT, AST, and total bilirubin) panel
- The subsequent doses of IFN-γ will be administered after safety assessment.

6.5.1.5 Protocol visit #4: End of stage 1 and pre-treatment evaluation for stage 2 (donor lymphocyte infusion), day +28 (± 5 days) after the first dose of IFN-γ or two weeks (± 5 days) after a stable dose of IFN-γ:

- Clinical safety monitoring: Any reaction or symptoms related to the prior doses of IFN-γ and will be assessed specifically focusing on active grade II-IV GVHD or de novo grade III-IV, or progressive chronic GVHD
- Laboratory monitoring: CBC with differential, chemistry panel (Na, K, Cl, CO2, creatinine, glucose, urea nitrogen, phosphorus, magnesium, albumin, calcium, LDH, uric acid), hepatic (alkaline phosphatase, ALT, AST, and total bilirubin) panel
- STR chimerism of bone marrow (if collected) and peripheral blood (whole blood, or CD3 and myeloid)
- **Bone marrow aspirates** (no biopsy) will be performed for morphology, flow cytometry, and research sample (10mL). Cytogenetics/molecular testing only needed if clinically indicated to follow MRD.
- Research sample collection: 10mL of bone marrow (10mL green top heparin tube), 30 mL of peripheral blood (10 mL green top heparin tube x 3 tubes) will be collected.
- The subsequent doses of IFN-γ will be administered after the safety assessment.
- If DLI is available, the first dose of DLI will be infused within two weeks from this visit.

6.5.1.6 Protocol visit #5: Stage 2 first safety assessment, 14 days (+/- 5 days) after the first dose of DLI, or two weeks (+/- 5 days) after the end visit of the first stage for non-DLI subjects.

- Clinical safety monitoring: Any reaction or symptoms related to the IFN-γ or DLI will be assessed, specifically focusing on active grade II-IV GVHD or de novo grade III-IV, or progressive chronic GVHD
- Laboratory monitoring: CBC with differential, chemistry panel (Na, K, Cl, CO2, creatinine, glucose, urea nitrogen, phosphorus, magnesium, albumin, calcium, LDH, uric acid), hepatic (alkaline phosphatase, ALT, AST, and total bilirubin) panel
- The subsequent frequency IFN-γ will be reduced to once-weekly dosing for four more weeks.

6.5.1.7 Protocol visit #6: Stage 2 follow up safety assessment, 28 days (+/- 5 days) after the first dose of DLI, or 28 days (+/- 5 days) after the end visit of the first stage for non-DLI subjects

- Clinical safety monitoring: Any reaction or symptoms related to the IFN-γ or DLI will be assessed, specifically focusing on active grade II-IV GVHD or de novo grade III-IV, or progressive chronic GVHD
- Laboratory monitoring: CBC with differential, chemistry panel (Na, K, Cl, CO2, creatinine, glucose, urea nitrogen, phosphorus, magnesium, albumin, calcium, LDH, uric acid), hepatic (alkaline phosphatase, ALT, AST, and total bilirubin) panel
- Research sample collection: 30 mL of peripheral blood (10 mL green top heparin tube x 3 tubes) will be collected.
- Optional bone marrow aspirates (no biopsy) will be performed for morphology, flow cytometry, and research sample (10ml) if clinically indicated. Cytogenetics/molecular testing only needed if clinically indicated to follow MRD if clinically indicated.
- If the second or third dose of DLI is available, the DLI will be infused at minimal 4 weeks of interval in the absence of active grade II-IV GVHD or de novo grade III-IV, or progressive chronic GVHD requiring new treatment or increased doses of current treatment per treating physician's determination.
- There is an optional bone marrow aspirate if clinically indicated before the second or the third DLI per the treating physician, and research sample (10mL in green top heparin tube) will be collected.

6.5.1.8 Protocol visit #7: Stage 2, end of IFN-γ therapy assessment

- At the 28 days (+/- 5 days) after the last dose of DLI, or eight weeks (+/- 5 days) after the end visit of the first stage for non-DLI subjects
- Clinical safety monitoring: Any reaction or symptoms related to the IFN-γ or DLI will be assessed, specifically focusing on active grade II-IV GVHD or de novo grade III-IV, or progressive chronic GVHD
- Laboratory monitoring: CBC with differential, chemistry panel (Na, K, Cl, CO2, creatinine, glucose, urea nitrogen, phosphorus, magnesium, albumin, calcium, LDH, uric acid), hepatic (alkaline phosphatase, ALT, AST, and total bilirubin) panel
- STR chimerism of bone marrow (if available) and peripheral blood (CD3 and myeloid)
- **Bone marrow aspirates** (no biopsy) will be performed for morphology, flow cytometry, and research sample (10mL). Cytogenetics/molecular testing only needed if clinically indicated to follow MRD within 3 days after the last dose of IFN-γ
- Research sample collection: 10mL of bone marrow (10ml green top heparin tube), 30 mL of peripheral blood (10 mL green top heparin tube x 3 tubes) will be collected.
- The date of the last dose of IFN-γ will be recorded.

6.5.1.9 Protocol visit #8: 28 days (+/- 5 days) safety assessment after the last dose of IFN-γ therapy (Off treatment assessment)

- Clinical safety monitoring: Any reaction or symptoms related to the IFN-γ or DLI will be assessed, specifically focusing on active grade II-IV GVHD or de novo grade III-IV, or progressive chronic GVHD. Monitoring of adverse events annotated to IFN-γ therapy will be completed on this off-treatment assessment visit
- Laboratory monitoring: CBC with differential, chemistry panel (Na, K, Cl, CO2,

creatinine, glucose, urea nitrogen, phosphorus, magnesium, albumin, calcium, LDH, uric acid), hepatic (alkaline phosphatase, ALT, AST, and total bilirubin) panel

- Research sample collection: 30 mL of peripheral blood (10 mL green top heparin tube x 3 tubes) will be collected.

6.5.1.10 Protocol visit #9: Off study assessment: 6 months (+/- 14 days) after the first dose of IFN-γ

- Clinical outcome monitoring: Any reaction or symptoms related to the IFN-γ or DLI will be assessed, specifically focusing on active grade II-IV GVHD or de novo grade III-IV, or progressive chronic GVHD. Other clinical outcomes, including the latest disease status, will be recorded.
- STR chimerism of peripheral blood (CD3 and myeloid)
- Optional bone marrow aspirates (no biopsy) will be performed for morphology, flow cytometry, and research sample (10ml). Cytogenetics/molecular testing only needed if clinically indicated to follow MRD if clinically indicated.
- Research sample collection: 10ml of bone marrow (green top heparin tube) if collected, 30 mL of peripheral blood (10 mL green top heparin tube x 3 tubes) will be collected.

6.5.2 Protocol Visits for Cohort 2

6.5.2.1 Pre-treatment bone marrow aspirates for research (within two weeks before IFN-γ administration)

- **Bone marrow aspirates** (no biopsy) will be performed for morphology, flow cytometry, and research sample (10mL) if the bone marrow research sample is not collected within two weeks before the first dose of IFN-γ. The bone marrow aspirate for research can be performed concurrently with protocol visit #1, the first dose of IFN-γ.

6.5.2.2 Protocol visit #1: Stage 1, the first dose of IFN-γ

- Pre-treatment assessment (bone marrow aspirates, vital signs, and clinical assessment) as described in 6.3.1 will be performed before IFN-γ administration.
- **Bone marrow aspirates** (no biopsy) will be performed for morphology, flow cytometry, and research sample (10mL) if the bone marrow research sample is not collected within two weeks before the first dose of IFN-γ.
- Research sample collection: A bone marrow aspirate with a goal of 10 mL collected (in 10ml green top heparin tube x 1) and 30 mL of peripheral blood (10 mL green top heparin tube x 3 tubes) will be obtained as a baseline sample before IFN-γ administration.
- First dose of IFN-γ will be administered after the safety assessment.
- As described in 6.3.1., the patient will receive the training for self-injection of IFN-γ
- Safety monitoring: Subjects will be assessed for an acute reaction to the IFN-γ or complication of biospecimen collection. Subjects will stay in the clinic for at least 1 hour after IFN-γ administration and be assessed by a physician, physician assistant, advanced practice nurse or nurse prior to discharge from the clinic.

6.5.2.3 Protocol visit #2: Stage 1, minimally 48 hours (up to one week) after the first dose of IFN-γ: Safety and biological assessment of IFN-γ, bone marrow aspiration for research

- Clinical safety monitoring: Any reaction or symptoms related to the prior doses of IFN-γ and will be assessed specifically focusing on active grade II-IV GVHD or de novo grade III-IV, or progressive chronic GVHD
- **Bone marrow aspirates** (no biopsy) will be performed for morphology, flow cytometry, and research sample (10mL).
- Research sample collection: 10mL of bone marrow (10ml green top heparin tube), 30 mL of peripheral blood (10 mL green top heparin tube x 3 tubes) will be collected.
- The subsequent doses of IFN-γ will be administered after the safety assessment.

6.5.2.4 Protocol visit #3: End of stage 1 and pre-treatment evaluation for stage 2 (donor lymphocyte infusion), Day +7 (-3 days to +5 days) after the first dose of IFN-γ: Safety assessment of IFN-γ

- Clinical safety monitoring: Any reaction or symptoms related to the prior doses of IFN-γ and will be assessed specifically focusing on active grade II-IV GVHD or de novo grade III-IV, or progressive chronic GVHD
- Laboratory monitoring: CBC with differential, chemistry panel (Na, K, Cl, CO2, creatinine, glucose, urea nitrogen, phosphorus, magnesium, albumin, calcium, LDH, uric acid), hepatic (alkaline phosphatase, ALT, AST, and total bilirubin) panel
- The subsequent doses of IFN-γ will be administered after the safety assessment.
- If DLI is available, the first dose of DLI will be infused within two weeks from this visit.

6.5.2.5 Protocol visit #4: Stage 2 first safety assessment, 21 days (+/- 5 days) after the first dose of IFN-γ, or 14 days (+/- 5 days) after the first dose of DLI.

- Clinical safety monitoring: Any reaction or symptoms related to the IFN-γ or DLI will be assessed, specifically focusing on active grade II-IV GVHD or de novo grade III-IV, or progressive chronic GVHD
- Laboratory monitoring: CBC with differential, chemistry panel (Na, K, Cl, CO2, creatinine, glucose, urea nitrogen, phosphorus, magnesium, albumin, calcium, LDH, uric acid), hepatic (alkaline phosphatase, ALT, AST, and total bilirubin) panel
- The subsequent frequency IFN-γ will be reduced to once-weekly dosing for four more weeks.

6.5.2.6 Protocol visit #5: Stage 2 follow up safety assessment, 35 days (+/- 5 days) after the first dose of IFN-γ, or 28 days (+/- 5 days) after the first dose of DLI

- Clinical safety monitoring: Any reaction or symptoms related to the IFN-γ or DLI will be assessed, specifically focusing on active grade II-IV GVHD or de novo grade III-IV, or progressive chronic GVHD
- Laboratory monitoring: CBC with differential, chemistry panel (Na, K, Cl, CO2, creatinine, glucose, urea nitrogen, phosphorus, magnesium, albumin, calcium, LDH, uric acid), hepatic (alkaline phosphatase, ALT, AST, and total bilirubin) panel
- STR chimerism of bone marrow (if collected) and peripheral blood (whole blood, or CD3 and myeloid)
- Research sample collection: 30 mL of peripheral blood (10 mL green top heparin tube x

3 tubes) will be collected.

- Optional bone marrow aspirates (no biopsy) will be performed for morphology, flow cytometry, and research sample (10ml) if clinically indicated. Cytogenetics/molecular testing only needed if clinically indicated to follow MRD if clinically indicated.
- If the second or third dose of DLI is available, the DLI will be infused at minimal 4 weeks of interval in the absence of active grade II-IV GVHD or de novo grade III-IV, or progressive chronic GVHD requiring new treatment or increased doses of current treatment per treating physician's determination.
- There is an optional bone marrow aspirate if clinically indicated before the second or the third DLI per the treating physician, and research sample (10mL in green top heparin tube) will be collected.

6.5.2.7 Protocol visit #6: Stage 2 follow up safety assessment, 56 days (+/- 7 days) after the first dose of IFN-γ (= approximately 49 days +/- 7 days after the first DLI or 21 days +/- 7 days after the second DLI)

- Clinical safety monitoring: Any reaction or symptoms related to the IFN-γ or DLI will be assessed, specifically focusing on active grade II-IV GVHD or de novo grade III-IV, or progressive chronic GVHD
- Laboratory monitoring: CBC with differential, chemistry panel (Na, K, Cl, CO2, creatinine, glucose, urea nitrogen, phosphorus, magnesium, albumin, calcium, LDH, uric acid), hepatic (alkaline phosphatase, ALT, AST, and total bilirubin) panel
- Research sample collection: 30 mL of peripheral blood (10 mL green top heparin tube x 3 tubes) will be collected.
- Optional bone marrow aspirates (no biopsy) will be performed for morphology, flow cytometry, and research sample (10ml) if clinically indicated. Cytogenetics/molecular testing only needed if clinically indicated to follow MRD if clinically indicated.
- If the third dose of DLI is available, the DLI will be infused at minimal 4 weeks of interval in the absence of active grade II-IV GVHD or de novo grade III-IV, or progressive chronic GVHD requiring new treatment or increased doses of current treatment per treating physician's determination.
- There is an optional bone marrow aspirate if clinically indicated before the third DLI per the treating physician, and research sample (10mL in green top heparin tube) will be collected.

6.5.2.8 Protocol visit #7: Stage 2, end of IFN-γ therapy assessment

- At the 12 weeks (+/- 2 weeks) after the first dose of IFN-γ
- Clinical safety monitoring: Any reaction or symptoms related to the IFN-γ or DLI will be assessed, specifically focusing on active grade II-IV GVHD or de novo grade III-IV, or progressive chronic GVHD
- Laboratory monitoring: CBC with differential, chemistry panel (Na, K, Cl, CO2, creatinine, glucose, urea nitrogen, phosphorus, magnesium, albumin, calcium, LDH, uric acid), hepatic (alkaline phosphatase, ALT, AST, and total bilirubin) panel
- STR chimerism of bone marrow (if available) and peripheral blood (CD3 and myeloid)
- **Bone marrow aspirates** will be performed for morphology, flow cytometry, and research sample (10mL). Cytogenetics/molecular testing only needed if clinically indicated to follow MRD within 2 weeks after the last dose of IFN-γ

- Research sample collection: 10mL of bone marrow (10ml green top heparin tube), 30 mL of peripheral blood (10 mL green top heparin tube x 3 tubes) will be collected.
- The date of the last dose of IFN-γ will be recorded.

6.5.2.9 Protocol visit #8: 28 days (+/- 2 weeks) safety assessment after the last dose of IFN-γ therapy (Off treatment assessment)

- Clinical safety monitoring: Any reaction or symptoms related to the IFN-γ or DLI will be assessed, specifically focusing on active grade II-IV GVHD or de novo grade III-IV, or progressive chronic GVHD. Monitoring of adverse events annotated to IFN-γ therapy will be completed on this off-treatment assessment visit
- Laboratory monitoring: CBC with differential, chemistry panel (Na, K, Cl, CO2, creatinine, glucose, urea nitrogen, phosphorus, magnesium, albumin, calcium, LDH, uric acid), hepatic (alkaline phosphatase, ALT, AST, and total bilirubin) panel
- Research sample collection: 30 mL of peripheral blood (10 mL green top heparin tube x 3 tubes) will be collected.

6.5.2.10 Protocol visit #9: Off study assessment: 6 months (+/- 4 weeks) after the first dose of IFN-γ

- Clinical outcome monitoring: Any reaction or symptoms related to the IFN-γ or DLI will be assessed, specifically focusing on active grade II-IV GVHD or de novo grade III-IV, or progressive chronic GVHD. Other clinical outcomes, including the latest disease status, will be recorded.
- STR chimerism of peripheral blood (CD3 and myeloid)
- Optional bone marrow aspirates (no biopsy) will be performed for morphology, flow cytometry, and research sample (10ml). Cytogenetics/molecular testing only needed if clinically indicated to follow MRD if clinically indicated.
- Research sample collection: 10ml of bone marrow (green top heparin tube) if collected, 30 mL of peripheral blood (10 mL green top heparin tube x 3 tubes) will be collected.

6.6 Management of Complications

6.6.1 Criteria for Dose Reduction of IFN-γ

The dose of IFN-γ will be reduced when the subject is considered to have an intolerance to IFN-γ defined by the following criteria.

6.6.1.1 Graft versus host disease

- De novo grade II GVHD
- Progressive chronic GVHD requiring new systemic treatment or increased doses of current systemic treatment
- Any grade of GVHD which requires increased doses of current treatment per treating physician's determination

6.6.1.2 Other organ diseases

- New onset of seizure which is not medically controlled

- New onset of cardiac ischemia, congestive heart failure or arrhythmia which is not medically controlled
- New onset of grade 3 or greater AST or ALT elevation
- Development of acute kidney failure with an estimated GFR < 30 mL/min

6.6.1.3 Intolerance per the subject

- If the subject develops intolerance due to the expected adverse events (listed in Section 9.2.2), the dose may be reduced per treating physician or investigator discretion. If the symptoms are eventually attributed to other cause or resolve, then the dose may be increased again, per treating physician or investigator discretion.

6.7 Criteria for Discontinuation of IFN-γ

Specific conditions that will require discontinuation of IFN-γ are as follows:

- Pregnancy
- New-onset grade 3 or 4 acute GVHD
- Progression of disease which requires immediate cytoreduction chemotherapy or other therapy per treating physician's determination
- Immediate hypersensitivity reaction associated with a study injection
- Intercurrent CTCAE grade 3 or 4 illness or organ dysfunction (other than cytopenias and electrolyte abnormalities) that does not resolve to grade <= 1 within two weeks
- The study site PI or treating physician assesses that it is not in the best interest of the subject to continue on the study.
- The dose of IFN-γ can be discontinued anytime based on the clinical status of the subject at the treating physician's discretion. If IFN-γ is not given for six doses, the subject will be withdrawn from further on-study treatment.

6.8 Management of hypersensitivity reactions to IFN-γ

If a systemic hypersensitivity reaction occurs, IFN-γ should be discontinued immediately and appropriate medical therapy should be instituted. For a severe hypersensitivity reaction, intramuscular epinephrine and intravenous antihistamines and systemic steroid with fluid resuscitation should be immediately initiated under appropriate clinical monitoring.

6.9 Management of Other Complications

6.9.1 Cytopenia, GVHD, and infection: Management of cytopenia, GVHD, and infection will be per the institutional Standard Practice Guidelines. These complications could be due in part or in full to IFN-γ, DLI, or the side effects of the transplant itself.

6.9.2 Flu-like symptoms

Flu-like symptoms such as fever, chills, rash, headache, myalgia, tachycardia, shortness of breath, nausea, and diarrhea can occur especially within the first week of IFN-γ treatment. Treatment for flu-like symptoms will be primary supportive (i.e., empiric antibiotics, IV fluid, antipyretic, anti-emesis, anti-diarrheal after ruling out infectious causes). Some of "flu-like" symptoms may be minimized by bedtime administration of IFN-γ. Prophylactic acetaminophen may also be used to ameliorate these effects.

6.9.3 Organ failure

- Neurologic: Decreased mental status, gait disturbance, and dizziness have been observed, particularly in patients receiving IFN doses greater than 250 mcg/m2/day (greater than 10 times the weekly maximal dose in this study). Most of these abnormalities were reversible within a few days upon dose reduction or discontinuation of therapy. We will clinically monitor the patients with seizure disorders or compromised central nervous system function.

- Cardiopulmonary system: Patients with pre-existing cardiopulmonary conditions, including ischemia, congestive heart failure, or arrhythmia, will be clinically monitored for signs/symptoms of exacerbation. <u>Some of "flu-like" symptoms may be minimized by bedtime administration of IFN-γ.</u> <u>Acetaminophen may also be used to ameliorate these effects.</u>

- Liver: Repeated administration of IFN-γ to patients with advanced hepatic disease may result in accumulation of interferon gamma-1b. Elevations of aspartate transaminase (AST) and/or alanine transaminase (ALT) (up to 25-fold) have been observed during IFN-γ therapy. The transaminase elevations were reversible with reduction in dosage or interruption of IFN-γ treatment. If severe hepatic enzyme elevations develop, IFN-γ dosage will be modified according to the does reduction criteria described in section 6.

- Renal: Renal toxicity has been reported in patients receiving IFN-γ; if new or progressive renal insufficiency occurs, that cannot be attributed to another cause (for example, a nephrotoxic medication), the dosage will be modified according to the does reduction criteria described in section 6.6.1. Nephrology consultation is recommended in this case.

6.10 Collection of Biospecimens

6.10.1 Saliva collection (optional)

The subject will be provided with a saliva collection kit and instructed to collect the saliva according to manufacturing instruction. Saliva collection can be repeated one more time if the amount of extracted DNA is not adequate for subsequent sequencing. Collection of saliva can occur at any time during the study.

6.10.2 Bone marrow aspirates

- An extra volume (goals of a minimum of 5 mL, up to 10 mL) of bone marrow aspirate will be collected for research at the specific time point, as described in section 6.5.

6.10.3 Peripheral blood collection

- Peripheral blood will be collected from the subjects, as described in section 6.5. The volumes of blood collection 30 mL of peripheral blood per time point. The research blood draw will not be performed when the hemoglobin level is less than 7.5 g/dL.

6.11 Biospecimen Management and Research Data Collection

6.11.1 De-identification of the biospecimen: each biospecimen will be assigned a de-identified accession number according to the standard operating procedure at Hillman Cancer Center Research Pavilion Lab 1.33.

6.11.2 Sample processing and storage

- Processing of saliva, peripheral blood and bone marrow:

  De-identified samples will be processed and cryopreserved as plasma and/or mononuclear cells at:

  Hillman Cancer Center
  Research Facility
  Lab 1.33
  5115 Centre Avenue
  Pittsburgh PA 15213

  Investigator phone number: 412-779-8616

  Investigator e-mail address: itos3@upmc.edu

- Location and storage of biospecimens:

  Extracted coded nucleotide samples will be stored at UPMC Genome Center Lab or equivalent for a limited period according to their standard procedure. Other biospecimens will be stored in Lab 1.33 Hillman Cancer Center and Laboratory of Dr. Warren Shlomchik at 15th floor of Biomedical Science Tower

7. LABORATORY RESEARCH STUDIES

7.1 Response of malignant blasts to IFN-γ

Using flow cytometry, we plan to measure the IFN-γ response of malignant blasts through phosphorylated STAT1 (pSTAT1), a canonical pathway of IFN-γ receptor (IFN-γR) and surface expressions of other IFN-γ inducible molecules such as HLA-class I, HLA-class II, ICAM-1. We will also perform single-cell RNA sequencing (sc-RNAseq), or bulk RNA sequencing (RNAseq) of malignant blasts pre and post in vivo IFN-γ therapy, to identify IFN-γ-induced transcriptional changes.

7.2 Cellular and humoral immune response to IFN-γ

We also plan to analyze the immune response to IFN-γ by analyzing T-cell subset with flow

phenotype using spectral analyzer (Cytek™ Aurora) which includes markers of memory subset (CD45RO, CCR7), regulatory T cells (CD25hi CD127lowFoxP3+), activation and exhaustion (PD1, LAG3, Tim3, and TIGIT). We will measure plasma IFN-γ and related cytokines (IP-10) using a multiplex ELISA panel. Antigen-specific T cells against infectious pathogens (i.e., CMV or EBV) or minor histocompatibility antigens (miHAs, such as HA-1) may also be assessed.

8. STATISTICAL CONSIDERATIONS

8.1 Study Endpoint

8.1.1 Primary safety endpoint:

To evaluate the adverse events of IFN-γ in relapsed patients after alloSCT, per CTCAE v5.0

To evaluate theadverse events of IFN-γ in relapsed patients after alloSCT with concurrent use of donor lymphocyte infusion, per CTCAE v5.0

8.1.1.1 Analysis plan

Adverse events will be summarized via descriptive statistics. The maximum grade of toxicity for each category of interest will be recorded for each patient and the summary results will be tabulated by category and grade.

The following data will be reported

- Incidence of graft-versus-host disease (GVHD) progression
- Incidence of de novo GVHD
- Incidence of serious adverse events (SAE) or grade 3 or 4 unexpected, suspected adverse reaction defined in section 9.1

8.1.2 Primary biological endpoint:

Upregulation of key targets including HLA-ABC and -DR/DQ, ICAM-1 and generation of phosphorylated STAT1 (pSTAT1) in bone marrow malignant blasts post-IFN-γ treatment, measured by the changes in mean florescent intensity and percentage of positive cells by flow cytometry

8.1.2.1 Analysis plan

Biological parameter measurements and outcomes will be presented via descriptive statistics, table or figure.

8.2 Sample Size

8.2.1 Sample Size:

Our goal is to have at least three patients complete intra-subject dose escalation and receive at

least one dose of DLI. We anticipate enrolling 6 total patients and likely no more than 9 as some patients may not be able to complete the study due to rapid disease progression or the unavailability of DLI.

For cohort 2, we aim to enroll at least three patients receiving IFN-γ and at least one dose of DLI. We anticipate enrolling up to 6 patients as some patients may not be able to complete the study due to rapid disease progression.

8.2.2 Accrual Rate

We plan to accrue six to nine subjects during the first six months and complete the study in one year.

8.3 Off Study Criteria

8.3.1 Completion of study

Following the completion of IFN-γ and off treatment monitoring period, the subjects will have completed protocol participation and will be considered off study at the off-protocol visit, defined in section 6.5

8.3.2 Criteria for removal of a subject from the study

Subjects may be removed from the study, as described below. Subjects that are removed will have a final "off study" visit. If such subjects had bone marrow aspirates before and after IFN-γ (i.e., Protocol Visit #2), data from those aspirates will be included in assessing the biological endpoint.

- The subject does not receive at least 2 doses of IFN-γ per week due to noncompliance.
- The subject develops a medical condition that is a contraindication to IFN-γ as described in Section 6.7, Criteria for Discontinuation of IFN-γ, without having already received at least 4 weeks of IFN-γ therapy.
- The subject becomes pregnant.
- The study PI assesses that it is not in the best interests of the subject to continue participation in the study or that the subject is not compliant with the investigation.

8.3.3 Subject withdrawal

Subjects may withdraw from the protocol at any time. Subjects may withdraw permission for the use of unprocessed specimens, at which time those samples will be destroyed. This request must be made in writing, addressed to the PI. Data regarding DNA sequences will be unable to be destroyed at the request of the subject. Any sample, portion of a sample or product derived from that sample that has already been used in research, will not be able to be retrieved and destroyed.

8.4 Criteria for Study Suspension

8.4.1 The study will be suspended if there is any death within four weeks after the first dose of IFN-γ, which is probably or definitely attributed to IFN-γ, by the treating physician, site PI or study PI. IRB review would follow. Subjects already on IFN-γ would stop their injections.

8.4.2 Criteria for study resumption after a safety pause

The IFN-γ injection and enrollments of new subjects would resume only if a review of the adverse events that caused the pause resulted in an IRB recommendation to permit further study injections and study enrollments.

The Principal Investigator will consult with the IRB to conduct the review and make the decision to resume or close the study for any SAEs that meet the criteria for pausing the study. As part of the pause review, the reviewers will also advise on whether the study needs to be paused again for any subsequent adverse events of the same type. When indicated, safety data reports and changes in study status will be submitted to the IRB and FDA in accordance with institutional policy.

9. ADVERSE EVENTS: LIST AND REPORTING REQUIREMENTS

9.1 Definitions

Adverse event: Any untoward medical occurrence associated with the use of the drug in humans, whether or not considered drug-related.

Suspected adverse reaction: Any adverse event for which there is a reasonable possibility that the drug caused the adverse event (considered "possibly related"). For the purposes of safety reporting, "reasonable possibility" means there is evidence to suggest a causal relationship between the drug and the adverse event. Suspected adverse reaction implies a lesser degree of certainty about causality than adverse reaction, which means any adverse event caused by a drug.

Serious Adverse Event: Any untoward medical occurrence associated with the use of a drug in humans, whether or not considered drug related. Serious adverse events are defined by federal regulations and include events which:

- Are fatal or life threatening
- Result in significant or persistent disability
- Require or prolong inpatient hospitalization
- Result in a congenital anomaly or neoplasm
- Result from an overdose

- Are other conditions which in the judgment of the PI represents a significant hazard

Life-threatening, suspected adverse reaction: A suspected adverse reaction is considered “life-threatening” if, in the view of the Investigator (i.e., the study site principal investigator), its occurrence places the patient or research subject at immediate risk of death. It does not include a suspected adverse reaction that had it occurred in a more severe form, might have caused death.

Unexpected, suspected adverse reaction: A suspected adverse reaction is considered “unexpected” if it is not listed in the general investigational plan or clinical protocol, or is not listed at the specificity or severity that has been previously observed and/or specified. If an investigator brochure is not required or available, suspected adverse reaction is not consistent with the risk information described in the general investigational plan or elsewhere in the current application, as amended. "Unexpected," as used in this definition, also refers to adverse events or suspected adverse reactions that are mentioned in the investigator brochure as occurring with a class of drugs or as anticipated from the pharmacological properties of the drug, but are not specifically mentioned as occurring with the particular drug under investigation. Any clinically important increase in the rate of a serious suspected adverse reaction over that listed in the protocol or investigator brochure can also be considered unexpected. An aggregate analysis of specific events observed in a clinical trial (such as known consequences of the underlying disease or condition under investigation or other events that commonly occur in the study population independent of drug therapy) that indicates those events occur more frequently in the drug treatment group than in a concurrent or historical control group.

All observed or volunteered adverse events (serious or non-serious), regardless of a study group or suspected causal relationship to the study drug(s) will be recorded in the subjects’ case histories. For all adverse events, sufficient information will be pursued and/or obtained so as to permit 1) an adequate determination of the outcome of the event (i.e., whether the event should be classified as a serious adverse event) and 2) an assessment of the causal relationship between the adverse event and the study drug(s). All toxicities encountered during the study will be evaluated on an ongoing basis, according to the NCI Common Toxicity Criteria version 5.0.

9.2 Expected Adverse Events

9.2.1 Related to phlebotomy, bone marrow examination, and research sample collection

Grade 1-2 of the following signs and symptoms are expected in phlebotomy and research sample collection:

1. Minor bleeding and bruising at the site of biospecimen collection
2. Minor infection at the site of biospecimen collection
3. Scar at the site of biospecimen collection
4. Pain or itching at the site of biospecimen collection
5. Light-headedness, fainting, and nausea due to vasovagal reactions

9.2.2 Related to ACTIMMUNE® (interferon gamma-1b)

The expected adverse events related to ACTIMMUNE® (interferon gamma-1b) are

described in the prescribing information.[33]

9.2.3 Related to donor lymphocyte infusion

The following symptoms and signs are expected adverse events associated with donor lymphocyte infusion.

1) Acute GVHD (up to 61 %)
2) Chronic GVHD
3) Bone marrow aplasia
4) Infusion reaction

9.2.4 Related to allogeneic stem cell transplantation

The following symptoms and signs are expected adverse events for the transplant recipient

1) Renal insufficiency
2) Hepatic insufficiency
3) Transient cardiac arrhythmias
4) Transient cardiac insufficiency
5) Pulmonary insufficiency
6) Neutropenia and its complications
7) Thrombocytopenia and its complications
8) Anemia and its complications
9) Treatable infections from bacteria, viruses, protozoa and fungi
10) Late effects of transplant regimens including chronic fatigue, cataracts, infertility, growth impairment, hypothyroidism, bone complications, and dental caries
11) Headache, insomnia, psychosis, mood changes, disorientation, seizures from metabolic imbalance
12) Nausea, vomiting, diarrhea, mucositis, weight loss, dry mouth, hiccoughs, constipation
13) Well-characterized drug reactions - allergic manifestations, "red man" syndrome, steroid effects
14) Well-characterized drug side effects from drugs used routinely in transplant recipients (e.g.; immunosuppressive drugs, antimicrobials)
15) Common side effects of antiemetics, analgesics, anti-inflammatory agent and known complications of steroid therapy
16) Complications from intravenous catheters, thrombotic occlusion, infection, local reactions, cardiac arrhythmia

9.3 Reporting of Serious Adverse Events

All events meeting the definition of a serious adverse event should be reported according to the departmental SAE checklist and SAE form. The initial SAE form should be sent to the following within 24 hours / 1 business day of the Principal Investigator becoming aware:

1. Investigator-Sponsor
2. crssafetysubmissions@upmc.edu
3. Local Institutional Review Board when reporting requirements are met.

4. Horizon –
   Email: AdverseEvents@horizonpharma.com
   Fax: 1-800-860-7836

In addition to completing appropriate patient demographic and suspect medication information, the report should include as applicable the following information that is available at the time of report within the Sections B and C of the departmental SAE form:

- CTCAE v5.0 term(s) and grade(s)
- current status of study drug
- all interventions to address the AE (testing and result, treatment and response)
- hospitalization and/or discharge dates
- event relationship to study drug

Follow-up reports:

Additional information may be added to a previously submitted report by adding to the original departmental SAE form and submitting it as follow-up or creating supplemental summary information and submitting it as follow-up with the original departmental SAE form.

9.4 Reporting adverse events to the responsible IRB

In accordance with applicable policies of the University of Pittsburgh Institutional Review Board (IRB), the PI will report to the IRB any observed or volunteered adverse event that is determined to be 1) associated with the investigational drug or study treatment(s); 2) a serious adverse event (SAE, described in section 9.1); or 3) an unexpected, suspected adverse reaction (defined in section 9.1). Adverse event reports will be submitted to the IRB in accordance with the respective IRB procedures.

Applicable adverse events will be reported to the IRB as soon as possible and, in no event, later than 10 calendar days following the investigator's receipt of the respective information. Adverse events which are 1) associated with the investigational drug or study treatment(s); 2) fatal or life-threatening; and 3) unexpected will be reported to the IRB within 24 hours of the investigator's receipt of the respective information.

Follow-up information to a reported adverse event will be submitted to the IRB as soon as the relevant information is available. If the results of the investigator's follow-up investigation show that an adverse event that was initially determined to not require reporting to the IRB does, in fact, meet the requirements for reporting, the investigator will report the adverse event to the IRB as soon as possible, but in no event later than 10 calendar days, after the determination was made.

Expected AEs listed in section 9.2 will not be reported but will be described in a case report form and summarized in the annual report of the protocol.

Adverse events related to the subject's pre-existing or undiagnosed conditions or concurrent therapies or medication will not be collected or reported.

9.5 Time Period for Collecting Adverse Events

Collection of adverse events will begin after the subject sign the informed consent form and will end at either time point described below, whichever occurs later

For subjects who completed the IFN-γ therapy: At the protocol visit #8: 28 days (+/- 5 days) safety assessment after the last dose of IFN-γ therapy (Off treatment assessment) defined on section 6.5.9

For subject who meet the criteria for removal from the study: At off study visit as defined on section 8.3.2.

10. DATA MANAGEMENT PLAN

10.1 Clinical Data Management

All human subjects personally identifiable information (PII) as defined in accordance with the Health Insurance Portability and Accountability Act, eligibility, and consent verification will be recorded in CTMA. Primary data obtained during the conduct of the protocol will be kept in secure network drives or in approved alternative sites that comply with UPMC/ University of Pittsburgh security standards. At Fred Hutchinson Cancer Research Center, primary data will be kept in their secure network drives that comply with the security standards of Fred Hutchinson Cancer Research Center. Deidentified data in electronic case report form (eCRF) will be then shared using a secure web-based SharePoint that complies with UPMC/University of Pittsburgh security standards.

10.2 Genomic Data Management

The coded sequenced data will be stored in a password-protected secure database that complies with UPMC/ University of Pittsburgh security standards.

10.3 End of Study Procedures

Data will be stored in locked cabinets or in a password protected database until it is no longer of scientific value.

10.4 Loss or Destruction of Data

Should we become aware that a major breach in our plan to protect patient confidentiality and trial data has occurred, the IRB will be notified.

Any breaches of confidentiality will be brought to the attention of the principal investigator and co-investigators for analysis and action. Data reviewed and discussed during these meetings will be kept confidential. Any breaches in subject confidentiality will be reported to the IRB.

10.5 Incidental/Secondary Findings Disclosure Procedure

Nucleotide sequence variations to be determined in these studies are not known to be associated with any known disease, and the research laboratory test used to identify the sequencing is not the Clinical Laboratory Improvement Amendments (CLIA)-certified for clinical testing. For all these reasons, the genetic information obtained <u>cannot be meaningfully</u>

interpreted outside the narrow focus of the study and will not be provided to the subjects or their physicians.

11. RISK AND BENEFIT

As described in the introduction, post-transplant relapse harbors very high risk of mortality even with current best standard of care (greater than 80-90% mortality in 2 years). In addition, alloSCT itself entails serious discomforts and hazards for the patient since pre-transplant conditioning regimen and alloreactive T cells can predispose the subjects to suffer from various adverse events (GVHD, infections, and organ toxicity) any time after alloSCT irrespective to the study intervention. The investigational component of this transplant protocol ultimately aims to improve graft versus leukemia effect and overall survival; however, IFN-γ may theoretically induce GVHD after alloSCT especially when concurrently used with donor lymphocyte infusions. Clinically, this approach is ethically acceptable because we will target a patient group with hematologic malignancies for which no therapy offers a greater than 10% chance of long-term survival. Subjects will make an informed decision to opt for a procedure which they understand has not been studied before, but which has a strong preclinical rationale. Risks of potential toxicity from IFN-γ will be fully explained in the consent. Implicit in this decision is the opportunity to weigh all treatment options after full information has been given to them by the research team and the referring physician. Therefore, for the adult subjects on this protocol, the research involves greater than minimal risk to subjects with the prospect of direct benefit (45 CFR 46.102).

The IRB will be notified of any change in risk/benefit ratio affecting decisions about study continuation. If any literature becomes available, which suggests that conducting this trial is no longer ethical, the study will be terminated, and the IRB will be notified of the new findings.

Minimal risks and greater than minimal risks related to this protocol are listed in sections 11.1–11.5.

11.1 Risks Related to Interferon-gamma (greater than minimal risk)

Anticipated risks from interferon-gamma are summarized in section 9.2.2. Additional risk in this protocol includes the potential for de novo or exacerbation of acute or chronic GVHD, flu-like symptoms, auto (allo-) immune disease, and organ toxicity.

11.2 Risks Related to Saliva Collection (negligible risk)

No major or minor risks are involved with saliva collection.

11.3 Risks Related to Phlebotomy or Bone Marrow Examination (minimal risk)

No major risks are involved with blood removal by intravenous puncture or bone marrow aspiration. Minor complications include bleeding, pain, itchiness, hematoma formation at the phlebotomy or bone marrow aspiration site, light-headedness, fainting, and nausea due to vasovagal reactions, and local infection.

11.4 Risks Related to Nucleotide Sequencing (minimal risk)

Genetic information can have implications regarding health, identity, paternity, employability, or insurability for the research participant as well as family members. No such relationship will be known to exist for any of the genes we are testing or will test. The nature of the genetic testing detailed in the protocol will not put participants at any further risk. Nucleotide sequence variations to be determined in this study are not known to be associated with any known diseases. Genetic information revealed to the investigators cannot be meaningfully interpreted outside the narrow focus of the research and will not be provided to the healthy volunteers or their physicians.

11.5 Risks Related to Protection of Confidentiality (minimal risk)

As with all research information, the information from this study will be kept confidential. Samples will be assigned a unique code prior to DNA testing, which will serve as a link to the individual's identity and other information collected as part of this research protocol. The actual DNA testing will be performed on coded samples, ensuring collaborating investigators (UPMC Genome Center Lab) will be blinded to the identity of the sample donor. The DNA information will be entered into a password-protected database.

12. COST AND PAYMENT

12.1 Payment and Compensation

The subjects who participate in this protocol will not receive any compensation related to study intervention or biospecimen collection. If they incur any costs related to complications of study participation, their insurance companies will be charged for their medical care. This will be explicitly stated in the consent documents.

13. DATA SAFETY MONITORING PLAN

Investigator/Sub-investigators, regulatory, CRS management, clinical research coordinators, clinical research associates, data managers, and clinic staff meet regularly in disease center Data Safety Monitoring Boards (DSMB) to review and discuss study data to include, but not limited to, the following:

- serious adverse events
- subject safety issues
- recruitment issues
- accrual
- protocol deviations
- unanticipated problems
- breaches of confidentiality

Minutes from the disease center DSMB meetings are available to those who are unable to attend in person.

All toxicities encountered during the study will be evaluated on an ongoing basis according to the NCI Common Toxicity Criteria Version 5.0. All study treatment associated adverse events

that are serious, at least possibly related and unexpected will be reported to the IRB. Any modifications necessary to ensure subject safety and decisions to continue or close the trial to accrual are also discussed during these meetings. If any literature becomes available which changes the risk/benefit ratio or suggests that conducting the trial is no longer ethical, the IRB will be notified in the form of an Unanticipated Problem submission and the study may be terminated.

All study data reviewed and discussed during these meetings will be kept confidential. Any breach in subject confidentiality will be reported to the IRB in the form of an Unanticipated Problem submission. The summaries of these meetings are forwarded to the UPMC Hillman Cancer Center DSMC which also meets monthly following a designated format.

For all research protocols, there will be a commitment to comply with the IRB's policies for reporting unanticipated problems involving risk to subjects or others (including adverse events). DSMC progress reports, to include a summary of all serious adverse events and modifications, and approval will be submitted to the IRB at the time of renewal.

Protocols with subjects in long-term (survival) follow-up or protocols in data analysis only, will be reviewed bi-annually.

Both the UPMC Hillman Cancer Center DSMC as well as the individual disease center DSMB have the authority to suspend accrual or further investigate treatment on any trial based on information discussed at these meetings.

All records related to this research study will be stored in a locked environment. Only the researchers affiliated with the research study and their staff will have access to the research records.

14. REFERENCES

1. Bleakley M, Riddell SR. Molecules and mechanisms of the graft-versus-leukaemia effect. *Nat Rev Cancer*. 2004;4(5):371-380.
2. Horowitz M, Schreiber H, Elder A, et al. Epidemiology and biology of relapse after stem cell transplantation. *Bone Marrow Transplant*. 2018.
3. Horowitz MM, Gale RP, Sondel PM, et al. Graft-versus-leukemia reactions after bone marrow transplantation. *Blood*. 1990;75(3):555-562.
4. Mackinnon S, Papadopoulos EB, Carabasi MH, et al. Adoptive immunotherapy evaluating escalating doses of donor leukocytes for relapse of chronic myeloid leukemia after bone marrow transplantation: separation of graft-versus-leukemia responses from graft-versus-host disease. *Blood*. 1995;86(4):1261-1268.
5. Porter DL, Roth MS, McGarigle C, Ferrara JL, Antin JH. Induction of graft-versus-host disease as immunotherapy for relapsed chronic myeloid leukemia. *N Engl J Med*. 1994;330(2):100-106.
6. Collins RH, Jr., Shpilberg O, Drobyski WR, et al. Donor leukocyte infusions in 140 patients with relapsed malignancy after allogeneic bone marrow transplantation. *J Clin Oncol*. 1997;15(2):433-444.
7. Schmid C, Labopin M, Nagler A, et al. Donor lymphocyte infusion in the treatment of first hematological relapse after allogeneic stem-cell transplantation in adults with acute myeloid leukemia: a retrospective risk factors analysis and comparison with other strategies by the EBMT Acute Leukemia Working Party. *J Clin Oncol*. 2007;25(31):4938-4945.
8. Matte CC, Cormier J, Anderson BE, et al. Graft-versus-leukemia in a retrovirally induced murine CML model: mechanisms of T-cell killing. *Blood*. 2004;103(11):4353-4361.
9. Matte-Martone C, Liu J, Jain D, McNiff J, Shlomchik WD. CD8+ but not CD4+ T cells require cognate interactions with target tissues to mediate GVHD across only minor H antigens, whereas both CD4+ and CD8+ T cells require direct leukemic contact to mediate GVL. *Blood*. 2008;111(7):3884-3892.
10. Matte-Martone C, Liu J, Zhou M, et al. Differential requirements for myeloid leukemia IFN-gamma conditioning determine graft-versus-leukemia resistance and sensitivity. *J Clin Invest*. 2017;127(7):2765-2776.
11. Elmaagacli AH, Steckel NK, Koldehoff M, et al. Early human cytomegalovirus replication after transplantation is associated with a decreased relapse risk: evidence for a putative virus-versus-leukemia effect in acute myeloid leukemia patients. *Blood*. 2011;118(5):1402-1412.
12. Foley B, Cooley S, Verneris MR, et al. Cytomegalovirus reactivation after allogeneic transplantation promotes a lasting increase in educated NKG2C+ natural killer cells with potent function. *Blood*. 2012;119(11):2665-2674.
13. Christopher MJ, Petti AA, Rettig MP, et al. Immune Escape of Relapsed AML Cells after Allogeneic Transplantation. *N Engl J Med*. 2018.
14. Toffalori C, Zito L, Gambacorta V, et al. Immune signature drives leukemia escape and relapse after hematopoietic cell transplantation. *Nat Med*. 2019;25(4):603-611.
15. Devane JG, Martin ML, Matson MA. A short 2 week dose titration regimen reduces the severity of flu-like symptoms with initial interferon gamma-1b treatment. *Curr Med Res Opin*. 2014;30(6):1179-1187.
16. Zorn E, Mohseni M, Kim H, et al. Combined CD4+ donor lymphocyte infusion and low-dose recombinant IL-2 expand FOXP3+ regulatory T cells following allogeneic hematopoietic stem cell transplantation. *Biol Blood Marrow Transplant*. 2009;15(3):382-388.
17. Romee R, Cooley S, Berrien-Elliott MM, et al. First-in-human phase 1 clinical study of the IL-15 superagonist complex ALT-803 to treat relapse after transplantation. *Blood*.

2018;131(23):2515-2527.
18. Andrea S. Henden AV, Justine Leach , Elise Sturgeon , Judy Avery , Jessica Kelly , Stuart Olver , Luke Samson , Gunter Hartel , Simon Durrant , Jason Butler , Anthony J. Morton , Ashish Misra , Siok-Keen Tey , Elango Subramoniapillai , Cameron Curley , Glen Kennedy , Geoffrey R. Hill. Pegylated interferon-2α invokes graft-versus-leukemia effects in patients relapsing after allogeneic stem cell transplantation. *Blood Adv* 2019;3(20):3013.
19. Passweg JR, Tiberghien P, Cahn JY, et al. Graft-versus-leukemia effects in T lineage and B lineage acute lymphoblastic leukemia. *Bone Marrow Transplant*. 1998;21(2):153-158.
20. Lee SJ, Klein JP, Barrett AJ, et al. Severity of chronic graft-versus-host disease: association with treatment-related mortality and relapse. *Blood*. 2002;100(2):406-414.
21. Kolb HJ, Schmid C, Barrett AJ, Schendel DJ. Graft-versus-leukemia reactions in allogeneic chimeras. *Blood*. 2004;103(3):767-776.
22. Drobyski WR, Keever CA, Roth MS, et al. Salvage immunotherapy using donor leukocyte infusions as treatment for relapsed chronic myelogenous leukemia after allogeneic bone marrow transplantation: efficacy and toxicity of a defined T-cell dose. *Blood*. 1993;82(8):2310-2318.
23. Kolb HJ, Schattenberg A, Goldman JM, et al. Graft-versus-leukemia effect of donor lymphocyte transfusions in marrow grafted patients. European Group for Blood and Marrow Transplantation Working Party Chronic Leukemia. *Blood*. 1995;86(5):2041-2050.
24. Porter DL, Collins RH, Jr., Hardy C, et al. Treatment of relapsed leukemia after unrelated donor marrow transplantation with unrelated donor leukocyte infusions. *Blood*. 2000;95(4):1214-1221.
25. Porter DL, Roth MS, McGarigle C, Ferrara JL, Antin JH. Induction of graft-versus-host disease as immunotherapy for relapsed chronic myeloid leukemia. *New England Journal of Medicine*. 1994;330(2):100-106.
26. Salama M, Nevill T, Marcellus D, et al. Donor leukocyte infusions for multiple myeloma. *Bone Marrow Transplant*. 2000;26(11):1179-1184.
27. Kharfan-Dabaja MA, Labopin M, Polge E, et al. Association of Second Allogeneic Hematopoietic Cell Transplant vs Donor Lymphocyte Infusion With Overall Survival in Patients With Acute Myeloid Leukemia Relapse. *JAMA Oncol*. 2018;4(9):1245-1253.
28. Miller JS, Warren EH, van den Brink MR, et al. NCI First International Workshop on The Biology, Prevention, and Treatment of Relapse After Allogeneic Hematopoietic Stem Cell Transplantation: Report from the Committee on the Biology Underlying Recurrence of Malignant Disease following Allogeneic HSCT: Graft-versus-Tumor/Leukemia Reaction. *Biol Blood Marrow Transplant*. 2010;16(5):565-586.
29. Alyea EP, DeAngelo DJ, Moldrem J, et al. NCI First International Workshop on The Biology, Prevention and Treatment of Relapse after Allogeneic Hematopoietic Cell Transplantation: report from the committee on prevention of relapse following allogeneic cell transplantation for hematologic malignancies. *Biol Blood Marrow Transplant*. 2010;16(8):1037-1069.
30. de Lima M, Porter DL, Battiwalla M, et al. Proceedings from the National Cancer Institute's Second International Workshop on the Biology, Prevention, and Treatment of Relapse After Hematopoietic Stem Cell Transplantation: part III. Prevention and treatment of relapse after allogeneic transplantation. *Biol Blood Marrow Transplant*. 2014;20(1):4-13.
31. Niederwieser D, Baldomero H, Szer J, et al. Hematopoietic stem cell transplantation activity worldwide in 2012 and a SWOT analysis of the Worldwide Network for Blood and Marrow Transplantation Group including the global survey. *Bone Marrow Transplant*. 2016;51(6):778-785.
32. NCI-SEER-Database. http://seer.cancer.gov/statfacts/index.html. 2014.

33. Horizon Therapeutics USA I. Prescribing information for ACTIMMUNE® (interferon gamma-1b) injection. 2019.

15. APPENDIX

15.1 Appendix A. CIBMTR acute GVHD grading and staging system

GVHD Grading and Staging

**Extent of Organ Involvement**			
**Stage**	**Skin**	**Liver**	**Gut**
1	Rash on <25% of skin[1]	Bilirubin 2-3 mg/dl[2]	Diarrhea > 500 ml/day[3] or persistent nausea[4]<br>*Pediatric*: 280-555 ml/m^2/day or 10-19.9 mL/kg/day
2	Rash on 25-50% of skin	Bilirubin 3-6 mg/dl	Diarrhea >1000 ml/day<br>*Pediatric*: 556-833 ml/m^2/day or 20-30 mL/kg/day
3	Rash on >50% of skin	Bilirubin 6-15 mg/dl	Diarrhea >1500 ml/day<br>*Pediatric*: >833 ml/m^2/day or > 30 mL/kg/day
4	Generalized erythroderma with bullous formation	Bilirubin >15 mg/dl	Severe abdominal pain, with or without ileus, and / or grossly blood stool
**Grade[5]**			
I	Stage 1-2	None	None
II	Stage 3	Stage 1	Stage 1
III	—	Stage 2-3	Stages 2-4
IV[6]	Stage 4	Stage 4	—

[1] Use “Rule of Nines” (see Percent Body Surfaces table below) or burn chart to determine extent of rash.

[2] Range given as total bilirubin. Downgrade one stage if an additional cause of elevated bilirubin has been documented.

[3] Volume of diarrhea applies to adults. For pediatric patients, the volume of diarrhea should be based on body surface area. Downgrade one stage if an additional cause of diarrhea has been documented.

[4] Persistent nausea with or without histologic evidence of GVHD in the stomach or duodenum.

[5] Criteria for grading given as minimum degree of organ involvement required to confer that grade.

[6] Grade IV may also include lesser organ involvement with an extreme decrease in performance status

15.2 Appendix B. NIH chronic GVHD scoring system

Organ Scoring of Chronic GVHD

Organ	Score 0	Score 1	Score 2	Score 3
**Skin % BSA[1]**	No BSA invo lved	1-18% BSA	19-50% BSA	>50% BSA
**Skin Features**	No sclerotic features	N/A	Superficial sclerotic features, but not "hidebound"	Deep sclerotic features; "hidebound;" impaired mobility; ulceration
**Mouth**	No symptoms	Mild symptoms **with** dise ase signs but not limiting oral intake significantly	Moderate symptoms with disease signs **with** partial limitation of oral intake	Severe symptoms with disease signs **with** major limitation of oral intake
**Eyes**	No symptoms	Mild dry eye symptoms not affecting ADL (requi rement of lubricant drops ≤ 3x/day)	Moderate dry eye symptoms partially affecting ADL (re quiring lubricant drops > 3x/day or punctal	Severe dry eye symptoms significantly affecting ADL (special eyewear to relieve pain) **OR** unable to work because of ocular

			plugs) **WITHOUT** new vision impairment due to keratoconjunctivitis sicca (KCS)	symptoms **OR** loss of vision due to keratoconjunctivitis sicca (KCS)
**GI Tract**	No symptoms	Symptoms without significant weight loss (< 5%)	Symptoms associated with mild to moderate weight loss (5-15%) within 3 months **OR** moderate diarrhea without significant interference with daily living	Symptoms associated with significant weight loss (> 15%) within 3 months, requires nutritional supplement for most calorie needs **OR** esophageal dilation **OR** severe diarrhea with significant interference with daily living.
**Liver**	Normal total bilirubin and ALT or AP < 3 x ULN	Normal total bilirubin with ALT ≥ 3 to 5 x ULN or AP ≥ 3 x ULN	Elevated total bilirubin but ≤ 3 mg / dL or ALT > 5 x ULN	Elevated total bilirubin > 3 mg / dL
**Lungs Symptom Score:**	No symptoms	Mild symptoms (SOB after climbing one flight of steps)	Moderate symptoms (SOB after walking on flat ground)	Severe symptoms (SOB at rests; requires O2)
**Lungs Lung Score:**	FEV1 ≥ 80%	FEV1 60-79%	FEV1 40-59%	FEV1 ≤ 39%
**Joints and Fascia**	No symptoms	Mild tightness of arms or legs, normal or mild decreased range of motion **AND** not affecting ADL	Tightness of arms or legs **OR** joint contractures, erythema thought to be due to fasciitis, moderate decrease of range of motion **AND** mild	Contractures **WITH** significant decrease of range of motion **AND** significant limitation of ADL (unable to tie shoes, button shirts, dress self, etc.)

			to moderate limitation of ADL	
**Genital Tract**[2]	No signs	Mild signs and females with or without discomfort on exam	Moderate signs and may have signs of discomfort on exam	Severe signs with or without symptoms
**Other Features**[3]	No GVHD	Mild	Moderate	Severe

NIH Consensus Criteria, 2014

[1] Features to be scored by BSA: Maculopapular rash, lichen planus-like features, sclerotic features, papulosquamous lesions or ichthyosis, keratosis pilaris-like GVHD.

[2] Scoring is based on severity of the signs instead of symptoms, based on limited available data and the opinions of experts. Female or male genital GVHD is not scored if a practitioner is unable to examine the patient.

[3] May include ascites, pericardial effusion, pleural effusion(s), nephrotic syndrome, myasthenia gravis, peripheral neuropathy, polymyositis, weight loss without GI symptoms, eosinophilia > 500/μL, platelets < 100,000/μL, others.

Skin: Ranges from skin discoloration to severe scarring and tightness. Includes, but is not limited to:

- Sclerosis: thickening of the skin, which may cause loss of suppleness
- Maculopapular rash / erythema: reddish skin with small confluent bumps / redness
- Lichen planus-like features: erythematous / violaceous flat-topped papules or plaques with or without surface reticulations or a silvery or shiny appearance.
- Papulosquamous lesions or ichthyosis: dry, scaly, or thickened skin
- Keratosis pilaris: small acne-like bumps and rough patches
- Poikiloderma: atrophy, pigmentary changes, and telangiectasia

If any skin abnormalities were present, but explained entirely by non-GVHD causes, specify any documented causes.

Mouth: Refers to white plaques, scarring, and ulcers occurring in the mouth and throat.

- Lichen planus-like features: whitish lacy patches that usually appear first on inner cheeks, but can involve roof of mouth, gums, and / or tongue

If any mouth abnormalities were present, but explained entirely by non-GVHD causes, specify any documented causes.

Eyes: Recipients may have dry eyes and corneal ulcers due to keratoconjunctivits sicca.

- Keratoconjuctivitis sicca (KCS): dry eye syndrome

If any eye abnormalities were present, but explained entirely by non-GVHD causes, specify documented causes.

Gastrointestinal tract (GI):

- Esophageal web / proximal stricture or ring: extension of esophageal tissue
- Dysphagia: difficulty swallowing
- Anorexia
- Nausea
- Vomiting
- Diarrhea
- Weight loss: weight loss ≥ 5%
- Failure to thrive

If any GI abnormalities were present, but explained entirely by non-GVHD causes, specify documented causes.

Liver: Record all types of liver abnormalities either clinical or histological.

- Liver involvement may be manifested by elevation of any of the liver function tests (bilirubin, particularly the direct component: alkaline phosphatase; GGT; SGOT [AST]; SGPT [ALT]).

If any liver abnormalities were present, but explained entirely by non-GVHD causes, specify documented causes.

Lung: This ranges from mild impairment on pulmonary function tests to severe disorders.

If pulmonary function tests were performed, specify FEV1 percent.

If any lung abnormalities were present, but explained entirely by non-GVHD documented causes, specify causes.

Joints and fascia:

- Contractures: loss of joint mobility due to skin or fascia changes

If any joint or fascia abnormalities were present, but explained entirely by non-GVHD causes, specify causes.

Genital tract:

- Female: Vaginitis / stricture: pain, ulceration, inflammation, eventually scarring / narrowing of the vaginal opening.
- Male: Pain, burning sensation, lichen planus or lichen sclerosis features, scarring, stenosis.

If any genital tract abnormalities were present, but explained entirely by non-GVHD causes, specify documented causes.

15.3 Appendix C. Schedule of event for cohort 1

	Screening	Stage 1			Stage 2					
Visit	Pre	V1	V2	V3	V4	V5	V6	V7	V8	V9
Days	-14 to -1	0	2	14	28	42	56	84	112	180
Window (days)			+5	+/-5	+/-5	+/-5	+/-5	+/-5	+/-5	+/-14
Enrollment procedure										
Eligibility assessment	X									
Informed consent	X									
Pre-treatment assessment										
H&P, performance status	X									
History of GVHD	X									
History of alloSCT	X									
Bone marrow examination	X*1)									
Pregnancy test	X									
CBC, chemistry, and hepatic panel	X									
STR chimerism	X									
Safety and efficacy assessment										
Vital sign		X	X	X	X	X	X	X	X	X
History and physical exam		X	X	X	X	X	X	X	X	X
CBC, chemistry, and hepatic panel				X	X	X	X	X	X	X
Bone marrow examination		X*1)	X		X			X		X
STR chimerism					X			X		X
Treatment										
IFN-γ (Actimmune) per protocol		X	X	X	X	X	X			
Donor lymphocyte infusion					X		X*2)	X*3)		
Research sample collection										
Bone marrow aspirate (10mL)	X*1)	X*1)	X		X			X		X*4)
Peripheral blood (30ml)		X	X		X		X	X	X	X

*1) No need to repeat bone marrow if the research sample was collected during the screening period
*2) Only applicable if the second dose of DLI is feasible
*3) Only applicable if the third dose of DLI is feasible
*4) This bone marrow is optional and can be collected if clinically indicated.

15.4 Appendix D. Schedule of event for cohort 2

	Screening	Stage 1		Stage 2						
Visit	Pre	V1	V2	V3	V4	V5	V6	V7	V8	V9
Days	-14 to -1	0	2	7	21	35	56	84	112	180
Window (days)			+5	-3 to +5	+/-5	+/-5	+/-7	+/-14	+/-14	+/-28
Enrollment procedure										
Eligibility assessment	X									
Informed consent	X									
Pre-treatment assessment										
H&P, performance status	X									
History of GVHD	X									
History of alloSCT	X									
Bone marrow examination	X*1)									
Pregnancy test	X									
CBC, chemistry, and hepatic pan	X									
STR chimerism	X									
Safety and efficacy assessment										
Vital sign		X	X	X	X	X	X	X	X	X
History and physical exam		X	X	X	X	X	X	X	X	X
CBC, chemistry, and hepatic panel				X	X	X	X	X	X	X
Bone marrow examination		X*1)	X					X		X
STR chimerism		X				X		X		X
Treatment										
IFN-γ (Actimmune) per protocol		X	X	X	X	X	X			
Donor lymphocyte infusion				X		X*2)	X*3)			
Research sample collection										
Bone marrow aspirate (10mL)	X*1)	X*1)	X			X*4)	X*4)	X		X*4)
Peripheral blood (30ml)		X	X			X	X	X	X	X

*1) No need to repeat bone marrow if the research sample was collected during the screening period
*2) Only applicable if the second dose of DLI is feasible
*3) Only applicable if the third dose of DLI is feasible
*4) This bone marrow is optional and can be collected if clinically indicated.
